# General Perturb-Then-Diagonalize Model for the Vibrational Frequencies and Intensities of Molecules Belonging to Abelian and Non-Abelian Symmetry Groups

**DOI:** 10.1021/acs.jctc.1c00240

**Published:** 2021-06-04

**Authors:** Marco Mendolicchio, Julien Bloino, Vincenzo Barone

**Affiliations:** Scuola Normale Superiore, Piazza dei Cavalieri 7, I-56126 Pisa, Italy

## Abstract

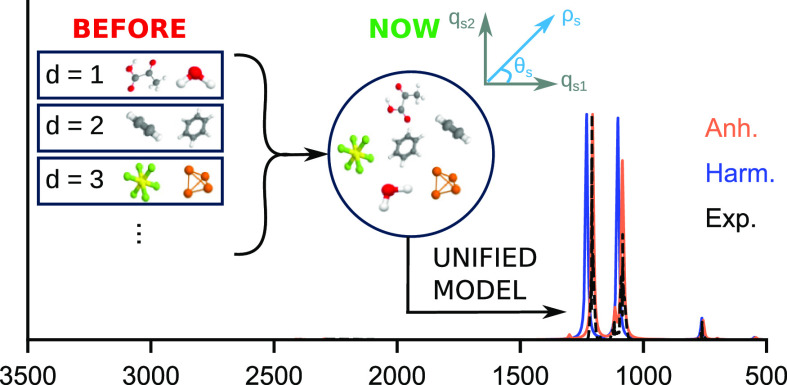

In this paper, we show that the standard second-order vibrational perturbation theory (VPT2) for Abelian groups can be used also for non-Abelian groups without employing specific equations for two- or threefold degenerate vibrations but rather handling in the proper way all the degeneracy issues and deriving the peculiar spectroscopic signatures of non-Abelian groups (e.g., -doubling) by a posteriori transformations of the eigenfunctions. Comparison with the results of previous conventional implementations shows a perfect agreement for the vibrational energies of linear and symmetric tops, thus paving the route to the transparent extension of the equations already available for asymmetric tops to the energies of spherical tops and the infrared and Raman intensities of molecules belonging to non-Abelian symmetry groups. The whole procedure has been implemented in our general engine for vibro-rotational computations beyond the rigid rotor/harmonic oscillator model and has been validated on a number of test cases.

## 1 Introduction

The reliability of quantum chemical (QC) models to support experimental findings is related from one side to their accuracy and from the other side to their feasibility, robustness, and ease of use.^1,2^ Concerning the accuracy, electronic structure computations of energies, geometries, and force fields are nowadays able to rival high-resolution spectroscopy for small systems and to help assignments and interpretation of all kinds of spectra for larger molecular systems, provided that nuclear motions and environmental effects are taken into proper account.^3−8^ In the present contribution, we will be concerned with molecular vibrations, which are directly sampled by different conventional [infrared (IR), Raman] and chiral (VCD, ROA) spectroscopies,^2,9^ but tune also the outcomes of other spectroscopies (e.g., distortion constants in microwave spectroscopy^4^ or line shapes in electronic spectroscopies^10^). Until quite recently, except for exceedingly small systems, the rigid rotor/harmonic oscillator model was nearly exclusively employed to describe molecular vibrations. However, the neglect of anharmonicity and ro-vibrational couplings can introduce significant errors, sometimes leading to even qualitatively wrong interpretations of experimental data.

Among the different approaches available to go beyond the rigid rotor/harmonic oscillator approximation,^11−35^ those based on perturbation theory applied to the expansion of the nuclear Hamiltonian in the power series of products of vibrational and rotational operators (hereafter referred to as vibrational perturbation theory, VPT) are particularly appealing for their remarkable cost/performance ratio, at least for semi-rigid molecular systems. Moreover, some formulations of VPT, such as the Van Vleck contact transformation method,^36^ fully justify a generalized model (GVPT2),^37,38^ allowing to couple the advantages of perturbative (for weakly coupled modes) and variational (for strongly coupled modes) treatments in a well-sound and robust framework. Actually, the GVPT2 approach belongs to the class of perturb-then-diagonalize many-body models, which, although less widely used than diagonalize-then-perturb models, have in the present context some appealing advantages from both conceptual and implementative points of view. Implementations of VPT2 approaches in general-purpose QC software are now quite widespread,^39−50^ although fully automatic and robust implementations of GVPT2 are less common. The situation is different for intensities of IR, Raman, VCD, and ROA spectra, which need to account for both mechanical and electrical/magnetic anharmonicity. To the best of our knowledge, the only general platform allowing GVPT2 computations for all the spectroscopic techniques mentioned above is the one implemented by some of the present authors in the Gaussian package.^51^ Extension of perturbative/variational procedures to the treatment of flexible systems is underway along different avenues including approaches based on reaction paths or surfaces following the decoupling of one or two large-amplitude motions from a bath of small-amplitude modes and/or the replacement of Cartesian coordinates by generalized internal coordinates.^52−55^

Here, we tackle a different problem, related to the treatment of molecular systems with non-Abelian point group symmetries, namely, linear, symmetric, and spherical tops. As a matter of fact, a significant ensemble of molecular systems, ranging from small to large sizes and of interest in various research fields, belong to these classes, including, for instance, organic and organometallic compounds like coronene and ferrocene^30,56−58^ or acetylene derivatives.^59−70^ The presence of degenerate modes raises multiple practical and theoretical issues, which have been only marginally addressed until now. A possible workaround is to lift the degeneracies by reducing the symmetry of the molecular system to the closest Abelian group symmetry,^42^ but this can lower the accuracy of the results. The rotational problem is actually simpler for non-Abelian groups because the rigid rotor approximation leads to analytical solutions, but a proper account of the degeneracy for the vibrational problem requires a careful check of the relative orientations of the degenerate modes to ensure that the anharmonic force fields and property surfaces are correctly built and an alternative derivation able to account for the couplings involving the degenerate modes. In a previous work,^71^ we have presented a complete framework implementing different equations for the vibrational frequencies based on the symmetry for linear and symmetric tops and taking into proper account both intrinsic and accidental degeneracies, leading to additional terms in the Hamiltonian or to singularities in the perturbative expansion, respectively. However, the current situation is unsatisfactory at several levels. First, the formulation derived from the work of Plíva^72^ cannot be extended straightforwardly to spherical tops, and to the best of our knowledge, no complete and correct derivation has been proposed. Second, the current implementation is particularly intricate due to the constant case switch depending on the symmetry and degeneracy of the modes in the calculation of the quantities of interest for the vibrational energies. Finally, it requires the use of complex algebra in the variational treatment, which leads to complex eigenvectors of the variational matrix, and thus the transition moments, even if the final intensities remain, of course, real.

Based on these premises, a unified treatment of Abelian and non-Abelian symmetries would be more efficient and general, provided that the results can be easily transformed to the more standard representation for compatibility and/or interpretative purposes. Furthermore, the simplicity of the new approach could allow a more straightforward implementation of new strategies based on VPT2^73^ not only for linear and symmetric tops but also for spherical tops. Finally, a general and robust framework can be set for the calculation of both IR and Raman intensities for all point groups without any need of complex algebra. This has convinced us to follow a different route, that is, to employ the asymmetric-top formulation also for the other cases, handling in the proper way all the degeneracy issues and deriving the customary spectroscopic signatures of non-Abelian groups (e.g., -type doubling) by a posteriori transformations of the eigenvectors. As will be shown in the following, the results for frequencies are exactly the same as those delivered by our previous conventional implementation (including resonance contributions), but we are now able to compute also intensities for both IR and Raman spectra.

The paper is organized as follows: In the Theory section, we start with deriving energy expressions for linear and symmetric tops, including resonance-free expressions for the zero-point energy, and then move to a general treatment of resonances and to intensities for all vibrational spectroscopies. The Results and Discussion section is organized in the same way but also considers different levels of electronic structure theory, starting from Hartree–Fock (HF) and second-order Møller–Plesset perturbation theory (MP2), which do not involve any underlying noise connected to numerical integration, and then moving to methods rooted in the density functional theory (DFT) up to double hybrids. The main results, remaining challenges, and perspectives are shortly outlined in the concluding section.

## 2 Theory

### 2.1 Framework

In order to set up the framework for our discussion, let us consider a system of *N* vibrational normal modes with a non-Abelian symmetry. These modes will generally be identified by the indexes *i*, *j*, *k*, *l*. Where relevant, and except if specified otherwise, different sets of indexes will be used to distinguish degenerate modes (*s*, *t*, *u*, followed by a subscript number for each mode) from the non-degenerate ones (*m*, *n*, *o*). As an example, modes *s*_1_ and *s*_2_ are two degenerate modes with the same harmonic wavenumber ω_s_, while *m* and *n* are two non-degenerate modes.

The formalism introduced by Plíva for symmetric and linear tops^72^ relies on a special set of coordinates for degenerate modes based on a complex combination
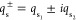
1where *q*_*i*_ represents the dimensionless normal coordinate associated to mode *i*. This form can actually be directly related to the definition of the harmonic vibrational wave function ψ. Thus, it is convenient to first recall its representation for a system with *N*′ non-degenerate modes and *N*″ sets of degenerate modes (*N*″ = 0 for asymmetric tops). In the canonical representation (*C*), ψ, associated to the vibrational state |***v***⟩, is given as a product of one-dimensional functions
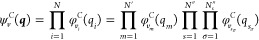
2where *N*_*s*_^″^ is the degeneracy order associated to degenerate mode *s*, *v*_*i*_ is the number of vibrational quanta corresponding to mode *i*, and  are the well-known one-dimensional harmonic oscillator wave functions. To make the discussion simpler, we will consider only molecules with at most doubly degenerate modes, that is, linear and symmetric tops. Extension to spherical tops is deferred to a dedicated section. Equation 2 can thus be written more explicitly as
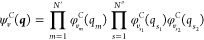
3

An alternative way to treat the degenerate coordinates is through the polar representation,^25^ in which the wave function, namely, ψ_*v*_^*P*^(***q***), assumes the following form:
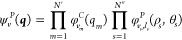
4where *v*_*s*_ and  are respectively the principal and angular quantum numbers, while  is the harmonic wave function of the two-dimensional isotropic harmonic oscillator Hamiltonian expressed in terms of the polar coordinates ρ_*s*_ and θ_*s*_ arising from a pair of degenerate modes.

It is worth recalling that both representations, canonical and polar, have the same eigenvalues and hence vibrational energies but different eigenstates. From a theoretical perspective, the polar representation is preferable since it leads to an explicit quantization of the vibrational angular momentum stemming from each pair of degenerate vibrations. The formulation proposed by Plíva takes these properties into account through a transformation of the degenerate normal coordinates and the associated force field.^72,74^

Having confirmed that the presence of degenerate modes does not preclude the development of VPT2 equations for symmetric and linear tops in the canonical representation (see Appendix A), the main task is to define a suitable transformation between the states obtained in each representation. At the harmonic level, it is possible to build a linear transformation between sets of degenerate states^38^
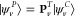
5where |ψ_*v*_^*P*^⟩ and |ψ_*v*_^*C*^⟩ are the column vectors containing the states with the same harmonic energy, which implies in practice that *v*_*m*_ and *v*_*s*_ = *v*_*s*_1__ + *v*_*s*_2__ are constant, and **P**_*v*_ is a unitary matrix connecting the two sets of states. The complete set of rotation matrices required for the conversion of fundamentals, first overtones, and binary (1 + 1) combinations from the vibrational ground state is reported in Section S1 of the Supporting Information. Furthermore, following the recent extension of our computational framework to the inclusion of three-quanta states,^75^ the full set of the corresponding rotations is reported for the sake of completeness.

Therefore, a unified framework can be set up, in which calculations are run in two steps:

1. anharmonic calculations in the canonical framework;
2. if degenerate modes are present, application of the necessary rotations to switch to the polar representation.

### 2.2 Vibrational Energies

Full derivations for the VPT2 energies of asymmetric^11,32,42^ and symmetric or linear^71,72,74^ tops have been reported elsewhere. Here, we will focus on the novel aspects using refs^48,71,76^ as a basis. Based on Plíva’s formalism, the vibrational energy for symmetric and linear tops can be written
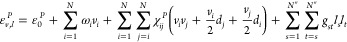
6where ε_0_^*P*^ is the zero-point vibrational energy (ZPVE) in the polar representation, *d*_*i*_ is the degeneration of the *i*th normal mode, and **χ**^*P*^ and **g** are respectively the anharmonic constant matrix in polar representation and the matrix containing the anharmonic contributions from the angular momenta, given in ref (71) and reported in Section S2 of the Supporting Information.

Let us consider a set of degenerate harmonic states in the canonical (|ψ_*v*_^*C*^⟩) and polar (|ψ_*v*_^*P*^⟩) representations. The associated blocks of the contact-transformed vibrational Hamiltonian are respectively **H̃**_*v*,*v*_^*C*^ and **H̃**_*v*,*v*_^*P*^, where the subscript “*v*” indicates that only matrix elements between states with the same principal quantum numbers *v* are included in the block. These blocks will be simply referred to as diagonal blocks in the following. By construction, only the diagonal elements of the corresponding matrices contribute to the anharmonic energies. Using eq 5, the following identity can be written
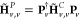
7

Since the trace of a matrix is invariant under similarity transformation, we have
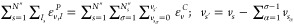
8

Equation 7 can thus be systematically used to obtain the energies in the polar representation, starting from both energies and resonances (see later) evaluated within the canonical representation.

After demonstrating that the expression of the resonance-free ZPVE for asymmetric tops^77−79^
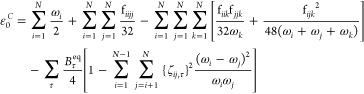
9can also be used for linear and symmetric tops (see Section S3 of the Supporting Information for details and notation), that is,
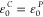
10

Equation 7 can be applied to calculate the energies in the polar representation for one- and two-quanta states involving at least one degenerate mode. Combining the rotation matrices reported in Section S1 of the Supporting Information with the transformation of the Hamiltonian given in eq 7, it is straightforward to prove that the anharmonic fundamental energies do not vary under the change of representation, a property true for any excited state involving only one degenerate mode excited with a single quantum (*v*_*s*_ = 1), for instance, , , , and so on.

Following the procedure outlined for the fundamental states, the rotation-based framework yields the expressions for the polar energies of the first overtone associated to a degenerate mode *s*
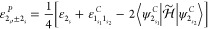
11a
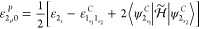
11bwhere  is the contact-transformed Hamiltonian,^24^ and
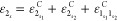
12as well as those of the binary (1+1) combination bands involving two degenerate modes, namely, *s* and *t*
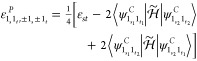
13a
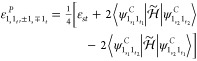
13bwhere
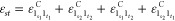
14

Contrary to fundamental bands, the energy terms in polar representation cannot be directly obtained from the canonical representation but require additional, off-diagonal terms, collectively called Darling–Dennison coupling terms, which will be illustrated later. Finally, three-quanta transitions are treated in the Section S1 of the Supporting Information.

### 2.3 Resonances and the Variational Correction

In the following, we will consider three models for the calculation of VPT2 energies. In the pure VPT2 approach (simply named VPT2), resonances are ignored, in the sense that all terms involved in the calculation of the quantities of interest are systematically included. As a result, this approach has a tendency to break down very quickly with the system size, often leading to unphysical results. An improvement is offered by the deperturbed VPT2 (DVPT2) scheme, where resonances are identified, usually through a multi-step procedure,^42,76,79,80^ and the related terms are discarded. Strong discrepancies are prevented, but the selective removal of terms can result in an unbalanced account of the anharmonic contributions, with varying intensity. In the most refined version, the generalized VPT2 (GVPT2) scheme,^32,42,76,81^ the resonant terms from DVPT2 are introduced back through an additional variational step, often together with other terms, broadly referred to as Darling–Dennison interaction terms.^82^ For this reason, the latter should be systematically considered for spectroscopic applications like those here. The general construction and use of polyads will be reviewed together with GVPT2.

#### 2.3.1 Fermi Resonances and DVPT2 Energies

At the VPT2 level, the energies in the polar representation can be systematically obtained through linear combinations of canonical energies and Darling–Dennison resonances. Let us first discuss the extension of the theoretical framework developed so far to the DVPT2 scheme, where each potentially resonant term in the **χ** matrix is analyzed by applying specific criteria, and those which are identified as resonant are discarded from the calculation. In the present work, the identification of Fermi resonances is done through a two-step procedure, which first considers the energetic proximity of the interacting states, namely, ω_*i*_ ≈ 2ω_*j*_ (type I) and ω_*i*_ ≈ ω_*j*_ + ω_*k*_ (type II), and then the magnitude of the term, using Martin’s test^80^ to estimate the deviation of the term from the variational energy of a model, ad hoc system. Currently, DVPT2 can be employed for both canonical and polar representations, the only difference lying in the definition of the **χ** matrix and the construction of the **g** matrix in the second case. However, it can be demonstrated that the formalism described in Section 2.2 can be easily extended to DPVT2 calculations since eq 7 is valid for both resonant and non-resonant terms separately
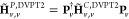
15

At this point, the transformation becomes similar to the VPT2 case presented above. An analogous procedure can be employed out to evaluate the **χ**^*P*^ and **g** matrices.

In practice, subtle differences could be observed because of the numerical parameters and tests used to define the resonances. However, for an equivalent set of resonances, the results obtained through eqs 6 and 15 (**χ**^*P*^ and **g** matrices being deprived of Fermi resonances) will converge.

#### 2.3.2 Variational Correction in GVPT2

GVPT2 is built on top of DVPT2 by adding a final step to calculate the anharmonic energies as eigenvalues of a variational matrix, whose diagonal elements are the DVPT2 energies, discussed in the previous section, and the off-diagonal elements represent the corrective terms to Fermi resonances, complemented by Darling–Dennison interactions, evaluated over the basis of the canonical harmonic-oscillator wave functions.

On the premise that our reference will remain the canonical representation, which also fully fits the conditions of the application of the GVPT2 scheme at first, we will discuss the theory underlying the definition of the polar variational matrix and then how a full equivalence can be reached between the two representations.

The calculation of a specific resonant term can be carried out by applying a simple generalization of eq 8 to the off-diagonal block coupling states differing in terms of principal quanta (***v*** ≠ ***v*′**), and it can be organized into two steps:

- Step 1: calculation of anharmonic energies and resonant terms in the canonical representation through the expressions reported in ref (78) (see Section S4 of the Supporting Information for more details);
- Step 2: combination of the canonical quantities evaluated in step 1 in order to obtain the polar resonant term of interest.

Through the symmetry relations between the anharmonic force constants of symmetric and linear tops, it is possible to prove that the corrective terms due to the Fermi resonances are equivalent in the two representations. Hence, the focus in the following will be on the couplings between states, collectively referred to as Darling–Dennison resonances or interactions.

In this context, particular importance is given to the resonances between states for which the condition ***v*** = ***v*′** holds, whose interaction generates the off-diagonal elements of the blocks **H̃**_**v,v**_^***P***^.

#### -Type Doubling in the Polar Representation

2.3.3

-type doubling terms involve overtones of a given degenerate mode or combination bands of two degenerate modes. Amat derived a general rule^24^ for the a priori identification of the non-vanishing off-diagonal terms
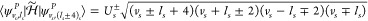
16
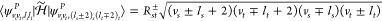
17
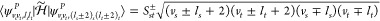
18where the elements defined in eq 16 contribute to the energy only if the order of the principal symmetry axis (*n* in *C*_*n*_) is a multiple of 4 and those in eq 18 only if *n* is even. The expressions of the terms *U*_*s*_^±^, *R*_*st*_^±^, and *S*_*st*_^±^ have been first derived by Grenier and Bresson^83,84^ and then re-derived in ref (71).

In the present derivation, there is no need of developing specific equations for computing the matrix elements defined in eqs 14–16 since they are off-diagonal elements of the diagonal blocks  and  (the full equations in terms of canonical quantities are reported in Section S5 of the Supporting Information). As a matter of fact, the calculation of -type terms can be performed concurrently with the conversion of the anharmonic energies through eq 7.

#### 2.3.4 Diagonalization and GVPT2 Energies

In the preceding sections, it has been shown that the blocks of the polar variational matrix (**H̃**^**P**^) can always be expressed in terms of their canonical counterpart. In order to understand the effects of such a connection on the GVPT2 energies, we will first consider a **H̃**^**P**^ matrix only containing the -type terms as off-diagonal elements. In this context, the variational problem simplifies to diagonalizing blocks of the type  and , whose eigenvalues are equal to those of their canonical counterparts. Therefore, the inclusion of -type doubling at the variational level implies the convergence of GVPT2 energies in the two representations.

This result can be easily generalized, given that an equivalent set of resonances is included in both representations, leading to the following identity:
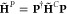
19where **P** is a block-diagonal matrix composed of all rotation matrices required for converting the different diagonal blocks **H̃_*v*,*v*_^*C*^**, and it is itself unitary.

In analogy with the treatment of -type doubling, the invariance of the eigenvalues of a matrix under unitary transformations can be exploited to state that canonical and polar GVPT2 energies converge to the same values. Let us remark that the -type terms are present even in the absence of accidental resonances. Consequently, their inclusion is mandatory in order to reach the convergence of the GVPT2 energies. From a practical point of view, eq 19 prevents any ambiguity connected to the representation choice since the set of GVPT2 energies is unique.

### 2.4 Transition Moments and Intensities

Starting from the available literature (e.g., the study of Tarrago and co-workers^85^ on the transition dipole moments of *C*_3*v*_-symmetry systems), a general computational framework to calculate both IR intensities and Raman activities of molecular systems with non-Abelian symmetries has been devised and implemented in our platform.

Let us start from the band intensities and the associated transition moments of linear and symmetric tops taking into account that thanks to the transformation shown in eq 5, it is possible to use the formulas obtained for asymmetric tops (and reported in Section S6 of the Supporting Information) to obtain their counterparts in the polar representation.

If we look at the fundamental bands (the full equation is reported in Sections S6.1.1 and S6.2.1 of the Supporting Information for degenerate modes), terms of the form
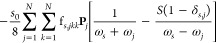
20where ***P***_*j*_ collects the Cartesian components of the first derivative of the property ***P*** with respect to the *j*th dimensionless normal coordinate, will present a singularity whenever *j* = *s*_2_. For this reason, it is necessary to exclude those modes in the summation so that the degenerate modes are assumed to be resonant, and the “resonant” form (Section S6.2.1 in the Supporting Information) is used instead. For simplicity, only transitions from the ground state (noted 0) to a given final state *f* are considered, noted “0;*f*”.

The quantities of interest here for IR and Raman spectroscopies are the dipole strength and Raman activity, labeled in the canonical representation *D*_0;*f*_^*C*^ and *S*_0;*f*_^*C*^, respectively,
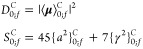
21Here, the invariants {*a*^2^}_0;*f*_^*C*^ (isotropic) and {γ^2^}_0;*f*_^*C*^ (anisotropic) for the most general case of a complex tensor are defined as^86,87^
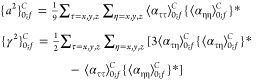
22where τ and η run over the Cartesian axes, while ⟨**μ**⟩_0;*f*_^*C*^ and ⟨α_τη_⟩_0;*f*_^*C*^ represent respectively the transition integrals of the electric dipole and a component of the polarizability tensor between the canonical states |ψ_0_^*C*^⟩ and |ψ_*f*_^*C*^⟩. Let us anticipate that a closure relation having the same form as eq 8 also holds for dipole strengths and Raman activities due to the unitarity of the rotation matrices.

The theoretical framework currently used for the calculation of transition moments can be straightforwardly extended to the polar representation (for more details, see Appendix B). Thus, once the transition dipole moments and polarizabilities are converted through eq 60, the calculation of both IR and Raman intensities in the polar representation is possible. It is worth mentioning that even though the transition moments evaluated in this way are generally complex, the corresponding intensities are always real.

#### 2.4.1 Infrared Intensities and Raman Activities at the VPT2 Level

For readability, the initial-state label will be dropped, so *D*_0;*f*_ will be simply written *D*_*f*_. In analogy with energies, the dipole strengths and Raman activities for fundamental states are the same if they are degenerate so that their contribution to the anharmonic spectra is independent of the representation. This result simplifies considerably the whole conversion procedure since among states with up to two quanta, the only states potentially different with respect to the canonical representation are the sets  and .

Concerning degenerate overtones, the dipole strengths of the polar states are
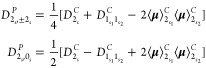
23where  and  are respectively the vectors containing the Cartesian components of the transition dipole moments associated with the states  and , and  is defined as
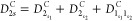
24

The Raman activities can be expressed in a compact notation through the introduction of the variables *A*_*r*;*s*_^*C*^, Γ_*r*;*s*_^*C*^, and *S*_*r*;*s*_^*C*^
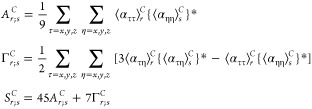
25which can be interpreted respectively as the “off-diagonal” terms of {*a*^2^}_*r*_^*C*^, {γ^2^}_*r*_^*C*^, and *S*_*r*_^*C*^.

As a consequence, the expressions of the Raman activities of the polar states of interest are
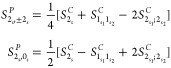
26where
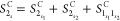
27

A similar analysis applied to the transition moments of binary combination bands involving degenerate modes yields the dipole strengths of the polar states
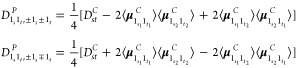
28where
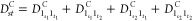
29

Finally, the corresponding Raman activities are
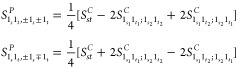
30where
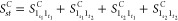
31

From a comparison of eqs 25 and 26 with eqs 11a and 11b and eqs 28 and 30 with eqs 13a and 13b, it is possible to observe that the conversion of dipole strengths and Raman activities is ruled by expressions similar to those employed for the anharmonic energies.

#### 2.4.2 Introduction of the Variational Correction

It has been demonstrated that if the variational matrix **H̃**^*P*^ in the polar representation can be expressed by a rotation of the canonical one **H̃**^*C*^ (see eq 19), the anharmonic energies within the two representations converge to the same values. This result can be easily extended to vibrational intensities. Indeed, while the definition of variational states is dependent on the representation of the reference states, polar and canonical variational states are equivalent when projected onto the same basis. In this context, the canonical basis is chosen as a reference.

As a matter of fact, the variational states, and hence the intensities, do not depend on the representation. Let us remark that the equality of the variational states holds even when only -type doubling terms are included as off-diagonal elements of the variational matrix, that is, even in the absence of accidental resonances.

In summary, the anharmonic spectrum is completely independent of the representation, with the only difference being the harmonic-state basis. Thus, the computational protocol currently employed for asymmetric tops can be straightforwardly extended to the treatment of symmetric/linear tops without any loss of accuracy.

### 2.5 Extension to Spherical Tops

In this section, we will show how the framework devised for symmetric and linear tops can be extended to spherical tops. For the sake of concision, the rotation-based formulation will be applied to systems presenting at most threefold degenerate vibrations. Actually, the eigenstates of the three-dimensional isotropic harmonic oscillator are again complex linear combinations of the canonical wave functions. This type of combinations can be extended to higher degeneracy orders, in which case, the following derivation can be straightforwardly adapted to systems exhibiting such characteristics.

Back to our application, the starting point remains the canonical representation since the specificity of the spherical top lies in the definition of the rotation matrices necessary for the transformation of the wave functions. While the canonical wave function is still given by eq 2, the so-called spherical wave function has the following form, where non-degenerate and doubly and triply degenerate modes are gathered in different terms
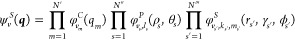
32where  are the solutions of the three-dimensional isotropic harmonic oscillator Hamiltonian expressed with respect to the spherical coordinates stemming from a trio of degenerate modes, *r*_*s*_, γ_*s*_, and ϕ_*s*_, and the quantum numbers *v*_*s*′_, *k*_*s*′_, and *m*_*s*′_ can assume the following values:
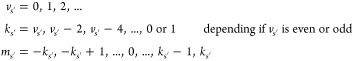
33

In analogy with symmetric and linear tops, the analysis of the spherical wave functions enables the definition of the rotation matrices **Q**_*v*_
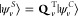
34where |ψ_*v*_^*S*^⟩ contains the spherical states sharing the same principal angular number ***v*** and |ψ_*v*_^*C*^⟩ is the corresponding canonical counterpart. Let us stress that when the states |ψ_*v*_^*S*^⟩ only involve non-degenerate and doubly degenerate modes, the rotation matrix is **P**_*v*_.

Let us now analyze the effect of rotations in deeper detail, highlighting the analogies with symmetric and linear tops. The canonical expression of ZPVE, whose expression is reported in eq 9, can be equivalently expressed through the following formula:
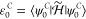
35where ψ_0_^*C*^ represents the harmonic ground-state wave function in the canonical representation. The main advantage of the rotation-based framework is that the specific properties of the rotor are collected in the definition of the wave function, more specifically through the use of rotation matrices to carry out the conversion procedure described above. As a result, the reference basis is always the canonical one, and the operators depending on the normal coordinates and their conjugate momenta are never subject to any modifications. Based on this, the expression of the spherical counterpart ε_0_^*S*^ is
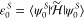
36where ψ_0_^*S*^ is the harmonic ground-state wave function in the spherical representation. Since the ground-state wave function is independent of the representation (see Appendix B for more details), we can conclude that
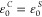
37

Hence, the resonance-free ZPVE in the spherical representation can be still evaluated through the customary expression, which can be then used for systems presenting both doubly and triply degenerate modes without any restriction.

In the previous sections, all different types of bands involving up to two-quanta excitations characterizing symmetric and linear tops have been derived and analyzed separately. In general terms, this separation is not necessary since only a few rotation matrices are actually sufficient to build all the other ones. With the aim of treating all the states up to two quanta, the largest value that the quantum numbers *v*_*m*_, *v*_*s*_, and *v*_*s*′_ reported in eq 34 can assume is 2 so that the matrices **P**_1_*s*__, **P**_2_*s*__, **Q**_1_*s*_,_ and **Q**_2_*s*__ allow to express any spherical state in terms of canonical ones (the extension to three-quanta states would only require additional matrices, **P**_3_*s*__ and **Q**_3_*s*__).

In the same way as symmetric and linear tops, both transition energies and intensities for states only involving one excited quantum of a threefold degenerate vibration are still independent of the representation, and this is also true for the ZPVE. Concerning overtones and binary combination bands involving triply degenerate modes, a complete derivation of both energies and intensities in the spherical representation for states up to two quanta is reported in Section S7 of the Supporting Information.

Finally, it is worth mentioning that the equivalence of representations at the GVPT2 level is not affected by the presence of triply degenerate vibrations, with it being strictly related to the connection of the wave functions by unitary matrices. Therefore, the GVPT2 results remain independent of the representation.

## 3 Computational Details

The theoretical framework presented in the previous sections has been implemented in a development version of the Gaussian package.^88^ Most of the available electronic structure computations allowing analytic computation of second energy and first property derivatives have been employed. These include HF and second-order Møller–Plesset (MP2) wave-function methods together with different flavors of DFT including representative hybrid (B3LYP^89−92^) and double-hybrid (B2PLYP^93,94^) exchange–correlation functionals, with the inclusion of empirical dispersion contributions by means of Grimme’s D3 model with Becke–Johnson damping^95,96^ (hereafter noted B3D3 and B2D3, respectively). As the core of this work concerns the development, functionals with a well-documented reliability on the molecular systems chosen here for illustration purposes were selected. The reliability of the B2PLYP functional in the calculation of both harmonic and anharmonic frequencies has been demonstrated in the literature.^97,98^ The B3LYP functional has been used for the calculation of both harmonic and anharmonic frequencies only for linear systems, where B2PLYP results have also been shown. Concerning symmetric and spherical tops, the B3LYP functional has been only used for the calculation of the anharmonic corrections in the hybrid scheme. In this respect, it has been demonstrated that the quality of the harmonic frequencies is much more critical if compared with that of the corresponding anharmonic corrections.^2^ The so-called calendar basis sets jun-cc-pVDZ and jun-cc-pVTZ^99^ have been consistently employed (referred to in the following as JnDZ and JnTZ, respectively).

The anharmonic data required for the VPT2 calculation of frequencies and intensities have been obtained by finite differences of analytical force constants (full cubic and semi-diagonal quartic force constants) and first-order derivatives of the properties (full second and semi-diagonal third derivatives), employing a default displacement of δ*Q*_*i*_ = 0.01 √amu·Å along each mass-weighted normal coordinate *Q*_*i*_.^42,100^ From a practical point of view, the generation of the anharmonic force field and higher-order property derivatives is the most expensive step once the equilibrium structure has been found. Indeed, 2*N* frequency calculations are needed in addition to the one at the reference geometry, required to generate the displacement vectors. Being independent from one another, they can be run in parallel on separate machines, with the final constants built at the end of the process. Hence, in an optimal scenario, the computational cost can be reduced to roughly twice what is needed at the harmonic level. The VPT2 calculations themselves on systems of this size last a few minutes, independent of the scheme chosen.

For the treatment of resonances, the protocol detailed in ref (76) was used with the default parameters.

In addition to the basic formulation of VPT2, the DVPT2 and GVPT2 schemes are also used for the calculation of both anharmonic energies and intensities. The use of DVPT2 is necessary in the presence of Fermi resonances to avoid the unphysical results issuing from the standard VPT2 equations. In addition to recovering the discarded terms from DVPT2, the GVPT2 scheme allows a straightforward account of Darling–Dennison resonances, which are not explicitly considered within the purely perturbative approach. Besides improving the overall agreement with experimental energies, they can be critical to obtain correct band shapes. However, the induced transformation can result in a significant deviation from the harmonic oscillator-based description of the vibrational states preserved by VPT2, thus requiring some extra work for the band assignment. Further details concerning the different VPT2 schemes concerning both vibrational energies and intensities have been recently reported in ref (101).

The so-called hybrid force field scheme has also been employed.^2,102−105^ In this approach, harmonic and anharmonic contributions are treated at different levels of theory in view of their different contribution to the final VPT2 result. In particular, anharmonic contributions have been consistently computed with the JnDZ basis set and harmonic terms with JnTZ. In some cases, which will be explicitly mentioned in the discussion, the results at the CCSD(T) level in conjunction with extended basis sets, already available in the literature, were employed for the latter. It should be noted that the higher-level harmonic frequencies are not simply added to anharmonic corrections but are also employed in the conversion of the anharmonic force constants and property derivatives, in the construction of the **χ** matrix, the definition of the resonant terms, and for the intensity. The JnTZ basis set has been employed for the calculation of anharmonic contributions of aromatic systems since it is well known that out-of-plane vibrations of these molecules are particularly sensitive to the basis-set dimension.^106−109^ An analogous remark applies to CO_2_.^110^

## 4 Results and Discussion

The framework previously discussed has been validated through a series of applications to linear and symmetric tops, illustrative of representative non-Abelian symmetry groups.

### 4.1 Linear Molecules

First, a set of three- and four-atom molecules, including hydrogen cyanide (HCN), hydrogen isocyanide (HNC), and acetylene (C_2_H_2_), has been considered. The absence of Fermi and 1–1 Darling–Dennison resonances (between fundamental states) implies that the anharmonic fundamentals do not vary going from VPT2 to DVPT2 or GVPT2 schemes. The fundamental harmonic and anharmonic frequencies obtained with the hybrid force-field model described above are reported in Table 1.

**Table 1:** Comparison of Experimental and Computed Anharmonic Fundamental Wavenumbers (in cm^–1^) for the Linear Molecules HCN, HNC, and C_2_H_2_^a^

		MP2//MP2^b^		B3D3//B3D3^b^		B2D3//B2D3^b^		
	symm.	ω	ν_VPT2_	ω	ν_VPT2_	ω	ν_VPT2_	exp.
HCN^c^								
|1_1_⟩	Σ	3459	3328	3440	3309	3455	3322	3312
|1_2_⟩		2023	1989	2199	2172	2125	2094	2097
|1_3_,±1_3_⟩	Π	710	704	757	738	740	726	714
MAE			45		34		8	
HNC^d^								
|1_1_⟩	Σ	3819	3658	3801	3632	3816	3650	3653
|1_2_⟩		2019	1986	2103	2070	2060	2025	2029
|1_3_,±1_3_⟩	Π	492	479	471	436	470	445	477
MAE			17		34		13	
C_2_H_2_^e^								
|1_1_⟩	Σ_*g*_	3525	3389	3512	3378	3524	3389	3372
|1_2_⟩		1969	1930	2068	2036	2024	1988	1975
|1_3_⟩	Σ_*u*_	3437	3312	3412	3287	3431	3305	3289
|1_4_,±1_4_⟩	Π_*g*_	592	561	663	621	638	602	613
|1_5_,±1_5_⟩	Π_*g*_	748	718	768	735	762	730	730
MAE			30		16		11	

a Mean absolute errors (MAEs) are also reported. The polar vibrational states are indicated as .

b Anharmonic calculations performed with the JnDZ basis set based on a set of harmonic frequencies evaluated through the JnTZ basis set.

c Experimental values from ref (69).

d Experimental values from ref (111).

e Experimental values from ref (112).

As can be seen from Table 1, the computed fundamental energies are in good agreement with the experimental counterparts, with the largest discrepancy concerning the degenerate mode of HNC. At the B3D3 and B2D3 levels of theory, such an error is most likely due to the underestimation of the corresponding harmonic frequency, which is lower of the experimental value in both cases.

As an example of comparison between the polar and canonical representations, the vibrational frequencies of CO_2_ for all states up to two quanta in the polar representation have been calculated and analyzed. The set of wavenumbers in the polar representation is reported in Table 2 and compared with reference experimental values.

**Table 2:** Comparison of Experimental and Computed Anharmonic VPT2, DVPT2, and GVPT2 Wavenumbers (in cm^–1^) of CO_2_ in the Polar Representation^a^

	MP2^b^				B3D3^b^				B2D3^b^				
state	ω	ν_VPT2_	ν_DVPT2_	ν_GVPT2_	ω	ν_VPT2_	ν_DVPT2_	ν_GVPT2_	ω	ν_VPT2_	ν_DVPT2_	ν_GVPT2_	exp.
Fundamentals													
|1_1_,±1_1_⟩	659	657	657	657	674	670	670	670	666	662	662	662	668^c^^,^^d^^,^^e^^,^^f^
|1_2_⟩	1326	1697	1309	1262	1369	1492	1349	1291	1341	1646	1321	1272	1285^c^^,^^d^^,^^e^^,^^f^^,^^g^
|1_3_⟩	2405	2367	2367	2367	2403	2356	2356	2356	2387	2342	2342	2342	2349^c^^,^^d^^,^^e^^,^^f^^,^^g^
Overtones													
|2_1_,±2_1_⟩	1319	1315	1315	1315	1347	1341	1341	1341	1332	1326	1326	1326	1336^d^^,^^e^^,^^f^
|2_1_,0_1_⟩	1319	933	1320	1368	1347	1203	1346	1403	1332	1005	1330	1379	1388^d^^,^^e^^,^^f^^,^^g^
|2_2_⟩	2652	3389	2614	2614	2739	2979	2692	2692	2682	3287	2635	2635	2548^g^
|2_3_⟩	4810	4714	4714	4714	4806	4688	4688	4688	4774	4660	4660	4660	4673^f^^,^^g^
Combinations													
|1_1_1_2_,±1_1_⟩	1986	2736	1960	1960	2043	2300	2013	2013	2007	2628	1978	1978	2077^d^^,^^f^
|1_1_1_3_,±1_1_⟩	3065	3013	3013	3013	3076	3014	3014	3014	3053	2992	2992	2992	3004^f^^,^^g^
|1_2_1_3_⟩	3731	4051	3664	3664	3772	3830	3686	3686	3728	3970	3644	3644	3613^d^^,^^e^^,^^f^^,^^g^
MAE		291	43	38		130	43	34		244	36	29	

a The polar vibrational states are indicated as .

b Basis set: JnTZ.

c Reference (113).

d Reference (114).

e Reference (115).

f Reference (116).

g Reference (117).

For comparison purposes, the VPT2, DVPT2, and GVPT2 wavenumbers in the canonical representation are reported in Table 3.

**Table 3:** Computed Anharmonic VPT2, DVPT2, and GVPT2 Wavenumbers (in cm^–1^) of CO_2_ in the Canonical Representation^a^

	MP2^b^				B3D3^b^				B2D3^b^			
state	ω	ν_VPT2_	ν_DVPT2_	ν_GVPT2_	ω	ν_VPT2_	ν_DVPT2_	ν_GVPT2_	ω	ν_VPT2_	ν_DVPT2_	ν_GVPT2_
Fundamentals												
|1_1*a*_⟩	659	657	657	657	674	670	670	670	666	662	662	662
|1_1*b*_⟩	659	657	657	657	674	670	670	670	666	662	662	662
|1_2_⟩	1326	1697	1309	1262	1369	1492	1349	1291	1341	1646	1321	1272
|1_3_⟩	2405	2367	2367	2367	2403	2356	2356	2356	2387	2342	2342	2342
Overtones												
|2_1*a*_⟩	1319	1124	1318	1315	1347	1272	1344	1341	1332	1166	1328	1326
|2_1*b*_⟩	1319	1124	1318	1315	1347	1272	1344	1341	1332	1166	1328	1326
|1_1*a*_1_1*b*_⟩	1319	1315	1315	1368	1347	1341	1341	1403	1332	1326	1326	1379
|2_2_⟩	2652	3389	2614	2614	2739	2979	2692	2692	2682	3287	2635	2635
|2_3_⟩	4810	4714	4714	4714	4806	4688	4688	4688	4774	4660	4660	4660
Combinations												
|1_1*a*_1_2_⟩	1986	2736	1960	1960	2043	2300	2013	2013	2007	2628	1978	1978
|1_1*b*_1_2_⟩	1986	2736	1960	1960	2043	2300	2013	2013	2007	2628	1978	1978
|1_1*a*_1_3_⟩	3065	3013	3013	3013	3076	3014	3014	3014	3053	2992	2992	2992
|1_1*b*_1_3_⟩	3065	3013	3013	3013	3076	3014	3014	3014	3053	2992	2992	2992
|1_2_1_3_⟩	3731	4051	3664	3664	3772	3830	3686	3686	3728	3970	3644	3644

a The canonical vibrational states are indicated as |*v*_*i*_*v*_*j*_⟩, and the subscripts “*a*” and “*b*” distinguish degenerate modes.

b Basis set: JnTZ.

As expected, the states involving at most one vibrational quantum in the degenerate bending mode have the same frequency irrespective of the chosen representation. Conversely, the frequencies of the first overtones related to the bending mode change between the representations, even though the energies converge to the same values when the GVPT2 model is applied.

By comparing Tables 2 and 3, it is straightforward to verify that the sum of the energies of the states  and  equals that of the states , , and . Such an outcome is in full agreement with eq 8.

Due to the strong Fermi resonance between the overtone of the bending mode and the fundamental of the symmetric stretching (2ω_1_ ≈ ω_2_), the CO_2_ molecule has been widely used as a prototype in the study of resonances. Let us underline that only the state  is involved in the resonance since the coupling between the states  and  is symmetry-forbidden. In order to show this from the mathematical point of view, let us consider the general case of a Fermi resonance between the fundamental of a non-degenerate mode *m* and the overtone of a degenerate mode *s*. Both possible resonant terms,  and , can be conveniently expressed in terms of canonical interaction terms
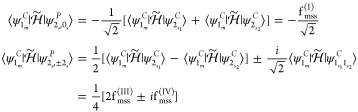
38where the expressions of the matrix elements associated to the Fermi resonances in the canonical representation^76^ have been used in conjunction with the symmetry rules and the definition of the force constants f_mss_^(σ)^ (σ = I, III, IV) reported in Table A2 of ref (71). For linear molecules, the only non-vanishing force constant f_mss_^(σ)^ corresponds to σ = I so that  always vanishes for this kind of systems.

**Figure 1:**
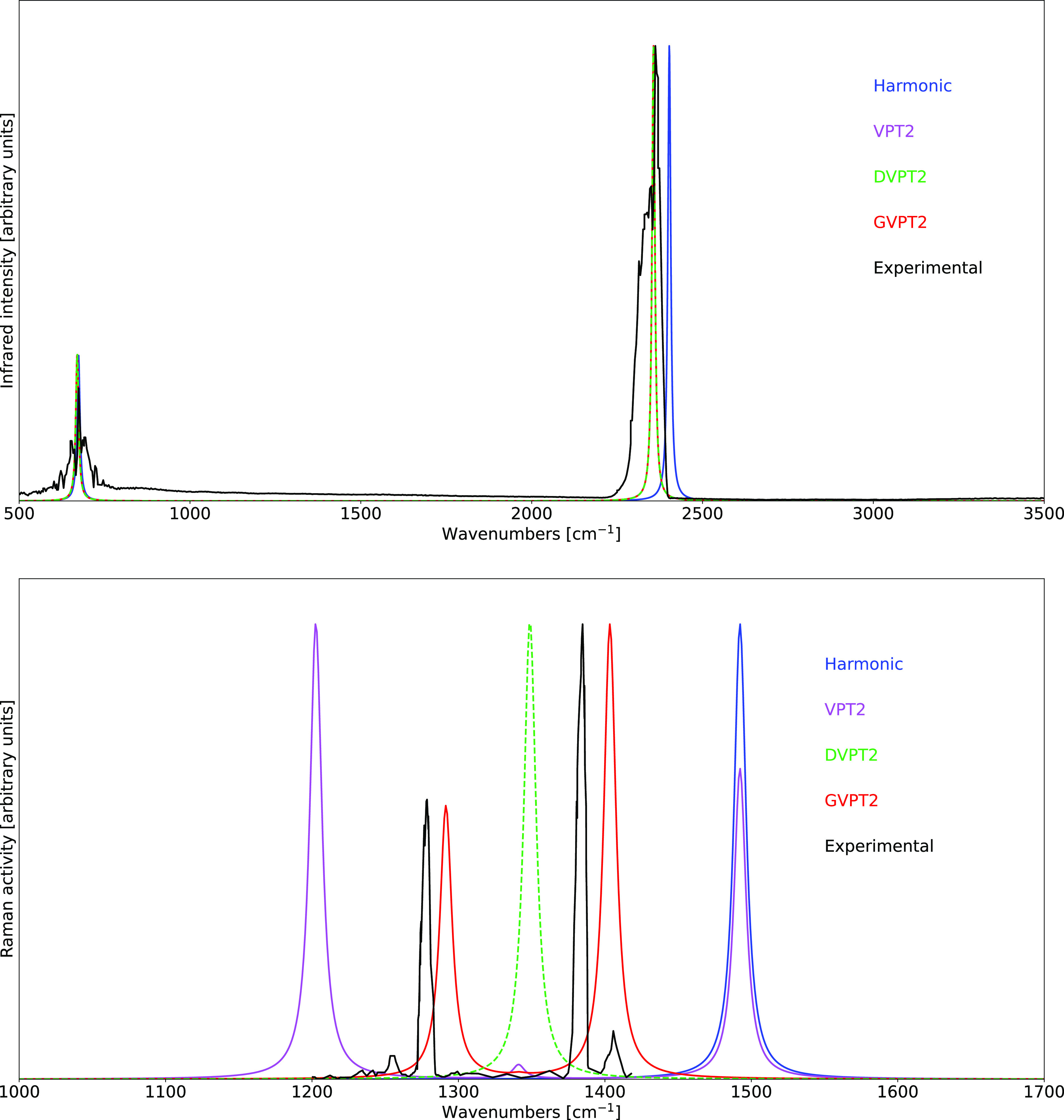
Comparison of the computed IR (top) and Raman (bottom) spectra of CO_2_ at the B3D3/JnTZ level of theory with the experimental data. Spectral line shapes have been convoluted by Lorentzian distribution functions with HWHMs of 5 cm^–1^. Experimental IR spectrum from the NIST Web Book.^118^ Experimental Raman spectrum from ref (119). All spectra are normalized by setting the intensity of their highest peak to unity.

The results obtained at the B3D3/JnTZ level of theory have been employed in the calculation of both IR and Raman spectra, with the harmonic and anharmonic results obtained within the VPT2, DVPT2, and GVPT2 schemes being compared with the experimental data in Figure 1. As expected, the best agreement between theoretical and experimental spectra is reached within the GVPT2 scheme. This is particularly evident in the Raman spectrum, in which the inclusion of the Fermi resonance discussed above at the variational level leads to an excellent reproduction of the relative intensities characterizing the Fermi diad present in the experimental spectrum.

**Figure 2:**
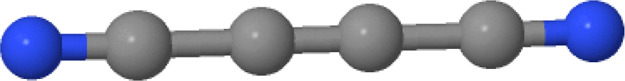
Molecular structure of dicyanoacetylene.

As a last example of linear molecules, dicyanoacetylene (C_4_N_2_, see Figure 2) is considered.

Dicyanoacetylene has been detected on the Titan moon of Saturn by IR spectroscopy, and it is used as a prototype for similar astrochemical systems, such as cyanopolyynes.^120^

The IR and Raman spectra of this system have been the object of several experimental works,^121−128^ while a theoretical analysis has been recently presented by Dargelos and Pouchan.^120^ With the aim of showing the application of our computational framework to longer chain systems, the VPT2, DVPT2, and GVPT2 fundamentals at different levels of theory have been calculated and compared with their experimental counterparts in Table 4.

**Table 4:** Comparison of Experimental and Computed Anharmonic Fundamental VPT2, DVPT2, and GVPT2 Wavenumbers (in cm^–1^) of Dicyanoacetylene^a^

		MP2//MP2^b^				B3D3//B3D3^b^				B2D3//B2D3^b^				
	symm.	ω	ν_VPT2_	ν_DVPT2_	ν_GVPT2_	ω	ν_VPT2_	ν_DVPT2_	ν_GVPT2_	ω	ν_VPT2_	ν_DVPT2_	ν_GVPT2_	exp.
|1_1_⟩	Σ_*g*_	2235	2174	2187	2178	2377	2607	2340	2315	2313	2236	2269	2254	2270
|1_2_⟩		2031	1991	1991	1991	2219	2190	2190	2190	2144	2110	2110	2110	2123
|1_3_⟩		600	606	606	606	617	634	611	617	610	623	604	610	606
|1_4_⟩	Σ_*u*_	2152	2105	2105	2105	2342	2309	2309	2309	2261	2221	2221	2221	2245
|1_5_⟩		1155	1149	1149	1149	1186	1186	1186	1186	1174	1171	1171	1171	1155
|1_6_,±1_6_⟩	Π_*g*_	504	422	422	422	559	330	330	330	537	375	375	375	505
|1_7_,±1_7_⟩		263	203	203	203	285	233	233	233	277	219	219	219	261
|1_8_,±1_8_⟩	Π_*u*_	478	463	463	463	510	475	475	475	497	469	469	469	472
|1_9_,±1_9_⟩		109	82	82	82	114	94	94	94	112	87	87	87	107
MAE^c^			58	57	58		71	35	33		21	15	17	

a Mean absolute errors (MAEs) are also reported. The polar vibrational states are indicated as .

b Anharmonic calculations performed with the JnDZ basis set based on a set of harmonic frequencies evaluated with the JnTZ basis set.

c States |1_6_,±1_6_⟩ excluded.

At both B3D3 and B2D3 levels, two Fermi resonances of the first type coupling the states |1_4_⟩ and |2_5_⟩, and |1_3_⟩ and |2_7_,0_7_⟩ are detected, with only the former being present at the MP2 level. Within the GVPT2 scheme, such Fermi resonances have been included variationally, together with the proper -type doubling terms, and 2–2 Darling–Dennison resonances, with the latter being present only at the B3D3 and B2D3 levels.

**Figure 3:**
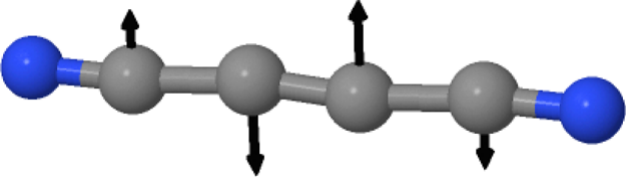
Graphical representation of normal mode 6 of dicyanoacetylene.

The best agreement between theoretical and experimental fundamentals is reached at the B2D3 level, despite an out-of-scale discrepancy detected for the degenerate states |1_6_,±1_6_⟩ regardless of the electronic level of theory. The corresponding normal mode is depicted in Figure 3.

At the MP2 level, such a difference can be ascribed to an underestimation of the harmonic frequency (which is lower than the experimental value), whereas density functional calculations show an excessive anharmonic correction (229 and 158 cm^–1^ for B3D3 and B2D3, respectively). Interestingly, the MP2 harmonic energies of the |1_6_,±1_6_⟩ fundamentals are very close to those of the experiment, a trend observed for all modes below 1000 cm^–1^.

### 4.2 Symmetric Tops

Shifting to symmetric-top systems, we analyze a set of six molecules, namely, bromotrifluoromethane (CF_3_Br), mono- and tri-deuterated methane (CH_3_D and CHD_3_, respectively), the cyclopentadienyl anion (C_5_H_5_^–^), benzene (C_6_H_6_), and pentaborane (B_5_H_9_), which are characterized by a principal axis of order ranging from 3 to 6 and belong to the molecular point groups *C*_*nv*_ and *D*_*nh*_. As already pointed out, anharmonic corrections at the MP2 level are free from the issues of numerical integration, which is not always true for methods rooted into DFT. In this respect, MP2 is more suitable for validation purposes of the new rotation-based framework, significantly reducing the possibility of errors in the symmetry relations proposed by Amat and Henry, and characterizing the anharmonic force field of symmetric and linear tops. As a matter of fact, while MP2 corrections will be used for all symmetric tops studied in the following, the use of DFT will be limited to the *C*_3*v*_ systems.

**Figure 4:**
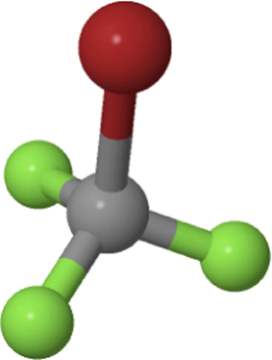
Molecular structure of CF_3_Br and CH_3_D.

Let us start from CF_3_Br (see Figure 4), whose anharmonic IR spectrum has been simulated at the MP2/JnDZ level of theory with harmonic frequencies corrected by CCSD(T)/aug-cc-pVTZ-PP^129^ calculations. In the former case, no Fermi resonances are present, while in the latter case, a single Fermi resonance of the second type, involving the non-degenerate fundamental |1_1_⟩ and combination |1_2_1_3_⟩, has been detected. In both cases, the only Darling–Dennison resonances detected correspond to the -type doubling of type *R* (which is the only one present when the principal axis order is 3).

The set of anharmonic fundamental frequencies, together with the energy of state |1_2_1_3_⟩ (the only two-quanta state involved in a Fermi resonance within the CC//MP2 scheme), is compared with the experimental data in Table 5 where, as expected, the improvement of the results due to the use of coupled-cluster (CC) harmonic frequencies leads to excellent agreement.

**Table 5:** Comparison of Experimental and Computed Anharmonic VPT2, DVPT2, and GVPT2 Wavenumbers (in cm^–1^) of CF_3_Br^a^

		MP2//MP2^b^				CC//MP2^c^				
state	symm.	ω	ν_VPT2_	ν_DVPT2_	ν_GVPT2_	ω^d^	ν_VPT2_	ν_DVPT2_	ν_GVPT2_	exp.^e^^,^^f^^,^^g^
|1_1_⟩	*A*_1_	1101	1080	1080	1080	1104	1082	1091	1086	1085
|1_2_⟩		770	764	764	764	763	757	757	757	761
|1_3_⟩		363	361	361	361	353	351	351	351	350
|1_4_,±1_4_⟩	*E*	1221	1196	1196	1196	1230	1206	1206	1206	1209
|1_5_,±1_5_⟩		553	547	547	547	548	542	542	542	550
|1_6_,±1_6_⟩		309	307	307	307	304	302	302	302	305
|1_2_1_3_⟩		1132	1132	1132	1132	1116	1119	1110	1115	1120
MAE			7	7	7		3	4	4	

a The polar vibrational states are indicated as .

b Anharmonic calculations performed with the JnDZ basis set based on a set of harmonic frequencies evaluated with the JnTZ basis set.

c Anharmonic calculations performed at the MP2/JnDZ level based on a set of harmonic frequencies evaluated at the CCSD(T)/aug-cc-pVTZ-PP level.

d Reference (130).

e Reference (131).

f Reference (132).

g Reference (133).

**Figure 5:**
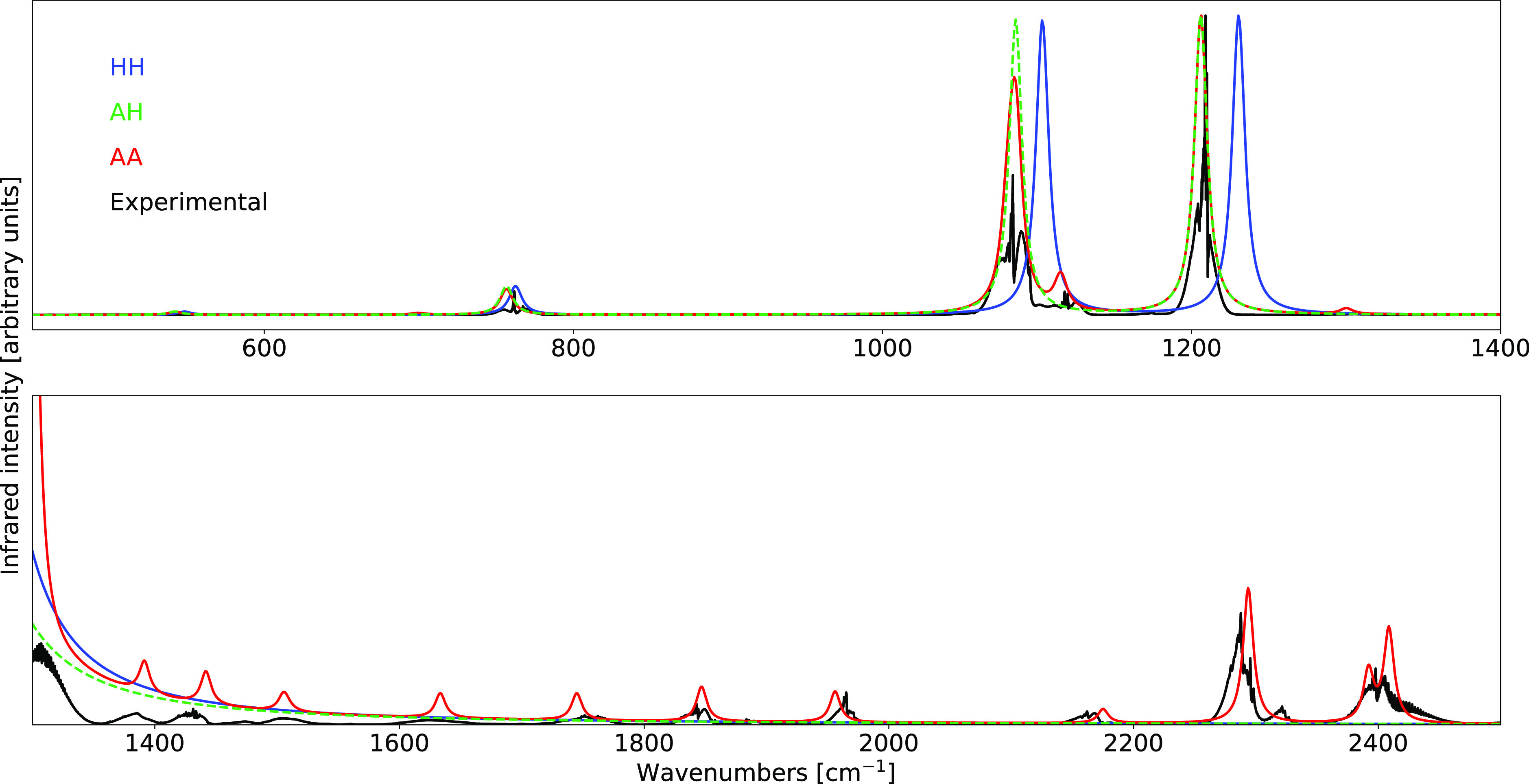
Comparison of the computed IR spectrum of CF_3_Br at the hybrid CC//MP2 level of theory with the experimental data. The labels HH and AA indicate respectively the full harmonic and anharmonic spectra, while the label AH indicates the inclusion of anharmonic effects only for the correction of the energies. The spectral range has been segmented to highlight the structure in the region above 1400 cm^–1^, with the panel below reporting the spectrum scaled by a factor 100 with respect to the above one. Spectral line shapes have been convoluted by Lorentzian distribution functions with HWHMs of 5 cm^–1^. Experimental IR spectrum from ref (133). All spectra are normalized by setting the intensity of their highest peak to unity.

The hybrid CC//MP2 results have also been used in the calculation of the anharmonic IR spectrum. Furthermore, in order to show the importance of anharmonic effects in the reproduction of both the position and intensity of the bands of the spectrum, the theoretical spectra have been evaluated by a stepwise inclusion of the anharmonic corrections. More specifically, the IR spectrum has been first evaluated at the purely harmonic level (HH), followed by the inclusion of the anharmonic corrections to the energies (AH) and finally to both energies and intensities (AA). A full comparison of theoretical and experimental spectra is reported in Figure 5.

As can be seen from the top panel of Figure 5, a remarkable improvement in the position of the bands is obtained by correcting the transition energies. Conversely, the bottom panel is characterized by a total absence of theoretical peaks unless the anharmonic contributions to the intensities are included. Indeed, in the spectral window corresponding to the bottom panel, only overtones and combination bands have been detected experimentally, while only fundamental bands have non-vanishing intensities within the harmonic oscillator model. The anharmonic corrections to the intensities allow not only to bypass this limit but also to produce a spectral profile in full agreement with the experimental one.

With the aim of showing the application of the new framework to the calculation of isotopomers belonging to different point groups, the CH_3_D and CHD_3_ molecules (symmetric tops) will be compared to the CH_2_D_2_ isotopomer (asymmetric top), whereas the spherical top isotopomers (CH_4_ and CD_4_) will be analyzed in a later section. In this context, four hybrid schemes have been used for computing the wavenumbers of the systems under consideration, namely, the MP2//MP2, B2D3//B3D3, CC//MP2, and CC//B3D3 models in conjunction with the JnDZ (B3D3 and MP2), JnTZ (B2D3), and cc-pVQZ (CC) basis sets.^134^ A full comparison of all sets of theoretical data with the corresponding experimental counterparts is reported in Table 6.

**Table 6:** Comparison of Experimental and Computed Anharmonic Fundamental VPT2, DVPT2, and GVPT2 Wavenumbers (in cm^–1^) of CH_2_D_2_, CH_3_D, and CHD_3_^a^

		MP2//MP2^b^				B2D3//B3D3^c^				CC//MP2^d^				CC//B3D3^e^				
	symm.	ω	ν_VPT2_	ν_DVPT2_	ν_GVPT2_	ω	ν_VPT2_	ν_DVPT2_	ν_GVPT2_	ω	ν_VPT2_	ν_DVPT2_	ν_GVPT2_	ω	ν_VPT2_	ν_DVPT2_	ν_GVPT2_	exp.^f^
CH_2_D_2_																		
|1_1_⟩	*A*_1_	3148	3017	3017	3017	*3112	2986	2973	2994	*3103	2964	2950	2972	*3103	2975	2961	2982	2975
|1_2_⟩		*2268	2249	2197	2230	*2245	4174	2178	2153	*2237	2446	2162	2133	*2237	2430	2169	2142	2203
|1_3_⟩		1483	1449	1449	1449	1476	1445	1445	1445	1471	1436	1436	1436	1471	1440	1440	1440	1435
|1_4_⟩		1060	1040	1040	1040	1058	1041	1041	1041	1053	1034	1034	1034	1053	1036	1036	1036	1033
|1_5_⟩	*A*_2_	1373	1346	1346	1346	1364	1339	1339	1339	1360	1332	1332	1332	1360	1335	1335	1335	1331
|1_6_⟩	*B*_1_	3208	3060	3060	3060	3163	3018	3018	3018	3157	2999	2999	2999	3157	3009	3009	3009	3012
|1_7_⟩		1122	1098	1098	1098	1122	1101	1101	1101	1116	1092	1092	1092	1116	1095	1095	1095	1091
|1_8_⟩	*B*_2_	*2376	2305	2287	2312	*2342	2321	2257	2241	*2337	2285	2243	2225	*2337	2290	2251	2233	2235
|1_9_⟩		1272	1243	1243	1243	1273	1248	1248	1248	1265	1237	1237	1237	1265	1240	1240	1240	1236
MAE^g^			26	24	27		19	10	10		10	6	4		10	6	4	
CH_3_D																		
|1_1_⟩	*A*_1_	3113	3004	2977	2931	3084	2984	2952	2995	3071	2962	2927	2978	3071	2971	2934	2983	2970
|1_2_⟩		2320	2220	2237	2226	2291	2201	2201	2201	2285	2183	2183	2183	2285	2192	2192	2192	2220
|1_3_⟩		1347	1314	1314	1314	1347	1318	1318	1318	1340	1307	1307	1307	1340	1310	1310	1310	1307
|1_4_,±1_4_⟩	*E*	3208	3064	3064	3064	3163	3022	3022	3024	3157	3003	3003	3005	3157	3013	3013	3015	3017
|1_5_,±1_5_⟩		1522	1488	1488	1488	1513	1480	1480	1480	1508	1473	1473	1473	1508	1475	1475	1475	1471
|1_6_,±1_6_⟩		1195	1169	1169	1169	1194	1170	1170	1170	1188	1162	1162	1162	1188	1164	1164	1164	1161
MAE			19	14	20		8	9	10		4	10	4		3	8	4	
CHD_3_																		
|1_1_⟩	*A*_1_	3179	3035	3035	3035	3138	2997	2997	2997	3131	2978	2978	2978	3131	2987	2987	2987	2992
|1_2_⟩		*2220	2170	2157	2171	*2201	2157	2143	2158	2191	2142	2108	2140	2191	2146	2132	2147	2143
|1_3_⟩		1031	1010	1010	1010	1031	1012	1012	1012	1025	1005	1005	1005	1025	1007	1007	1007	1003
|1_4_,±1_4_⟩	*E*	*2376	2320	2288	2277	*2342	2234	2259	2252	2337	2202	2246	2239	2337	2212	2253	2245	2251
|1_5_,±1_5_⟩		1332	1304	1304	1304	1326	1301	1301	1301	1321	1293	1293	1293	1321	1295	1295	1295	1291
|1_6_,±1_6_⟩		1062	1043	1043	1043	1060	1042	1042	1042	1056	1036	1036	1036	1056	1037	1037	1037	1036
MAE			23	18	16		8	6	5		11	4	5		8	3	3	

a Mean absolute errors (MAEs) are also reported. The polar vibrational states are indicated as . The frequencies impacted by resonances are indicated with a “*”.

b Anharmonic calculations performed with the JnDZ basis set based on a set of harmonic frequencies evaluated with the JnTZ basis set.

c Anharmonic calculations performed at the B3D3/JnDZ level based on a set of harmonic frequencies evaluated at the B2D3/JnTZ level.

d Anharmonic calculations performed at the MP2/JnDZ level based on a set of harmonic frequencies evaluated at the CCSD(T)/cc-pVQZ level.

e Anharmonic calculations performed at the B3D3/JnDZ level based on a set of harmonic frequencies evaluated at the CCSD(T)/cc-pVQZ level.

f Reference (135).

g State |1_2_⟩ excluded.

Concerning CH_2_D_2_, two Fermi resonances, namely, ω_2_ ≈ 2ω_7_ and ω_8_ ≈ ω_4_ + ω_9_, have been found at all levels of calculation, with an additional one (ω_1_ ≈ 2ω_3_) detected at the B2D3//B3D3 level. Conversely, 1–1 and 2–2 Darling–Dennison resonances have not been identified. The resonance analysis carried out for CH_3_D shows the presence of a Fermi resonance of type I (ω_1_ ≈ 2ω_5_) and a 1–1 Darling–Dennison resonance (ω_1_ ≈ ω_4_) at all the computational levels employed here. A second Fermi resonance of type I (ω_2_ ≈ 2ω_6_) is also present at the MP2//MP2 level. Furthermore, a series of 2–2 Darling–Dennison interactions have been detected (including -type terms). Finally, CHD_3_ is consistently characterized by two Fermi resonances (ω_2_ ≈ 2ω_3_ and ω_4_ ≈ ω_3_ + ω_5_), with the addition of ω_4_ ≈ 2ω_6_ at the CC//MP2 level. While 1–1 Darling–Dennison resonances have not been found, different 2–2 Darling–Dennison resonances have been included at the variational level. Let us remark that in agreement with the analysis performed by Lee and co-workers,^134^ the state |1_1_⟩ of CH_3_D is strongly coupled with |2_7_,0_7_⟩ (but not with |2_7_,±2_7_⟩ since the coupling element vanishes based on eq 38) so that at the GVPT2 level, the states with energies (see Table 6) 2931, 2995, 2978, and 2983 cm^–1^ for MP2//MP2, B2D3//B3D3, CC//MP2, and CC//B3D3, respectively, and those with energies 3017, 2911, 2890, and 2899 cm^–1^ are basically equal mixtures of |1_1_⟩ and |2_7_,0_7_⟩.

As expected, the results closer to those of the experiment are those based on CC harmonic frequencies, although the B2D3//B3D3 scheme leads to quite satisfactory results. Besides, the results presented here are in good agreement with those reported in ref (134), as confirmed by a comparison of the **χ**^*P*^ and **g** matrices, for which only the diagonal elements are reported in Table 7 for readability.

**Table 7:** Comparison of Extrapolated and Computed Anharmonic VPT2 and DVPT2 **χ**^*P*^ and **g** Diagonal Elements (in cm^–1^) of CH_2_D_2_, CH_3_D, and CHD_3_^a^

	MP2//MP2^b^		B2D3//B3D3^c^		CC//MP2^d^		CC//B3D3^e^			
	ν_VPT2_	ν_DVPT2_	ν_VPT2_	ν_DVPT2_	ν_VPT2_	ν_DVPT2_	ν_VPT2_	ν_DVPT2_	CC.^f^	exp.^g^
CH_2_D_2_										
χ_11_^*C*^	–28.1	–28.1	–27.3	–27.3	–30.7	–30.7	–27.9	–27.9	–27.3	–26.4
χ_22_^*C*^	–14.4	–14.4	–14.1	–14.1	–15.7	–15.7	–14.4	–14.4	–14.1	–13.5
χ_33_^*C*^	–7.5	–7.5	–7.2	–0.5	–8.6	–1.3	–7.3	–0.5	–6.7	–9.1
χ_44_^*C*^	–5.2	–5.2	–5.0	–5.0	–6.0	–6.0	–5.0	–5.0	–4.5	–7.1
χ_55_^*C*^	–2.0	–2.0	–1.9	–1.9	–2.2	–2.2	–2.0	–2.0	–2.2	–3.6
χ_66_^*C*^	–32.2	–32.2	–31.5	–31.5	–35.2	–35.2	–32.1	–32.1	–31.6	–32.7
χ_77_^*C*^	–28.2	–2.3	–999.2	–1.7	–144.7	–2.4	–132.3	–1.7	–1.9	8.9
χ_88_^*C*^	–18.8	–18.8	–18.5	–18.5	–20.5	–20.5	–18.9	–18.9	–18.6	–19.3
χ_99_^*C*^	–5.2	–5.2	–4.4	–4.4	–5.8	–5.8	–4.5	–4.5	–4.4	–13.4
MAE^h^	3.4	0.5	111.0	0.8	17.6	2.2	14.8	1.0		
MAE^i^	6.0	3.1	114.0	3.9	19.4	4.4	17.7	3.9		
CH_3_D										
χ_11_^*P*^	–17.8	–17.8	–17.2	–17.2	–19.3	–19.3	–17.6	–17.6	–17.2	–15.9
χ_22_^*P*^	–31.5	–31.5	–31.4	–31.4	–34.6	–34.6	–31.9	–31.9	–31.2	–31.0
χ_33_^*P*^	–7.5	–7.5	–6.9	–6.9	–8.2	–8.2	–7.0	–7.0	–6.8	–20.3
χ_44_^*P*^	–32.1	–32.1	–31.5	–31.5	–35.1	–35.1	–32.2	–32.2	–31.6	–32.6
χ_55_^*P*^	–8.4	–1.7	–9.4	–1.5	–10.5	–1.8	–9.8	–1.5	–1.9	–19.3
χ_66_^*P*^	1.5	–2.8	0.8	0.8	0.5	0.5	1.0	1.0	0.3	–6.1
*g*_44_	12.6	12.6	12.7	12.7	13.8	13.8	12.9	12.9	12.6	13.3
*g*_55_	6.9	0.2	7.8	–0.1	8.7	0.4	8.2	–0.1	0.4	15.9
*g*_66_	–1.0	3.3	–0.1	–0.1	0.2	0.2	–0.3	–0.3	0.7	3.3
MAE^h^	2.0	0.9	1.9	0.3	3.2	1.4	2.2	0.5		
MAE^i^	5.3	5.9	5.0	6.8	5.2	7.1	4.9	6.7		
CHD_3_										
χ_11_^*P*^	–60.4	–60.4	–59.7	–59.7	–66.3	–66.3	–60.6	–60.6	–59.2	–59.5
χ_22_^*P*^	–9.1	–9.1	–8.8	–8.8	–9.8	–9.8	–9.1	–9.1	–8.8	–8.1
χ_33_^*P*^	–7.4	–0.8	–7.5	–0.4	–8.5	–0.8	–7.6	–0.4	–7.2	–18.1
χ_44_^*P*^	–18.9	–18.9	–18.6	–18.6	–20.6	–20.6	–19.0	–19.0	–18.6	–19.3
χ_55_^*P*^	–5.0	–5.0	–4.4	–4.4	–5.7	–5.7	–4.5	–4.5	–4.6	–7.4
χ_66_^*P*^	–5.9	–5.9	–6.0	–6.0	–6.9	–2.2	–6.0	–6.0	–5.5	–12.4
*g*_44_	8.2	8.2	8.3	8.3	9.0	9.0	8.4	8.4	8.3	8.7
*g*_55_	5.7	5.7	5.5	5.5	6.2	6.2	5.5	5.5	5.4	6.7
*g*_66_	3.3	3.3	3.3	3.3	4.0	–0.7	3.4	3.4	3.3	7.7
MAE^h^	0.4	1.1	0.2	1.0	1.8	3.0	0.4	1.1		
MAE^i^	3.1	3.8	3.1	3.9	3.5	5.4	3.1	3.9		

a Mean absolute errors (MAEs) are also reported.

b Anharmonic calculations performed with the JnDZ basis set based on a set of harmonic frequencies evaluated with the JnTZ basis set.

c Anharmonic calculations performed at the B3D3/JnDZ level based on a set of harmonic frequencies evaluated at the B2D3/JnTZ level.

d Anharmonic calculations performed at the MP2/JnDZ level based on a set of harmonic frequencies evaluated at the CCSD(T)/cc-pVQZ level.

e Anharmonic calculations performed at the B3D3/JnDZ level based on a set of harmonic frequencies evaluated at the CCSD(T)/cc-pVQZ level.

f Theoretical values obtained from CCSD(T)/cc-pVTZ anharmonic force constants in conjunction with harmonic frequencies at the CCSD(T)/cc-pVQZ level (see Tables V and VI of ref (134) for more details) and reported with one decimal place.

g Values from ref (136) and reported with one decimal place.

h Computed with respect to the reference theoretical (CC) values.

i Computed with respect to the experimental data.

**Figure 6:**
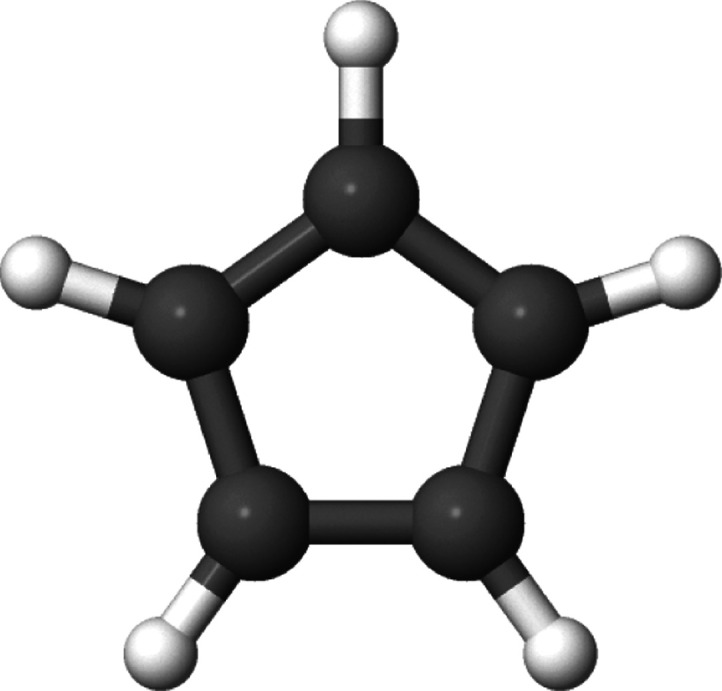
Molecular structure of the cyclopentadienyl anion.

**Figure 7:**
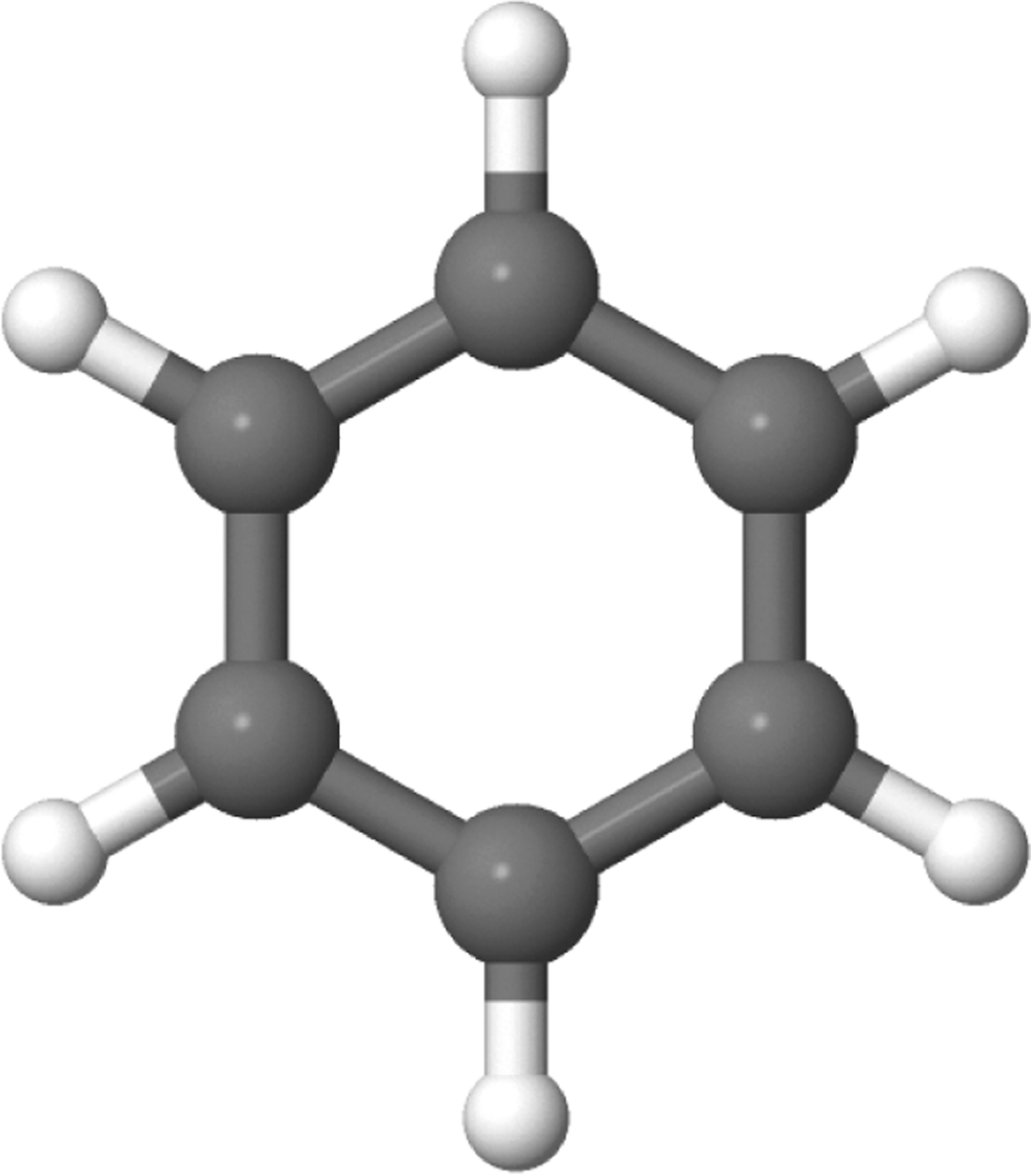
Molecular structure of benzene.

The next case studies are two planar aromatic systems, namely, the cyclopentadyenil anion and benzene (see Figures 6 and 7), with the former being noted Cp^–^ in the following.

As already anticipated, the presence of out-of-plane vibrations makes the use of a triple-ζ basis set mandatory for the calculation of the anharmonic force field so that the MP2/JnTZ level of theory has been employed for both systems. The assignment of the normal vibrations of the cyclopentadyenil anion has been recently revisited by Bencze and co-workers^137^ on the basis of previous studies.^138^ As outlined in ref (137), the cyclopentadienyl anion cannot exist without a counter cation under normal experimental conditions of IR and Raman spectroscopies. By means of a detailed analysis, the authors showed that the structural parameters of the Cp^–^ ring in the solid-state CpK should be close to those of the hypothetical free anion employed in the QC computations. Furthermore, the experimental frequencies of CpLi and CpNa in tetrahydrofuran (THF) were found to be very close to those of solid CpK. Experimental and theoretical anharmonic fundamental frequencies of Cp^–^ are reported in Table 8.

**Table 8:** Comparison of Experimental and Computed Anharmonic Fundamental VPT2, DVPT2, and GVPT2 Wavenumbers (in cm^–1^) of the Cyclopentadienyl Anion^a^

		MP2^b^				exp.		
state	symm.	ω	ν_VPT2_	ν_DVPT2_	ν_GVPT2_	CpLi^c^	CpNa^d^	CpK^e^
|1_1_⟩	*A*_1_^′^	3218	3087	3087	3087	3104	3090	3088
|1_2_⟩		1140	1118	1118	1118	1114	1114	1119
|1_3_⟩	*A*_2_^′^	1262	1237	1237	1237			1260
|1_4_,±1_4_⟩	*A*_2_^″^	663	652	652	652	710	722	686
|1_5_,±1_5_⟩	*E*_1_^′^	3196	3066	3066	3068	3082	3067	3061
|1_6_,±1_6_⟩		1448	1415	1415	1415	1433		1440
|1_7_,±1_7_⟩		1014	996	996	996	1006	998	1008
|1_8_,±1_8_⟩	*E*_2_^′^	3171	3043	3043	3042	3080	3060	3068
|1_9_,±1_9_⟩		1420	1612	1382	1375	1346	1342	1370
|1_10_,±1_10_⟩		1061	1043	1043	1043	1067	1062	1070
|1_11_,±1_11_⟩		830	819	819	819	854	848	854
|1_12_⟩	*E*_1_^″^	638	638	638	638	759	730	719
|1_13_,±1_13_⟩	*E*_2_^″^	780	787	787	787	735	722	686
|1_14_,±1_14_⟩		616	612	612	612			600
MAE^f^			40	23	22			

a Mean absolute errors (MAEs) are also reported. The polar vibrational states are indicated as .

b Basis set: JnTZ.

c Solution of CpLi dissolved in THF (ref (139)).

d Solution of CpNa dissolved in THF (ref (139)).

e Solid CpK (refs (137) and (139)).

f MAE computed with respect to the experimental values of CpK, excluding the states |1_13_,±1_13_⟩.

The results reported in Table 8 show that the VPT2 results are in good agreement with the experiment and that inclusion of the variational correction improves the agreement. The largest discrepancies concern the |1_12_⟩ and |1_13_,±1_13_⟩ states. There are, of course, systematic shifts related to the difference between the structure of the cyclopentadienyl anion in the experimental complexes and that of the free anion, with the lowest frequencies being, as usual, the most sensitive to environmental effects.

The next molecule studied is benzene (see Figure 7), a *D*_6*h*_ symmetric-top system, which has been extensively studied by both IR and Raman spectroscopies.^30,140−143^

The anharmonic calculations have been carried out at the MP2/JnTZ level or coupling the MP2/JnTZ anharmonic contributions to harmonic frequencies evaluated at the CCSD(T)/ANO4321′ level.^144^ The VPT2, DVPT2, and GVPT2 fundamentals of benzene computed at both levels of theory are compared with experimental data in Table 9.

**Table 9:** Comparison of Experimental and Computed Anharmonic Fundamental VPT2, DVPT2, and GVPT2 Wavenumbers (in cm^–1^) of Benzene^a^

		MP2^b^				CC//MP2^b^				
state	symm.	ω	ν_VPT2_	ν_DVPT2_	ν_GVPT2_	ω	ν_VPT2_	ν_DVPT2_	ν_GVPT2_	exp.^c^
|1_1_⟩	*A*_1*g*_	3235	3091	3107	3101	3210	3072	3072	3072	3074
|1_2_⟩		1011	997	997	997	1003	988	988	988	993
|1_3_⟩	*A*_2*g*_	1371	1338	1350	1346	1380	1348	1359	1355	(1350)
|1_4_⟩	*B*_2*g*_	996	1019	1019	1019	1009	1030	1030	1030	(990)
|1_5_⟩		710	709	709	709	709	708	708	708	(707)
|1_6_,±1_6_⟩	*E*_1*g*_	864	858	858	858	865	858	858	858	847
|1_7_,±1_7_⟩	*E*_2*g*_	3207	3084	3084	3084	3183	3061	3061	3061	3057
|1_8_,±1_8_⟩		1636	1601	1601	1601	1637	1600	1600	1600	1601
|1_9_,±1_9_⟩		1195	1180	1180	1180	1194	1179	1179	1179	1178
|1_10_,±1_10_⟩		606	603	603	603	611	609	609	609	608
|1_11_⟩	*A*_2*u*_	691	683	683	683	687	678	678	678	674
|1_12_⟩	*B*_1*u*_	3195	3106	3072	3026	3173	3117	3047	3047	(3057)
|1_13_⟩		1019	1014	1014	1014	1020	1015	1015	1015	(1010)
|1_14_⟩	*B*_2*u*_	1460	1418	1418	1418	1326	1288	1288	1288	1309
|1_15_⟩		1169	1156	1156	1156	1163	1149	1149	1149	1150
|1_16_,±1_17_⟩	*E*_1*u*_	3224	3111	3111	3111	3200	3089	3073	3045	3047
|1_17_,±1_17_⟩		1503	1476	1476	1476	1509	1481	1481	1481	1484
|1_18_,±1_18_⟩		1060	1040	1040	1040	1056	1035	1035	1035	1038
|1_19_,±1_19_⟩	*E*_2*u*_	977	978	978	978	985	985	985	985	976
|1_20_,±1_20_⟩		404	401	401	401	406	403	403	403	398
MAE			18	17	17		11	8	7	

a The polar vibrational states are indicated as .

b Basis set: JnTZ.

c Reference (30), the values in parentheses have not been observed directly but have been deduced from combination bands.

**Figure 8:**
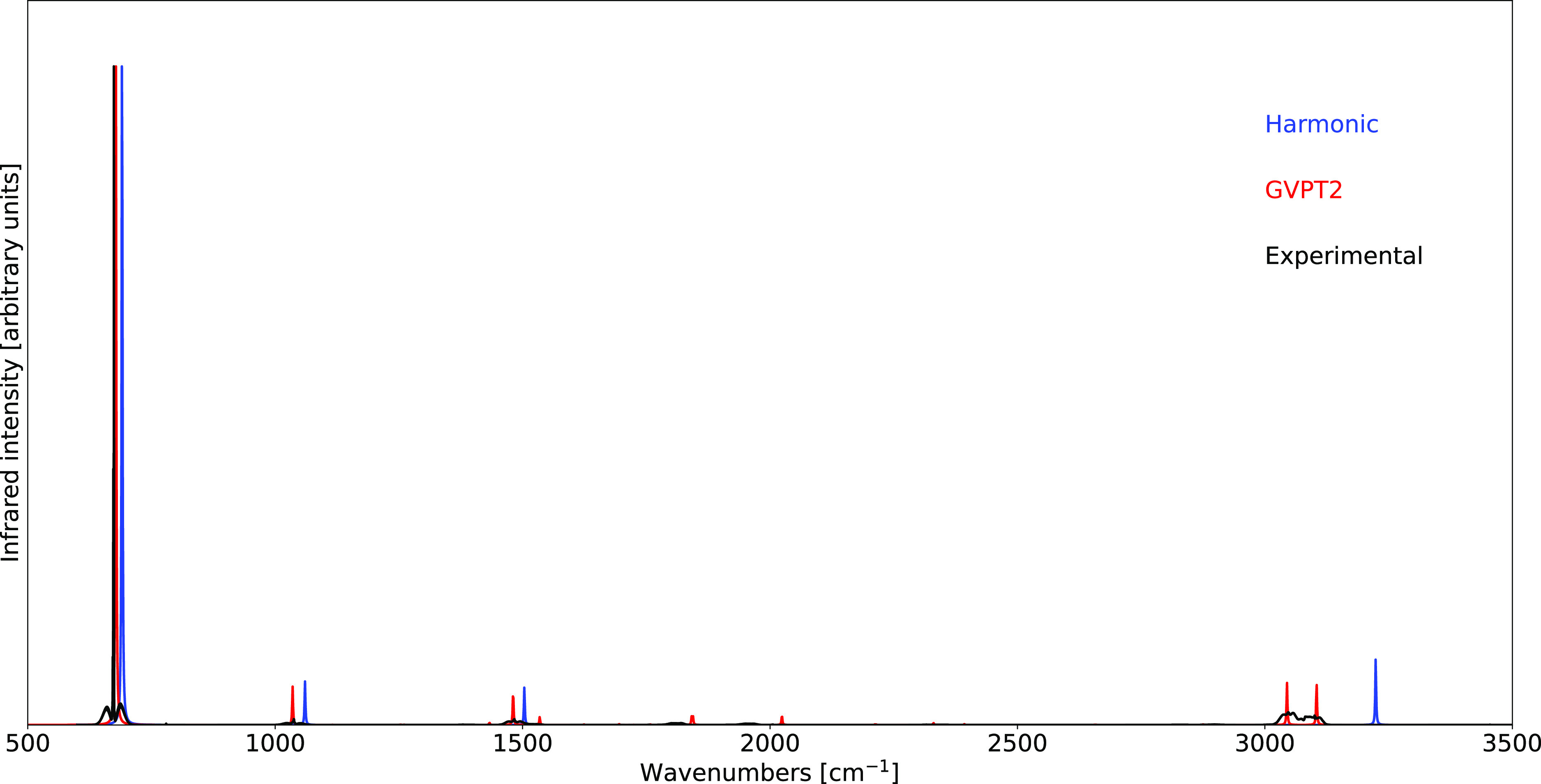
Comparison of the computed harmonic and anharmonic (GVPT2) IR spectra of benzene at the MP2/JnTZ level of theory with those of the experiment. Spectral line shapes have been convoluted by Lorentzian distribution functions with HWHMs of 1 cm^–1^. The experimental IR spectrum is from refs (145) and (146). All spectra are normalized by setting the intensity of their highest peak to unity.

Concerning the GVPT2 scheme, Fermi resonances of type II (ω_*i*_ ≈ ω_*j*_ + ω_*k*_) as well as 2–2 DD resonances, were found, with the latter including -type doubling of types *R* and *S*. On the other hand, 1–1 DD resonances have not been identified. The use of the hybrid scheme leads to a remarkable improvement of the fundamentals, and this is particularly true for the state |1_14_⟩, with the corresponding MP2/JnTZ harmonic frequency being clearly overestimated. On top of this, the inclusion of the variational correction further improves the agreement with the experimental data for all the transitions involved in resonances. The IR spectrum evaluated by means of the CC//MP2 hybrid scheme is compared with that of the experiment in Figure 8.

**Figure 9:**
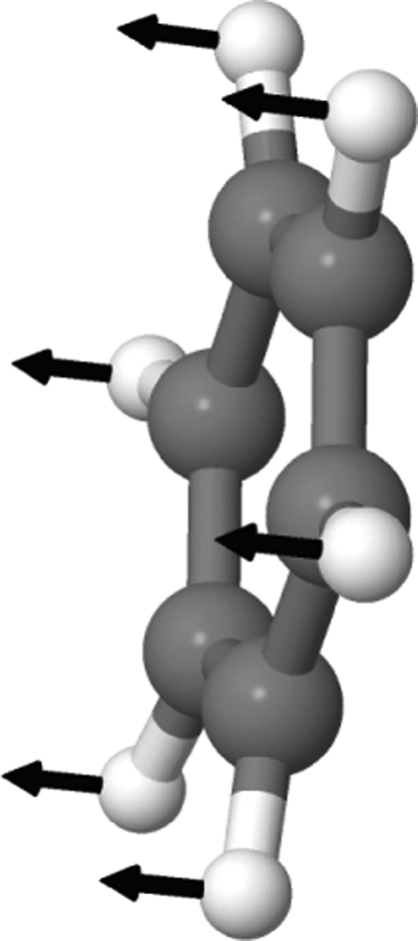
Vibration associated to the strongest band of the IR spectrum of benzene.

All spectra show a very strong band between 650 and 700 cm^–1^ corresponding to the fundamental transition to |1_12_⟩, associated to the out-of-plane bending vibration sketched in Figure 9.

Since such a state is not involved in any resonance, its position is independent of the adopted VPT2 scheme.

**Figure 10:**
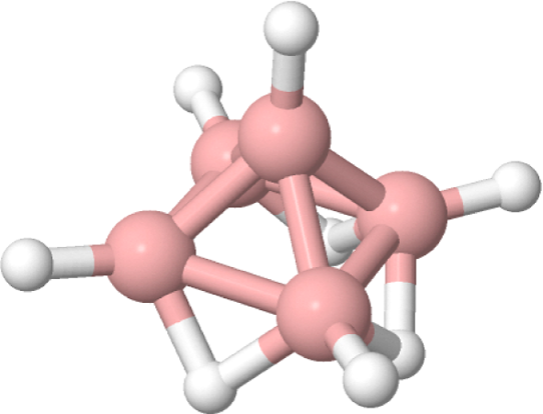
Molecular structure of pentaborane.

Finally, the simulation of the IR spectrum of pentaborane (see Figure 10), a *C*_4*v*_ system, has been carried out.

Among the systems considered in the present study, pentaborane is the only one showing all types of -type doubling within the GVPT2 scheme since the order of its principal axis is a multiple of 4. The simulation has been performed by employing the same hybrid scheme as for the other systems, namely, by coupling MP2/JnDZ anharmonic contributions to MP2/JnTZ harmonic frequencies. A full comparison of the theoretical and experimental fundamentals is reported in Table 10.

**Table 10:** Comparison of Experimental and Computed (VPT2, DVPT2, and GVPT2) Anharmonic Fundamental Wavenumbers (in cm^–1^) of Pentaborane

		MP2^a^				
state	symm.	ω	ν_VPT2_	ν_DVPT2_	ν_GVPT2_	Exp.
|1_1_⟩	*A*_1_	2773	2675	2675	2677	2628^b^
|1_2_⟩		2755	2657	2657	2651	2610^b^
|1_3_⟩		2026	1865	1852	1852	1844^b^
|1_4_⟩		1167	1125	1125	1125	1126^b^
|1_5_⟩		1011	981	981	981	985^c^
|1_6_⟩		825	801	801	801	799^b^
|1_7_⟩		725	709	709	709	701^b^
|1_8_⟩	*A*_2_	1523	1361	1373	1372	1450^c^
|1_9_⟩		883	849	849	849	
|1_10_⟩	*B*_1_	1987	1721	1839	1787	1870^d^
|1_11_⟩		1038	1010	1010	1010	1036^b^
|1_12_⟩		784	752	752	752	741^b^
|1_13_⟩		620	604	604	604	599^b^
|1_14_⟩	*B*_2_	2742	2640	2640	2639	2610^b^
|1_15_⟩		1718	1554	1568	1565	1500^c^
|1_16_⟩		812	792	792	792	785^b^
|1_17_⟩		729	706	706	705	
|1_18_⟩		491	473	473	473	470^d^
|1_19_,±1_19_⟩	*E*	2751	2649	2649	2649	2610^b^
|1_20_,±1_20_⟩		1988	1817	1851	1812	1634^b^
|1_21_,±1_21_⟩		1585	1467	1440	1436	1410^b^
|1_22_,±1_22_⟩		1086	1101	1047	1041	1035^b^
|1_23_,±1_23_⟩		958	927	927	927	918^b^
|1_24_,±1_24_⟩		907	886	886	886	890^b^
|1_26_,±1_26_⟩		649	624	624	624	618^b^
|1_27_,±1_27_⟩		595	576	576	576	569^b^
MAE			30	21	22	

a Mean absolute error (MAE) does not include state |1_20_,±1_20_⟩.

b Reference (147).

c Reference (148).

d Reference (149).

**Figure 11:**
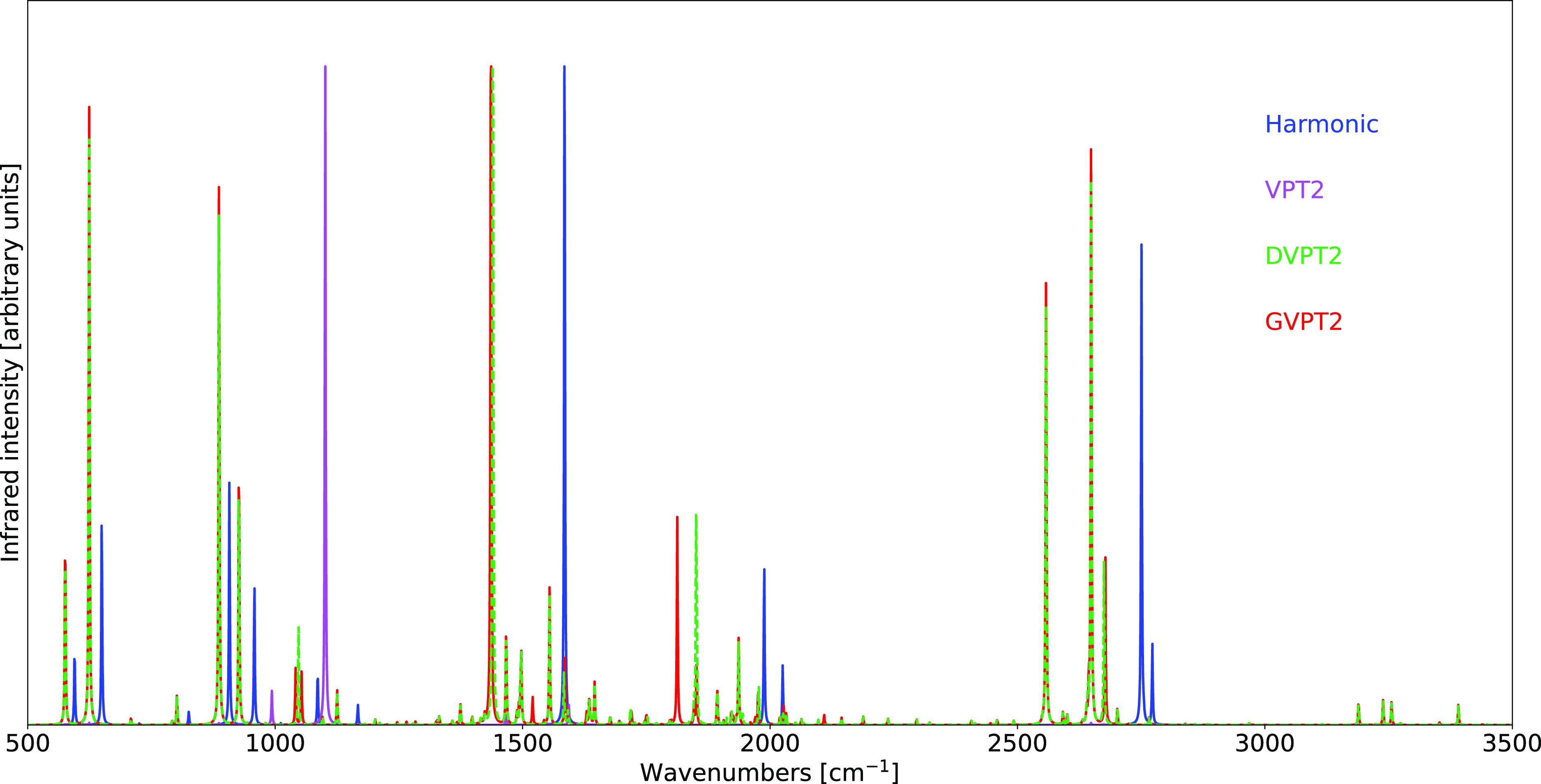
Comparison of the computed harmonic, VPT2, DVPT2, and GVPT2 IR spectra of pentaborane. Spectral line shapes have been convoluted by Lorentzian distribution functions with HWHMs of 1 cm^–1^. All the spectra are normalized by setting the intensity of their highest peak to unity.

At the DVPT2 level, several Fermi resonances (most of type II) have been detected, which have been successively included at the variational level within the GVPT2 scheme, together with the proper -type doubling terms and the other identified 2–2 Darling–Dennison resonances. Most of the frequencies are qualitatively correct and consistent with those reported in a recent study performed by Maillard and co-workers^150^ for several B–H systems (including, for instance, the out-of-scale discrepancy characterizing the states |1_20_,±1_20_⟩). For the sake of completeness, the IR spectrum of B_5_H_9_ is reported in Figure 11, where the theoretical data obtained by means of the VPT2, DVPT2, and GVPT2 schemes are compared.

While DVPT2 and GVPT2 spectra show a common pattern, the atypical behavior of the VPT2 spectrum is due to the strong Fermi resonances between the fundamentals |1_22_,±1_22_⟩ and the combination bands |1_18_1_27_,±1_27_⟩, which lead to a huge value for the transition dipole moment of the |1_22_,±1_22_⟩ states so that the corresponding band is the only one clearly visible in the theoretical spectrum. This problem is fixed within the DVPT2 scheme through the elimination of the resonant term, and the result is further refined at the GVPT2 level by the successive variational treatment.

### 4.3 Paving the Route to Spherical Tops

**Figure 12:**
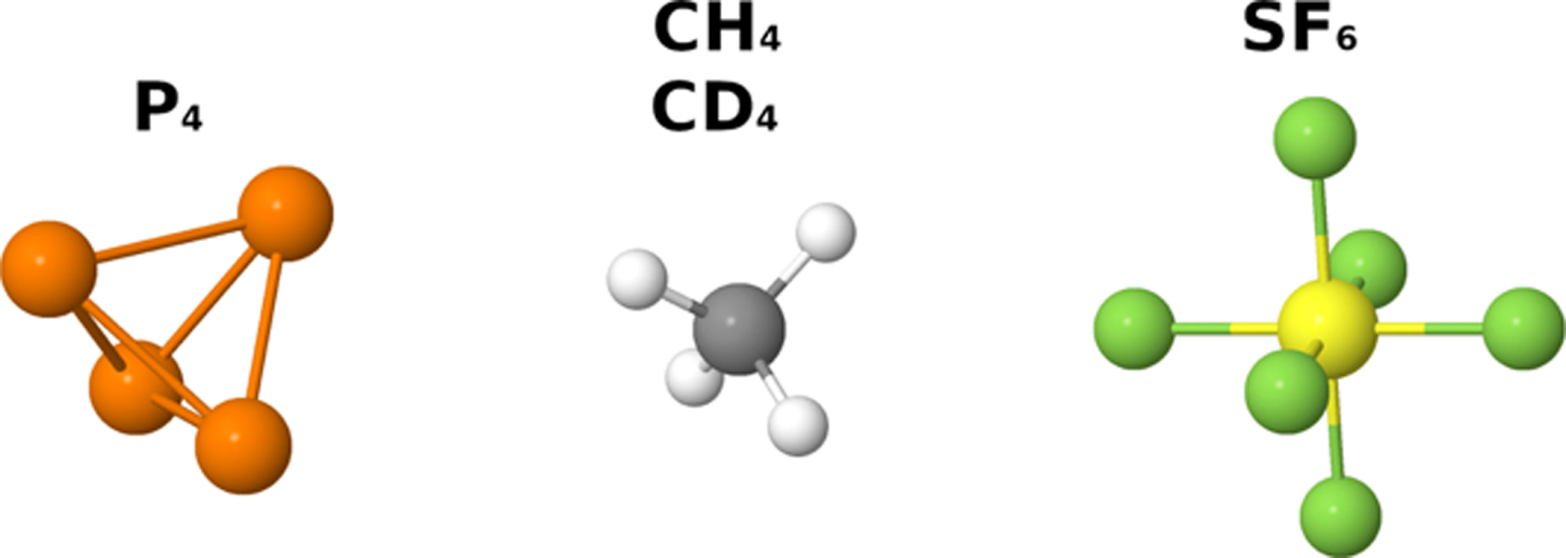
Molecular geometries of methane, tetraphosphorus, and sulfur hexafluoride.

With the aim of showing the extension of our computational framework to spherical tops, we now analyze a series of systems of both tetrahedral and octahedral symmetries, including, for instance, tetraphosphorus (P_4_), methane (CH_4_) and its fully deuterated isotopologue (CD_4_), and sulfur hexafluoride (SF_6_), whose structures are sketched in Figure 12.

In the case of linear and symmetric tops, our computational protocol performs by default a full check of all the symmetry relations present between the anharmonic force constants.^71^ Such a procedure has not yet been implemented for tetrahedral XY_4_-^136,151^ and octahedral XY_6_-like^152^ systems so that some slight (and generally negligible) discrepancies can be detected between anharmonic force constants which should be in principle identical. With the aim of limiting this issue as much as possible, the pruned (99,590) grid employed before for DFT calculations (which are in principle the most sensitive to this problem) will be replaced with a larger one (175,974 and 250,974 for first-row atoms and atoms in the second and later rows, respectively).

#### 4.3.1 Tetrahedral Molecules

The fundamental frequencies of the P_4_ molecule obtained through the MP2//MP2 and B2D3//B3D3 hybrid schemes are compared with their experimental counterparts in Table 11.

**Table 11:** Comparison of Experimental and Computed Anharmonic Fundamental VPT2 Wavenumbers (in cm^–1^) of Tetraphosphorus^a^

		MP2//MP2^b^		B2D3//B3D3^c^		
	symm.	ω	ν_VPT2_	ω	ν_VPT2_	exp.^d^
|1_1_⟩	*A*_1_	613	607	606	601	600
|1_2_,±1_2_⟩	*E*	365	363	363	361	361
|1_3_,1_3_,±1_3_ or 0_3_⟩	*T*_2_	463	459	458	454	467
MAE^c^			6		5	

a Mean absolute errors (MAEs) are also reported. The spherical and polar vibrational states are indicated respectively as |*v*_*i*_,*k*_*i*_,*m*_*i*_⟩ and .

b Anharmonic calculations performed with the JnDZ basis set based on a set of harmonic frequencies evaluated with the JnTZ basis set.

c Anharmonic calculations performed at the B3D3/JnDZ level based on a set of harmonic frequencies evaluated at the B2D3/JnTZ level.

d Reference (153).

The P_4_ system does not show any Fermi or 1–1 Darling–Dennison resonance so that the values of the fundamentals do not vary going from VPT2 to DVPT2 or GVPT2 schemes. As can be seen from Table 11, the frequencies of the states |1_1_⟩ and |1_2_,±1_2_⟩ are significantly improved within the B2D3//B3D3 scheme, reaching values within 1 cm^–1^ from the experimental counterparts. Concerning the triply degenerate states |1_1_,1_1_,±1 or 0_1_⟩, the best estimate is reached by the MP2//MP2 scheme, even though in both cases, the value of the frequency is not as accurate as the previous ones. The origin of such a discrepancy can be traced back to the experimental conditions at which the gas-phase spectrum has been recorded,^153^ as pointed out by Persson and co-workers^154^ in their very detailed vibrational analysis of P_4_. For the sake of completeness and as a consistency check of our calculations, the fundamentals calculated within the MP2//MP2 scheme have been compared with the MP2 results of ref (154), showing good agreement in terms of both harmonic frequencies and anharmonic corrections (see Table III of ref (154) for more details).

**Figure 13:**
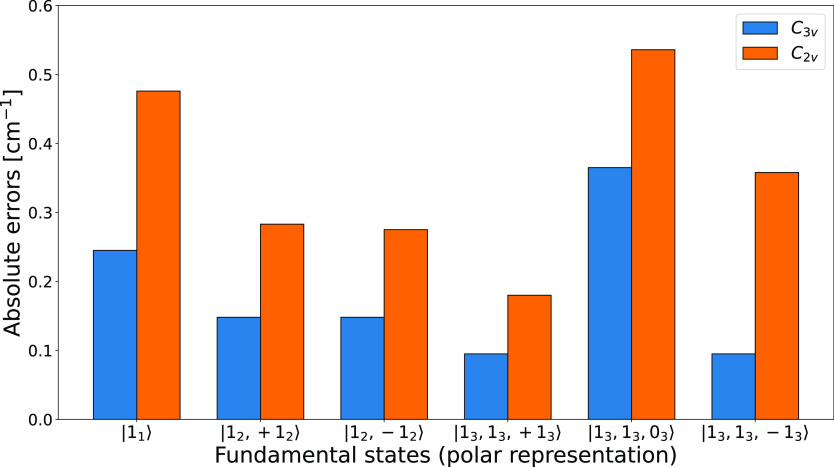
Errors between anharmonic fundamentals of P_4_ and the corresponding counterparts of the *C*_3*v*_ and *C*_2*v*_ symmetry-broken geometries obtained through the MP2//MP2 hybrid scheme.

A strategy often used to deal with systems presenting degeneracies is that of modifying slightly one or more masses/coordinates in order to lower the symmetry^134,140^ and then employ a theoretical model able to treat only lower- or non-degenerate modes. This procedure has been applied to P_4_ in order to perform a consistency check of our new implementation. More specifically, the *T*_*d*_ symmetry of this system has been gradually reduced without any geometry modification by slightly increasing (by 0.1%) the masses of a single and then a couple of phosphorous atoms, thus obtaining a symmetric (*C*_3*v*_ point group) or an asymmetric (*C*_2*v*_ point group) top, respectively. A comparison of the VPT2 fundamentals of the lower-symmetry systems with those of the fully symmetric molecule is reported in Figure 13.

As expected, triply degenerate states |1_3_,1_3_,±1_3_ or 0_3_⟩ are no more present, being replaced with a non-degenerate state of *A*_1_ symmetry and a couple of doubly degenerate states belonging to the irreducible representation *E* in the *C*_3*v*_ system and with three non-degenerate states with irreducible representations *A*_1_, *B*_1_, and *B*_2_ in the *C*_2*v*_ structure. This is clearly visible since the blue bars (*C*_3*v*_) corresponding to the states |1_3_,1_3_,±1_3_⟩ are equal to each other but different from that of |1_3_,1_3_,1_0_⟩, while the corresponding orange bars (*C*_2*v*_) are all different. The couple of states |1_2_,±1_2_⟩ belonging to the irreducible representation *E* are still present in the *C*_3*v*_ system, while they are replaced with two non-degenerate states with representations *A*_1_ and *A*_2_ in the *C*_2*v*_ system. Again, the blue bars of the states |1_2_,±1_2_⟩ are equal, while the corresponding orange ones are different, following the symmetry breaking.

As a further test, CH_4_ and CD_4_ are considered. In addition to the MP2//MP2 and B2D3//B3D3 schemes employed for P_4_, two hybrid calculations have been performed at the MP2/JnDZ and B3D3/JnDZ levels in conjunction with a set of harmonic frequencies at the CCSD(T)/cc-pVQZ level.^134^ The VPT2 wavenumbers are compared with the experimental data in Table 12.

**Table 12:** Comparison of Experimental and Computed Anharmonic Fundamental VPT2 Wavenumbers (in cm^–1^) of CH_4_ and CD_4_^a^

		MP2//MP2^b^		B2D3//B3D3^c^		CC//MP2^d^		CC//B3D3^e^		
	symm.	ω	ν_VPT2_	ω	ν_VPT2_	ω	ν_VPT2_	ω	ν_VPT2_	exp.
CH_4_^f^										
|1_1_⟩	*A*_1_	3073	2945	3051	2928	3036	2902	3036	2911	2921
|1_2_,±1_2_⟩	*E*	1586	1551	1575	1543	1570	1534	1570	1538	1532
|1_3_,1_3_,±1_3_ or 0_3_⟩	*T*_2_	3209	3067	3163	3027	3157	3007	3157	3017	3022
|1_4_,1_4_,±1_4_ or 0_4_⟩		1352	1319	1353	1323	1345	1311	1345	1316	1308
MAE			28		10		10		7	
CD_4_^f^										
|1_1_⟩	*A*_1_	2173	2124	2158	2113	2148	2098	2148	2102	2124
|1_2_,±1_2_⟩	*E*	1122	1103	1114	1097	1111	1092	1111	1094	1093
|1_3_,1_3_,±1_3_ or 0_3_⟩	*T*_2_	2376	2295	2342	2264	2337	2252	2337	2258	2260
|1_4_,1_4_,±1_4_ or 0_4_⟩		1022	1003	1023	1006	1017	998	1017	1000	1001
MAE			17		6		10		7	

a Mean absolute errors (MAEs) are also reported. The spherical and polar vibrational states are indicated respectively as |*v*_*i*_,*k*_*i*_,*m*_*i*_⟩ and .

b Anharmonic calculations performed with the JnDZ basis set based on a set of harmonic frequencies evaluated with the JnTZ basis set.

c Anharmonic calculations performed at the B3D3/JnDZ level based on a set of harmonic frequencies evaluated at the B2D3/JnTZ level.

d Anharmonic calculations performed at the MP2/JnDZ level based on a set of harmonic frequencies evaluated at the CCSD(T)/cc-pVQZ level.

e Anharmonic calculations performed at the B3D3/JnDZ level based on a set of harmonic frequencies evaluated at the CCSD(T)/cc-pVQZ level.

f Reference (135).

Both systems do not show any Fermi or 1–1 Darling–Dennison resonance, except for CD_4_ within the CC//MP2 hybrid scheme, where a Fermi resonance of type I (ω_1_ ≈ 2ω_4_) has been detected, leading the vibrational frequency of |1_1_⟩ shifts from 2064 to 2099 cm^–1^ between the DVPT2 and GVPT2 levels. The presence of 2–2 Darling–Dennison resonances has been detected in the GVPT2 model, and the resonances between states possessing the same principal quanta have been properly included. Concerning CH_4_, the best agreement between theory and experiments is reached with the CC//B3D3 hybrid scheme (MAE = 7 cm^–1^), even if all sets of VPT2 theoretical wavenumbers are close to those of the experiment, with the only exception being the MP2//MP2 scheme (MAE = 28 cm^–1^), for which larger discrepancies are observed.

The results for CD_4_ present a trend similar to that of CH_4_, with the MP2//MP2 scheme showing again the worst agreement with the experiment, although it is able to match exactly the experimental value of the fundamental band associated to the state |1_1_⟩. The hybrid schemes based on CC harmonic frequencies (particularly CC//B3D3) are quite close to the experimental data, even though in this case, the best agreement is reached by the B2D3//B3D3 scheme (MAE = 6 cm^–1^).

**Figure 14:**
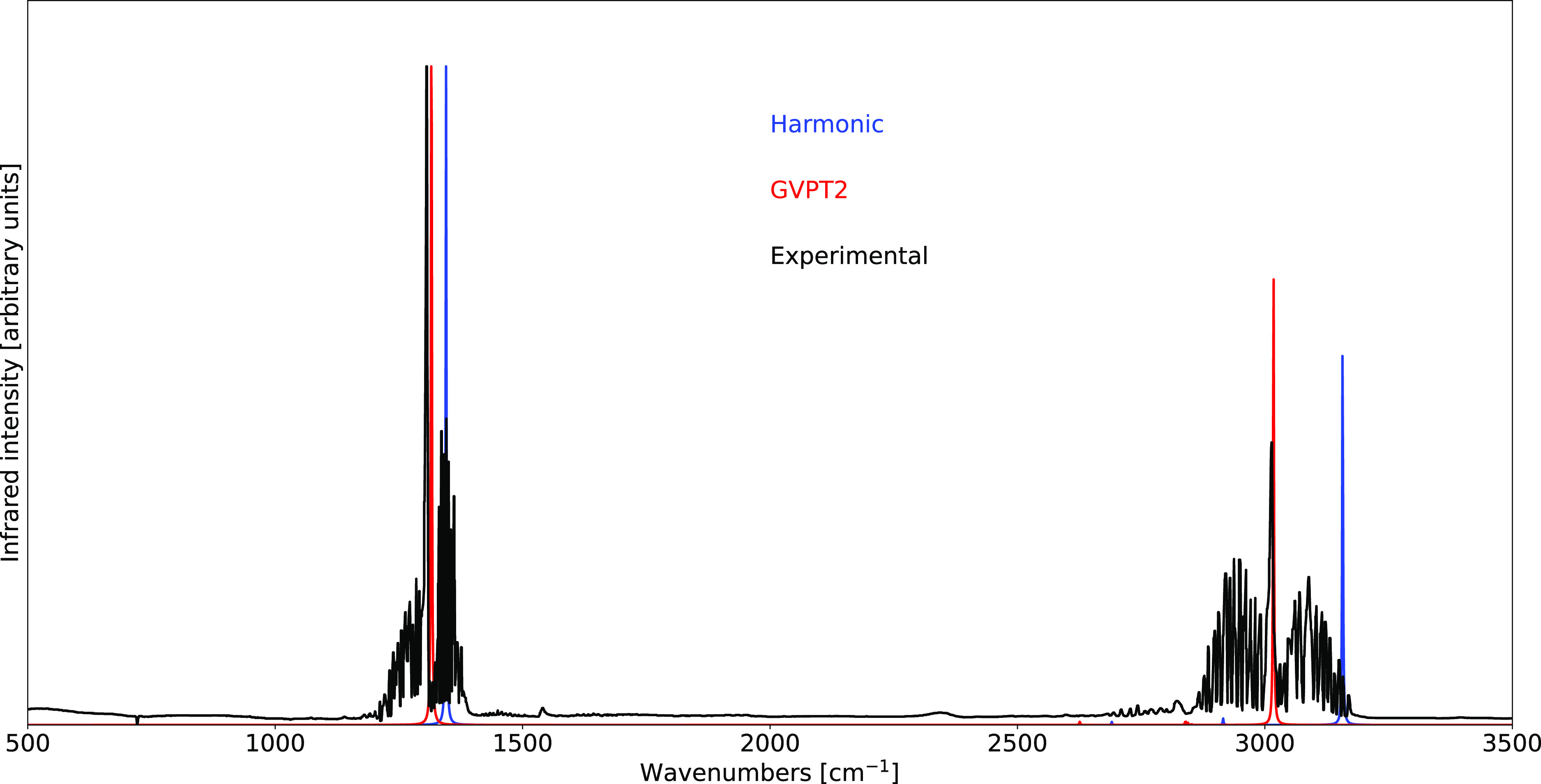
Comparison of the computed harmonic and GVPT2 IR spectra of methane at the CC//B3D3 level of theory with the experimental data. Spectral line shapes have been convoluted by Lorentzian distribution functions with HWHMs of 1 cm^–1^. The experimental IR spectrum is from ref (145). All spectra are normalized by setting the intensity of their highest peak to unity.

The anharmonic frequencies and intensities at the CC//B3D3 level have been employed in the simulation of the IR spectrum, which is compared with the harmonic and experimental ones in Figure 14.

As expected, the inclusion of anharmonic effects improves the agreement between theory and experiments, especially for the band around 3000 cm^–1^, corresponding to the excitation of the triply degenerate fundamental |1_3_,1_3_,±1_3_ or 0_3_⟩.

#### 4.3.2 Octahedral molecules

Finally, sulfur hexafluoride has been chosen as a test case for the *O*_*h*_ point group. The anharmonic calculations have been performed at the MP2/JnDZ and B3D3/JnDZ levels of theory, in conjunction with harmonic frequencies at the MP2/JnTZ and B2D3/JnTZ levels, respectively. A comparison between the VPT2 fundamental wavenumbers and their experimental counterparts is reported in Table 13.

**Table 13:** Comparison of Experimental and Computed Anharmonic Fundamental VPT2 Wavenumbers (in cm^–1^) of Sulfur Hexafluoride^a^

		MP2//MP2^b^		B2D3//B3D3^c^		
	symm.	ω	ν_VPT2_	ω	ν_VPT2_	exp.^d^
|1_1_⟩	*A*_1*g*_	771	762	748	740	775
|1_2_,±1_2_⟩	*E*_*g*_	646	638	629	622	643
|1_3_,1_3_,±1_3_ or 0_3_⟩	*T*_1*u*_	953	937	928	915	948
|1_4_,1_4_,±1_4_ or 0_4_⟩		606	600	592	586	615
|1_5_,1_5_,±1_5_ or 0_5_⟩	*T*_2*g*_	515	511	502	498	524
|1_6_,1_6_,±1_6_ or 0_6_⟩	*T*_2*u*_	342	339	334	330	348
MAE			11		27	

a Mean absolute errors (MAEs) are also reported. The spherical and polar vibrational states are indicated respectively as |*v*_*i*_,*k*_*i*_,*m*_*i*_⟩ and .

b Anharmonic calculations performed with the JnDZ basis set based on a set of harmonic frequencies evaluated with the JnTZ basis set.

c Anharmonic calculations performed at the B3D3/JnDZ level based on a set of harmonic frequencies evaluated at the B2D3/JnTZ level.

d Reference (155).

**Figure 15:**
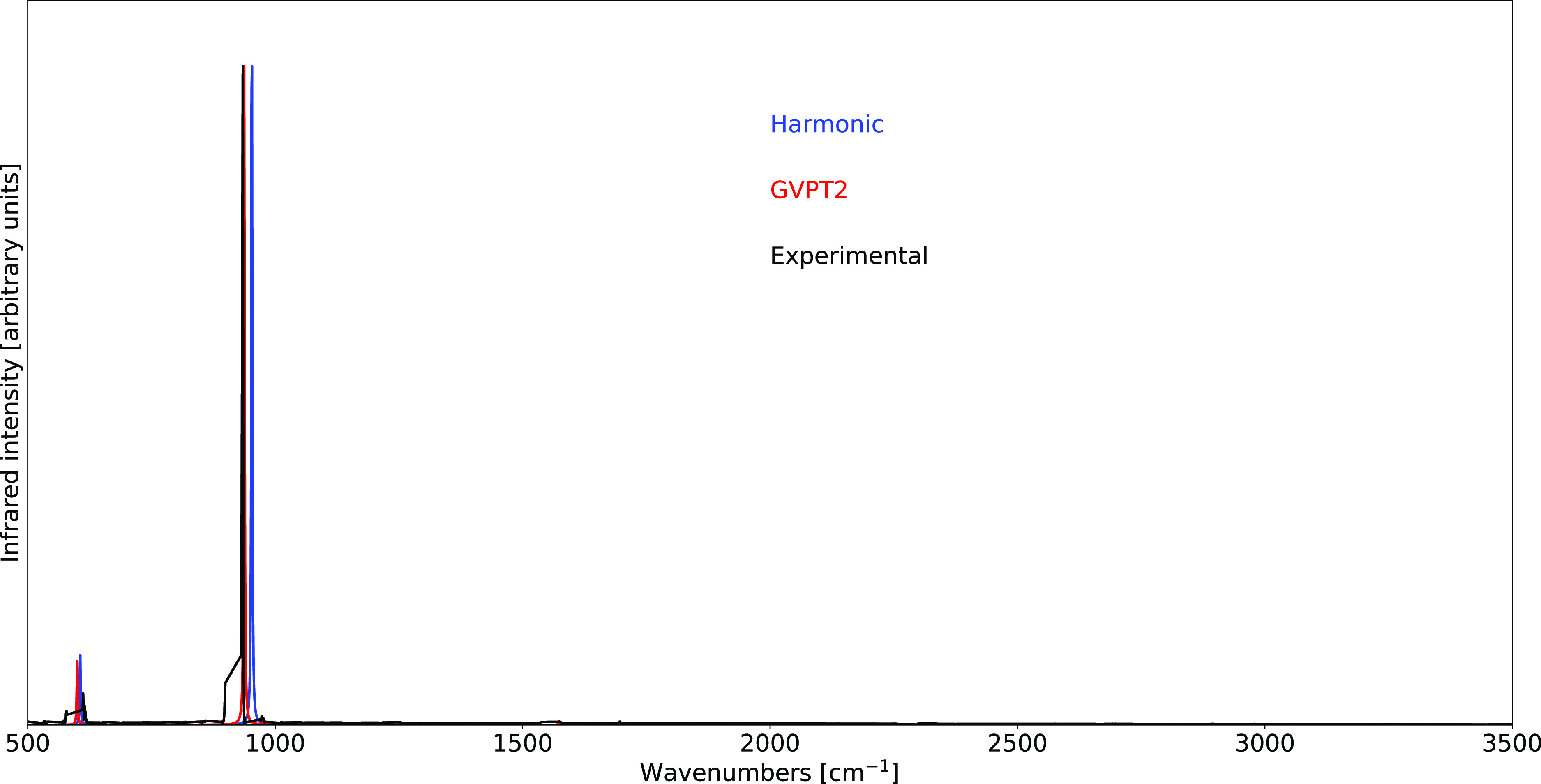
Comparison of the computed harmonic and GVPT2 IR spectra of sulfur hexafluoride obtained through the MP2//MP2 hybrid scheme of theory with the experimental data. Spectral line shapes have been convoluted by Lorentzian distribution functions with HWHMs of 1 cm^–1^. The experimental IR spectrum is from ref (145). All spectra are normalized by setting the intensity of their highest peak to unity.

This system does not show any Fermi or 1–1 Darling–Dennison resonance at the levels of theory employed here, while 2–2 Darling–Dennison resonances have been identified and included at the GVPT2 level. The best agreement with the experimental fundamentals is obtained at the MP2//MP2 level (MAE = 11 cm^–1^), while the B2D3//B3D3 scheme is in this case characterized by a significant underestimation of all harmonic wavenumbers. For this reason, the anharmonic results obtained at the MP2//MP2 level have been employed in the simulation of the IR spectrum. Both theoretical harmonic and GVPT2 spectra are compared with the experimental one in Figure 15.

Similar to CH_4_, the inclusion of anharmonic contributions leads to a spectral profile closer to the experimental one, especially for the position of the band around 1000 cm^–1^, corresponding to the triply degenerate fundamentals |1_3_,1_3_,±1_3_ or 0_3_⟩.

## 5 Conclusions

In this work, we have shown how the canonical representation used for the development of VPT2 equations of asymmetric tops can be extended to linear and symmetric tops, followed by a series of a posteriori transformations, to give results identical to those obtained with the polar representation, thus offering the possibility of an ease of choice of the most convenient form for any application in vibro-rotational and vibrational spectroscopies. Such a strategy offers a number of advantages with respect to previous, ad hoc procedures. The first aspect concerns the ease of implementation since the new approach does not require any heavy modification of the codes already supporting VPT2 for asymmetric tops. The second aspect is the simplicity of the extension to spherical tops. Once the transformation matrix between the representations is known, it is possible to derive the necessary equations for any quantity of interest, which can be coded in small, specialized routines. However, the most important advantage is the availability of general equations for the intensities of all vibrational spectroscopies without the need of resorting to complex numbers. Actually, to the best of our knowledge, this is the first completely general implementation of intensities in the framework of the double-perturbation theory.

The results show that we dispose now of a general and robust implementation of GVPT2 for Abelian and non-Abelian groups allowing the effective treatment of medium- to large-sized molecules for all electronic structure methods for which analytical Hessians and first derivatives of properties are available. Hybrid methods in which harmonic and anharmonic contributions are treated at different levels can further extend the range of applications of the general platform. Studies in condensed phases can also be performed by means of mixed discrete-continuum models in which the solute and, possibly, some molecules of its cybotactic region are embedded in a polarizable continuum mimicking bulk solvent effects. Also in this case, the availability of analytical Hessians and first derivatives of properties allows an effective GVPT2 treatment.

Of course, all the intrinsic problems of a low-order perturbative treatment based on Cartesian normal modes are still present, especially concerning large-amplitude motions. Besides, the harmonic-oscillator wave functions do not always provide a suitable basis for the representation of vibrations, regardless of both the method used for the inclusion of anharmonic effects and the level of electronic theory employed. However, semi-rigid molecules can be routinely analyzed with remarkable results, largely sufficient for interpretation and assignment tasks. Extension to flexible systems can be pursued by coupling reduced-dimensionality treatments of large-amplitude motions to GVPT2 for small-amplitude motions. In this connection, use of curvilinear in place of rectilinear coordinates is an appealing option. While work in this and related connections is underway in our laboratory, we think that already the present implementation offers a number of interesting perspectives for the study of molecular systems of current scientific and technological interest.

## Appendix

### A.1 Degeneracies in the Perturbative Treatment

One of the obvious issues in the VPT2 expansion based on the canonical representation is the risk of singularity arising from the degeneracy of the vibrational modes and the related harmonic states. Let us consider the calculation of the energy ε_*R*_ of a vibrational state |ψ_*R*_⟩ (the superscript “*C*” has been dropped for clarity). Following the Rayleigh–Schrödinger perturbation theory, the following relations are obtained:

with

39a

39b

39c

where the vectorial form |***v***_*R*_⟩ = |*v*_*R*,1_...*v*_*R*,*i*_...*v*_*R*,*N*_⟩ was preferred to represent the harmonic state |ψ_*R*_^(0)^⟩.

The problem lies in the second term of the right-hand side of eq 39c since if |***v***_*R*_⟩ and |***v***_*S*_⟩ are degenerate, the denominator would be null. For the sake of simplicity but without loss of generality, let us consider that both states involve the same set of degenerate modes, {*s*_1_, *s*_2_}. In order to have ε_*R*_^(0)^ = ε_*S*_^(0)^, the total number of quanta *v*_*s*_ = *v*_*s*_1__ + *v*_*s*_2__ must be kept constant, and the number of quanta involving non-degenerate modes cannot vary. Mathematically, this translates in an even number of bosonic ladder operators involving the degenerate modes. By construction,  is defined as

which implies that three creation/annihilation operations are carried out. Hence,  if |***v*****_*R*_**⟩ and |***v*****_*S*_**⟩ are degenerate. In other words, the representation matrix of  over the degenerate state basis is null so that the canonical representation can be safely applied to treat systems with degenerate modes.

## B.1 Calculation of Properties in the Polar Representation

In the following, we will describe the theoretical framework underlying the calculation of molecular properties (including transition ones) in the polar representation. Let us point out that even if the derivation reported below focuses on symmetric and linear tops, it still holds for systems presenting threefold degenerate vibrations.

Let us consider the operator Θ̂ describing a molecular property depending on normal coordinates or their conjugate momenta. Following previous works,^34,47,98^ a generic element of the representation matrix of Θ̂ over the canonical states can be written as

40

The perturbative expansion of both Θ̂ and canonical states enables to write a generic element Θ_*R*;*S*_^*C*^ as

41

where

42

Similar expressions stand for the polar representation. Since we already know that polar and canonical harmonic wave functions are linked through the customary expression

43

we will focus on the first- and second-order corrections to the wave functions.

### B.1.1 Perturbative Corrections to the Polar States

#### B.1.1.1 First-Order Correction to the Wave Function

In the canonical representation, the first-order correction |ψ_*R*_^*C*(1)^⟩ to the *R*th state is given by

44

The diagonal elements of the first-order Hamiltonian over the harmonic states are null as well as the off-diagonal ones when two degenerate states are considered. As a matter of fact, we can define the matrix **H̅**^*C*^ as

45

so that eq 44 can be rewritten as

46

By defining |ψ^*C*(0)^⟩ and |ψ^*C*(1)^⟩, the vectors collecting respectively the harmonic states and the corresponding first-order corrections in the canonical representation, eq 46 can be recast in the matrix form

47

and a similar expression holds in the polar representation

48

The diagonal blocks of **H̅**^*P*^ and **H̅**^*C*^ are null, and the energy difference ε_*i*_^(0)^ – ε_*j*_^(0)^ reported above is constant within an off-diagonal block. As a matter of fact, we obtain the following identity:

49

By substituting eqs 43 and 49 into eq 48, we obtain that

50

#### B.1.1.2 Second-Order Correction to the Wave Function

In the canonical representation, the second-order correction to the wave function is given by

51

Let us define *H̿*^*C*^ as

52

Equation 51 can be rewritten as follows:

53

Due to the presence of *H*_*SR*_^*C*(2)^ in eq 53, the terms for which the harmonic states |ψ_*R*_^*C*(0)^⟩ and |ψ_*S*_^*C*(0)^⟩ are degenerate must be excluded from the summation. From this, an analysis analogous to the one reported for the first-order correction leads to the following identity:

54

### B.2.1 Molecular Properties: From the Canonical to the Polar Representation

Let us now consider the general expression for the conversion of a molecular property at the second order of perturbation. An element of the representation matrix of the operator Θ̂ over the perturbed polar states is given by an expression similar to eq 41

55

where Θ^*P*(0)^, Θ^*P*(1)^, and Θ^*P*(2)^ are given by expressions similar to eq 42 that, recast in the matrix form, have the following expressions in the polar representation:

56

where the subscript *N* indicates a compact matrix notation for the normalization term.

By combining eqs 43, 50 and 54 with eq 56, the following identity is obtained:

57

where ⟨**Θ**⟩^*P*^ and ⟨**Θ**⟩^*C*^ are respectively the representation matrices of the operator Θ̂ over the perturbed polar and canonical states. Consequently, the conversion of molecular properties at the anharmonic level is ruled by expressions similar to those used for the transformation of the contact-transformed Hamiltonian.

#### B.2.1.1 Transition Properties from the Ground State

Finally, let us consider the ground state as the initial state. It is worth mentioning that the matrix **P** only mixes polar and canonical states that are degenerate at the harmonic level so that the perturbed wave function associated to the ground state is independent of the representation

58

As a result, the row vector ⟨**Θ**⟩_0;*S*_^*P*^, containing the values of the transition property (e.g., a component of the dipole moment or polarizability) from the ground state to a set of states sharing the set of principal quantum numbers ***v***_*S*_, is related to its canonical counterpart ⟨**Θ**⟩_0;*S*_^*C*^ as follows:

59

or equivalently,

60

where in eq 60, ⟨**Θ**⟩_0;*S*_^*P*^ and ⟨**Θ**⟩_0;*S*_^*C*^ are column vectors.

## Supporting Information Available

Rotation matrices, transition energies and intensities, and ZPVE for systems containing doubly or triply degenerate vibrations, **χ** and **g** matrices, and -type doubling coefficients in terms of the canonical representation, and expression for the transition properties accounting for degeneracies (PDF)
