# Supplementary material for: General Perturb-Then-Diagonalize Model for the Vibrational Frequencies and Intensities of Molecules Belonging to Abelian and Non-Abelian Symmetry Groups
Source: J Chem Theory Comput. 2021 Jun 4;17(7):4332–58. doi: 10.1021/acs.jctc.1c00240 (PMC8280743; doi:10.1021/acs.jctc.1c00240)
Supplement: Supplementary file 1 — ct1c00240_si_001.pdf [file ct1c00240_si_001.pdf]

Supporting Information: General Perturb-Then-Diagonalize Model for the Vibrational Frequencies and Intensities of Molecules Belonging to Abelian and Non-Abelian Symmetry Groups

Marco Mendolicchio, Julien Bloino, and Vincenzo Barone*

Scuola Normale Superiore, Piazza dei Cavalieri 7, I-56126 Pisa

E-mail: vincenzo.barone@sns.it

Contents

S1 Construction of the rotation matrices for symmetric and linear tops **S-3**

S1.1 One-quanta states S-3

S1.2 Two-quanta states S-3

S1.3 Three-quanta states S-4

S2 Vibrational energies **S-5**

S2.1 χ^P and $\mathbf{g}$ matrices S-5

S2.2 Derivation of the χ^P and $\mathbf{g}$ matrices in the canonical formalism S-7

S2.3 Equivalence relations of the anharmonic canonical energies S-9

S3 ZPVE **S-12**

S4 Darling-Dennison resonances with shifted reference states **S-15**

S4.1 1-1 resonances S-15

S4.2 2-2 resonances S-16

S4.3 2-11 resonances S-17

S4.4 11-11 resonances S-17

S5 ℓ-type doubling **S-18**

S6 Transition intensities **S-20**

S6.1 Non-resonant equations S-21

S6.2 Resonant cases S-24

S7 States involving both doubly- and triply-degenerate vibrations **S-26**

S7.1 Fundamental band of a triply-degenerate mode S-26

S7.2 First overtones of triply-degenerate vibrations S-26

S7.3 Binary combinations between triply-degenerate vibrations S-27

S7.4 Binary combinations between triply- and doubly-degenerate vibrations . . . S-29

References **S-32**

S1 Construction of the rotation matrices for symmetric and linear tops

A set of vibrational states in the polar representation $|\psi_{\boldsymbol{v}}^P\rangle$ is related to the corresponding canonical one $|\psi_{\boldsymbol{v}}^C\rangle$ through a unitary matrix $\mathbf{P}_{\boldsymbol{v}}$:

$$|\psi_{\boldsymbol{v}}^P\rangle = \mathbf{P}_v^T |\psi_{\boldsymbol{v}}^C\rangle \tag{1}$$

In the following, the linear transformations for states involving at least one degenerate vibration are reported.

S1.1 One-quanta states

S1.1.1 Doubly-degenerate fundamentals

$$\begin{pmatrix} |\psi^P_{1_s,+1_s}\rangle \\ |\psi^P_{1_s,-1_s}\rangle \end{pmatrix} = -\frac{1}{\sqrt{2}} \begin{pmatrix} 1 & i \\ 1 & -i \end{pmatrix} \begin{pmatrix} |\psi^C_{1_{s_1}}\rangle \\ |\psi^C_{1_{s_2}}\rangle \end{pmatrix} \tag{2}$$

S1.2 Two-quanta states

S1.2.1 Doubly-degenerate two-quanta overtones

$$\begin{pmatrix} |\psi^P_{2_s,+2_s}\rangle \\ |\psi^P_{2_s,0_s}\rangle \\ |\psi^P_{2_s,-2_s}\rangle \end{pmatrix} = \frac{1}{\sqrt{2}} \begin{pmatrix} \frac{1}{\sqrt{2}} & i & -\frac{1}{\sqrt{2}} \\ -1 & 0 & -1 \\ \frac{1}{\sqrt{2}} & -i & -\frac{1}{\sqrt{2}} \end{pmatrix} \begin{pmatrix} |\psi^C_{2_{s_1}}\rangle \\ |\psi^C_{1_{s_1}1_{s_2}}\rangle \\ |\psi^C_{2_{s_2}}\rangle \end{pmatrix} \tag{3}$$

S1.2.2 Degenerate 1-1 combination bands

$$\begin{pmatrix} |\psi^P_{1_s1_t,+1_s+1_t}\rangle \\ |\psi^P_{1_s1_t,+1_s-1_t}\rangle \\ |\psi^P_{1_s1_t,-1_s+1_t}\rangle \\ |\psi^P_{1_s1_t,-1_s-1_t}\rangle \end{pmatrix} = \frac{1}{2} \begin{pmatrix} 1 & i & i & -1 \\ 1 & -i & i & 1 \\ 1 & i & -i & 1 \\ 1 & -i & -i & -1 \end{pmatrix} \begin{pmatrix} |\psi^C_{1_{s_1}1_{t_1}}\rangle \\ |\psi^C_{1_{s_1}1_{t_2}}\rangle \\ |\psi^C_{1_{s_2}1_{t_1}}\rangle \\ |\psi^C_{1_{s_2}1_{t_2}}\rangle \end{pmatrix} \tag{4}$$

S1.3 Three-quanta states

Since the states $|\psi^P_{2_m 1_s}\rangle$ and $|\psi^P_{1_m 1_n 1_s}\rangle$ transform as $|\psi^P_{1_s}\rangle$, $|\psi^P_{1_m 2_s}\rangle$ as $|\psi^P_{2_s}\rangle$, and $|\psi^P_{1_m 1_s 1_t}\rangle$ as $|\psi^P_{1_s 1_t}\rangle$, the only rotation matrices to be derived correspond to the states $|\psi^P_{3_s}\rangle$, $|\psi^P_{2_s 1_t}\rangle$ and $|\psi^P_{1_s 1_t 1_u}\rangle$.

S1.3.1 Degenerate three-quanta overtones $|\psi^P_{3_s}\rangle$

$$\begin{pmatrix} |\psi^P_{3_s,+3_s}\rangle \\ |\psi^P_{3_s,+1_s}\rangle \\ |\psi^P_{3_s,-1_s}\rangle \\ |\psi^P_{3_s,-3_s}\rangle \end{pmatrix} = \frac{\sqrt{3}}{2\sqrt{2}} \begin{pmatrix} 1/\sqrt{3} & -i/\sqrt{3} & i & -1 \\ 1 & i & i/\sqrt{3} & 1/\sqrt{3} \\ 1 & -i & -i/\sqrt{3} & 1/\sqrt{3} \\ 1/\sqrt{3} & i/\sqrt{3} & -i & -1 \end{pmatrix} \begin{pmatrix} |\psi^C_{3_{s_1}}\rangle \\ |\psi^C_{3_{s_2}}\rangle \\ |\psi^C_{2_{s_1} 1_{s_2}}\rangle \\ |\psi^C_{1_{s_1} 2_{s_2}}\rangle \end{pmatrix} \tag{5}$$

S1.3.2 Degenerate 2-1 combination bands $|\psi^P_{2_s 1_t}\rangle$

$$\begin{pmatrix} |\psi^P_{2_s 1_t,+2_s+1_t}\rangle \\ |\psi^P_{2_s 1_t,+2_s-1_t}\rangle \\ |\psi^P_{2_s 1_t,0_s+1_t}\rangle \\ |\psi^P_{2_s 1_t,0_s-1_t}\rangle \\ |\psi^P_{2_s 1_t,-2_s+1_t}\rangle \\ |\psi^P_{2_s 1_t,-2_s-1_t}\rangle \end{pmatrix} = -\frac{1}{2} \begin{pmatrix} 1/\sqrt{2} & i & -1/\sqrt{2} & i/\sqrt{2} & -1 & -i/\sqrt{2} \\ 1/\sqrt{2} & i & -1/\sqrt{2} & -i/\sqrt{2} & 1 & i/\sqrt{2} \\ -1 & 0 & -1 & -i & 0 & -i \\ -1 & 0 & -1 & i & 0 & i \\ 1/\sqrt{2} & -i & -1/\sqrt{2} & i/\sqrt{2} & 1 & -i/\sqrt{2} \\ 1/\sqrt{2} & -i & -1/\sqrt{2} & -i/\sqrt{2} & -1 & i/\sqrt{2} \end{pmatrix} \begin{pmatrix} |\psi^C_{2_{s_1} 1_{t_1}}\rangle \\ |\psi^C_{1_{s_1} 1_{s_2} 1_{t_1}}\rangle \\ |\psi^C_{2_{s_2} 1_{t_1}}\rangle \\ |\psi^C_{2_{s_1} 1_{t_2}}\rangle \\ |\psi^C_{1_{s_1} 1_{s_2} 1_{t_2}}\rangle \\ |\psi^C_{2_{s_2} 1_{t_2}}\rangle \end{pmatrix} \tag{6}$$

S1.3.3 Degenerate 1-1-1 combination bands $|\psi^P_{1_s 1_t 1_u}\rangle$

$$\begin{pmatrix} |\psi^P_{1_s 1_t 1_u,+1_s+1_t+1_u}\rangle \\ |\psi^P_{1_s 1_t 1_u,+1_s+1_t-1_u}\rangle \\ |\psi^P_{1_s 1_t 1_u,+1_s-1_t+1_u}\rangle \\ |\psi^P_{1_s 1_t 1_u,-1_s+1_t+1_u}\rangle \\ |\psi^P_{1_s 1_t 1_u,+1_s-1_t-1_u}\rangle \\ |\psi^P_{1_s 1_t 1_u,-1_s+1_t-1_u}\rangle \\ |\psi^P_{1_s 1_t 1_u,-1_s-1_t+1_u}\rangle \\ |\psi^P_{1_s 1_t 1_u,-1_s-1_t-1_u}\rangle \end{pmatrix} = -\frac{1}{2\sqrt{2}} \begin{pmatrix} 1 & i & i & -1 & i & -1 & -1 & -i \\ 1 & -i & i & 1 & i & 1 & -1 & i \\ 1 & i & -i & 1 & i & -1 & 1 & i \\ 1 & i & i & -1 & -i & 1 & 1 & i \\ 1 & -i & -i & -1 & i & 1 & 1 & -i \\ 1 & -i & i & 1 & -i & -1 & 1 & -i \\ 1 & i & -i & 1 & -i & 1 & -1 & -i \\ 1 & -i & -i & -1 & -i & -1 & -1 & i \end{pmatrix} \begin{pmatrix} |\psi^C_{1_{s_1} 1_{t_1} 1_{u_1}}\rangle \\ |\psi^C_{1_{s_1} 1_{t_1} 1_{u_2}}\rangle \\ |\psi^C_{1_{s_1} 1_{t_2} 1_{u_1}}\rangle \\ |\psi^C_{1_{s_1} 1_{t_2} 1_{u_2}}\rangle \\ |\psi^C_{1_{s_2} 1_{t_1} 1_{u_1}}\rangle \\ |\psi^C_{1_{s_2} 1_{t_1} 1_{u_2}}\rangle \\ |\psi^C_{1_{s_2} 1_{t_2} 1_{u_1}}\rangle \\ |\psi^C_{1_{s_2} 1_{t_2} 1_{u_2}}\rangle \end{pmatrix} \tag{7}$$

S2 Vibrational energies

All elements of the $\boldsymbol{\chi}^P$ matrix and the diagonal ones of the $\mathbf{g}$ matrix can be obtained directly from the $\boldsymbol{\chi}^C$ matrix. In this section, the expressions of $\boldsymbol{\chi}^P$ and $\mathbf{g}$ are reported as done in Ref.[S1] Next, the expressions allowing the conversion from the canonical to the polar representation are discussed. The correctness of the $\boldsymbol{\chi}^C$ matrix, which can be used either for anharmonic calculations in the canonical representation, or as starting point for the evaluation of the $\boldsymbol{\chi}^P$ and $\mathbf{g}$ matrices, can be checked through specific equivalencies between the canonical vibrational energies, which are reported in Section S2.3.

S2.1 $\boldsymbol{\chi}^P$ and g matrices

In the polar representation, the matrices containing the anharmonic contributions, $\boldsymbol{\chi}^P$ and $\mathbf{g}$, are defined as,[S1]

$$16\chi^P_{mm} = \mathrm{f}_{mmmm} - \frac{5}{3}\frac{\mathrm{f}_{mmm}{}^2}{\omega_m} - \sum_{\substack{n=1\\(n\neq m)}}^{N'} \frac{\mathrm{f}_{mmn}{}^2(8\omega_m^2 - 3\omega_n^2)}{\omega_n(4\omega_m^2 - \omega_n^2)}$$

$$4\chi^P_{mn} = \mathrm{f}_{mmnn} - \frac{\mathrm{f}_{mmm}\mathrm{f}_{mnn}}{\omega_n} - \frac{\mathrm{f}_{mmn}\mathrm{f}_{nnn}}{\omega_n} - 2\frac{\mathrm{f}_{mmn}{}^2\omega_m}{4\omega_m^2 - \omega_n^2} - 2\frac{\mathrm{f}_{mnn}{}^2\omega_n}{4\omega_n^2 - \omega_m^2}$$
$$+ \sum_{\substack{o=1\\(o\neq m,n)}}^{N'} \left[\frac{2\mathrm{f}_{mno}{}^2(\omega_m^2 + \omega_n^2 - \omega_o^2)}{\Delta_{mno}} - \frac{\mathrm{f}_{mmo}\mathrm{f}_{nno}}{\omega_o}\right] + 4\sum_{\tau=x,y,z} B^e_\tau\{\zeta_{mn,\tau}\}^2 \left(\frac{\omega_m^2 + \omega_n^2}{\omega_m\omega_n}\right)$$

$$4\chi^P_{ms} = \mathrm{f}_{mmss} - \frac{\mathrm{f}_{mmm}\mathrm{f}^{(I)}_{mss}}{\omega_m} - 2\sum_\sigma \frac{\{\mathrm{f}^{(\sigma)}_{mss}\}^2\omega_s}{4\omega_s^2 - \omega_m^2} - \sum_{\substack{n=1\\(n\neq m)}}^{N'} \frac{\mathrm{f}_{mmn}\mathrm{f}^{(I)}_{nss}}{\omega_n}$$
$$+ \ 2\sum_{\substack{t=1\\(t\neq s)}}^{N''}\sum_\sigma \frac{\{\mathrm{f}^{(\sigma)}_{mst}\}^2\omega_t(\omega_m^2 + \omega_s^2 - \omega_t^2)}{\Delta_{mst}} + 4B^e_x[\{\zeta^{(I)}_{ms}\}^2 + \{\zeta^{(II)}_{ms}\}^2] \left(\frac{\omega_m^2 + \omega_s^2}{\omega_m\omega_s}\right)$$

$$16\chi^P_{ss} = \sum_{\sigma\leq III} \mathrm{f}^{(\sigma)}_{ssss} - \frac{5}{16}\sum_\sigma \frac{\{\mathrm{f}^{(\sigma)}_{sss}\}^2}{\omega_s} - \sum_{m=1}^{N'}\sum_\sigma \frac{\delta'_\sigma\{\mathrm{f}^{(\sigma)}_{mss}\}^2(8\omega_s^2 - 3\omega_m^2)}{\omega_m(4\omega_s^2 - \omega_m^2)} - \sum_{\substack{t=1\\(t\neq s)}}^{N''}\sum_\sigma \frac{\{\mathrm{f}^{(\sigma)}_{sst}\}^2(8\omega_s^2 - 3\omega_t^2)}{\omega_t(4\omega_s^2 - \omega_t^2)}$$

$$4\chi^P_{st} = \sum_{\sigma\leq VII} \delta'_\sigma \mathrm{f}^{(\sigma)}_{sstt} - 2\sum_\sigma \frac{\{\mathrm{f}^{(\sigma)}_{sst}\}^2\omega_s}{4\omega_s^2 - \omega_t^2} - 2\sum_\sigma \frac{\{\mathrm{f}^{(\sigma)}_{stt}\}^2\omega_t}{4\omega_t^2 - \omega_s^2}$$
$$+ \sum_{m=1}^{N'}\left[\sum_\sigma \frac{\{\mathrm{f}^{(\sigma)}_{mst}\}^2\omega_m(\omega_s^2 + \omega_t^2 - \omega_m^2)}{\Delta_{mst}} - \frac{\mathrm{f}^{(I)}_{mss}\mathrm{f}^{(I)}_{mtt}}{\omega_m}\right] + 2\sum_{\substack{u=1\\(u\neq s,t)}}^{N''}\sum_\sigma \frac{\{\mathrm{f}^{(\sigma)}_{stu}\}^2\omega_u(\omega_s^2 + \omega_t^2 - \omega_u^2)}{\Delta_{stu}}$$
$$+ \ 2\left\{B^e_z[\{\zeta^{(I)}_{st}\}^2 + \{\zeta^{(II)}_{st}\}^2] + B^e_x[\{\zeta^{(III)}_{st}\}^2 + \{\zeta^{(IV)}_{st}\}^2]\right\}\left(\frac{\omega_s^2 + \omega_t^2}{\omega_s\omega_t}\right)$$

$$16g_{ss} = -\frac{1}{3}\sum_{\sigma\leq III} \mathrm{f}^{(\sigma)}_{ssss} + \frac{7}{3}\sum_\sigma \frac{\{\mathrm{f}^{(\sigma)}_{sss}\}^2}{\omega_s} + \sum_{m=1}^{N'}\left[-\frac{\{\mathrm{f}^{(I)}_{mss}\}^2\omega_m}{4\omega_s^2 - \omega_m^2} + \frac{[\{\mathrm{f}^{(III)}_{mss}\}^2 + \{\mathrm{f}^{(IV)}_{mss}\}^2](8\omega_s^2 - \omega_m^2)}{2\omega_m(4\omega_s^2 - \omega_m^2)}\right]$$
$$+ \sum_{\substack{t=1\\(t\neq s)}}^{N'}\sum_\sigma \frac{\{\mathrm{f}^{(\sigma)}_{sst}\}^2(8\omega_s^2 - \omega_t^2)}{\omega_t(4\omega_s^2 - \omega_t^2)} + B^e_z\{\zeta^{(I)}_{ss}\}^2$$

$$g_{st} = \sum_{m=1}^{N'}\sum_\sigma \frac{\delta''_\sigma\{\mathrm{f}^{(\sigma)}_{mst}\}^2\omega_m\omega_s\omega_t}{2\Delta_{mst}} - \sum_{\substack{u=1\\(u\neq s,t)}}^{N''}\sum_\sigma \frac{\{\mathrm{f}^{(\sigma)}_{stu}\omega_s\omega_t\omega_u\}^2}{\Delta_{stu}} + \sum_\sigma\left[\frac{\delta''_\sigma\{\mathrm{f}^{(\sigma)}_{sst}\}^2\omega_s^2}{\omega_t(4\omega_s^2 - \omega_t^2)} + \frac{\delta''_\sigma\{\mathrm{f}^{(\sigma)}_{stt}\}^2\omega_t^2}{\omega_s(4\omega_t^2 - \omega_s^2)}\right]$$
$$+ B^e_x[(s_x\cdot 1 - s'_y\cdot 1)\{\zeta^{(III)}_{st}\}^2 + (s_y\cdot 1 - s'_x\cdot 1)\{\zeta^{(IV)}_{st}\}^2] + B^e_z[\{\zeta^{(I)}_{st}\}^2 + \{\zeta^{(II)}_{st}\}^2 + 2\zeta^{(I)}_{ss}\zeta^{(I)}_{tt}]$$

(8)

where f_{ijk} and f_{ijkl} are respectively the dimensionless cubic and quartic force constants, $\zeta_{ij,\tau}$ are the Coriolis interaction parameters, B_τ^{eq} is the equilibrium rotational constant along the τ axis and Δ_{ijk} is defined as follows,

$$\Delta_{ijk} = \omega_i^4 + \omega_j^4 + \omega_k^4 - 2(\omega_i^2\omega_j^2 + \omega_i^2\omega_k^2 + \omega_j^2\omega_k^2) \tag{9}$$

S2.2 Derivation of the $\boldsymbol{\chi}^P$ and g matrices in the canonical formalism

The theoretical derivation reported in this work shows that the energies in the polar representation can be obtained from the canonical ones without the actual determination of additional intermediate quantities like the anharmonic $\boldsymbol{\chi}^P$ and **g** matrices. Nevertheless, being able to derive them has several advantages. From the perspective of an implementation, it gives the possibility to check that there is no implementation error. But beyond this technical aspect, these matrices offer several practical features. As stated above, **g** gives information on the splitting of the degenerate states and can thus help analyze the contribution to the splitting of the bands. $\boldsymbol{\chi}$ contains the bulk of the anharmonic correction, so it is possible to assess the couplings between modes through the components of the matrix and their contribution to the anharmonic effects.

Let us anticipate that both $\boldsymbol{\chi}^P$ matrix and the diagonal elements of the **g** matrix (g_{ss}) can be obtained from the $\boldsymbol{\chi}^C$ matrix alone, whose elements have the following properties:

$$\begin{aligned}
\chi^C_{s_1s_1} &= \chi^C_{s_2s_2}\\
\chi^C_{ms_1} &= \chi^C_{ms_2}\\
\chi^C_{s_1t_1} &= \chi^C_{s_2t_2}\\
\chi^C_{s_1t_2} &= \chi^C_{s_2t_1}
\end{aligned} \tag{10}$$

Through the application of the rotation matrices to degenerate fundamentals $|\psi^P_{1_s,\pm 1_s}\rangle$ and binary combination bands $|\psi^P_{1_m 1_s,\pm 1_s}\rangle$, $|\psi^P_{1_s 1_t,\pm 1_s \pm 1_t}\rangle$ and $|\psi^P_{1_s 1_t,\pm 1_s \mp 1_t}\rangle$, it is possible to retrieve a set of simple expressions enabling the full calculation of $\boldsymbol{\chi}^P$ from $\boldsymbol{\chi}^C$:

$$\begin{aligned}
\chi^P_{mn} &= \chi^C_{mn} \\
\chi^P_{ss} &= \chi^C_{s_1 s_1} = \chi^C_{s_2 s_2} \\
\chi^P_{ms} &= \chi^C_{m s_1} = \chi^C_{m s_2} \\
\chi^P_{st} &= \frac{\chi^C_{s_1 t_1} + \chi^C_{s_1 t_2}}{2} = \frac{\chi^C_{s_2 t_1} + \chi^C_{s_2 t_2}}{2}
\end{aligned} \tag{11}$$

In addition, the closure relation of anharmonic energies can be used in conjunction with Eq. 11 to obtain an explicit formula for the g_{ss} terms:

$$\mathrm{g}_{ss} = \frac{1}{2}\chi^C_{s_1 s_2} - \chi^C_{s_1 s_1} = \frac{1}{2}\chi^C_{s_1 s_2} - \chi^C_{s_2 s_2} \tag{12}$$

Let us now consider the g_{st} terms, for which an explicit expression in terms of the $\boldsymbol{\chi}^C$ matrix is not available. By recalling that

$$\begin{aligned}
\varepsilon^P_{1_s 1_t,\pm 1_t \pm 1_t} &= \varepsilon^P_{1_s,\pm 1_s} + \varepsilon^P_{1_t,\pm 1_t} + g_{st} \\
\varepsilon^P_{1_s 1_t,\pm 1_t \mp 1_t} &= \varepsilon^P_{1_s,\pm 1_s} + \varepsilon^P_{1_t,\pm 1_t} - g_{st}
\end{aligned} \tag{13}$$

g_{st} can be expressed as half the difference between the energies $\varepsilon^P_{1_s,1_t;\pm 1,\pm 1}$ and $\varepsilon^P_{1_s,1_t;\pm 1,\mp 1}$, which in turn can be developed in terms of canonical energies and Darling–Dennison couplings, leading to the following expression:

$$\mathrm{g}_{st} = \frac{\langle \psi^C_{1_{s_1} 1_{t_2}} | \tilde{\mathcal{H}} | \psi^C_{1_{s_2} 1_{t_1}} \rangle - \langle \psi^C_{1_{s_1} 1_{t_1}} | \tilde{\mathcal{H}} | \psi^C_{1_{s_2} 1_{t_2}} \rangle}{2} \tag{14}$$

Let us remark that Eqs. 11, 12 and 14 are formally equivalent to Eq. 8. However,

the calculation of both $\boldsymbol{\chi}^P$ and $\mathbf{g}$ matrices through the procedure described above does not require the special definitions of anharmonic force field constants based on the molecular symmetry as done in Refs.[S1–S3]

S2.3 Equivalence relations of the anharmonic canonical energies

The properties of the $\boldsymbol{\chi}^C$ matrix introduced in Eq. 10 can be used to establish equivalencies among the anharmonic (transition) energies of the canonical states degenerate at the harmonic level.

The canonical energies at the anharmonic level are calculated through the following expression,

$$\varepsilon_R^C = \varepsilon_0^C + \sum_{i=1}^{N} v_{R,i}\omega_i + \sum_{i=1}^{N}\sum_{j=i}^{N} \chi_{ij}^C \left[v_{R,i}v_{R,j} + \frac{1}{2}\left(v_{R,i} + v_{R,j}\right)\right] \tag{15}$$

where ε_0^C is the canonical ZPVE and the elements of the $\boldsymbol{\chi}^C$ matrix are given by,

$$\begin{aligned} 16\chi_{ii}^C &= \mathrm{f}_{iiii} - \frac{5}{3}\frac{{\mathrm{f}_{iii}}^2}{\omega_i^2} - \sum_{\substack{j=1\\(j\neq i)}}^{N} \frac{(8\omega_i^2 - 3\omega_j^2){\mathrm{f}_{iij}}^2}{\omega_j(4\omega_i^2 - \omega_j^2)} \\ 4\chi_{ij}^C &= \mathrm{f}_{iijj} - \frac{2\omega_i{\mathrm{f}_{iij}}^2}{(4\omega_i^2 - \omega_j^2)} - \frac{2\omega_j{\mathrm{f}_{ijj}}^2}{(4\omega_j^2 - \omega_i^2)} - \frac{\mathrm{f}_{iii}\mathrm{f}_{ijj}}{\omega_i} - \frac{\mathrm{f}_{iij}\mathrm{f}_{jjj}}{\omega_j} \\ &+ \sum_{\substack{k=1\\(k\neq i,j)}}^{N} \left[\frac{2\omega_k(\omega_i^2 + \omega_j^2 - \omega_k^2){\mathrm{f}_{ijk}}^2}{\Delta_{ijk}} - \frac{\mathrm{f}_{iik}\mathrm{f}_{jjk}}{\omega_k}\right] + \frac{4(\omega_i^2 + \omega_j^2)}{\omega_i\omega_j} \sum_{\tau=x,y,z} B_\tau^{\mathrm{eq}}\{\zeta_{ij,\tau}\}^2 \end{aligned} \tag{16}$$

Considering states up to three quanta, the following expressions of the transition energies $\nu_R = \varepsilon_R^C - \varepsilon_0^C$ can be obtained:

- Fundamental bands:

$$\nu_{1_i} = \omega_i + 2\chi_{ii}^C + \frac{1}{2}\sum_{\substack{j=1\\(j\neq i)}}^{N} \chi_{ij}^C \tag{17}$$

- Overtone bands:

$$\begin{aligned} \nu_{2_i}^C &= 2\nu_i^C + 2\chi_{ii}^C \\ \nu_{3_i}^C &= 3\nu_i^C + 6\chi_{ii}^C \end{aligned} \tag{18}$$

- Combination bands:

$$\begin{aligned} \nu_{1_i 1_j}^C &= \nu_i^C + \nu_j^C + \chi_{ij}^C \\ \nu_{2_i 1_j}^C &= \nu_{2_i}^C + \nu_{1_i}^C + 2\chi_{ij}^C \\ \nu_{1_i 1_j 1_k}^C &= \nu_{1_i}^C + \nu_{1_j}^C + \nu_{1_k}^C + \chi_{ij}^C + \chi_{jk}^C + \chi_{ik}^C \end{aligned} \tag{19}$$

Eq. 11 can be used in conjunction with Eqs. 17, 18 and 19 to obtain the set of identities reported in the following:

$$
\begin{aligned}
\varepsilon^C_{1_{s_1}} &= \varepsilon^C_{1_{s_2}} \\
\varepsilon^C_{2_{s_1}} &= \varepsilon^C_{2_{s_2}} \\
\varepsilon^C_{3_{s_1}} &= \varepsilon^C_{3_{s_2}} \\
\varepsilon^C_{1_m 1_{s_1}} &= \varepsilon^C_{1_m 1_{s_2}} \\
\varepsilon^C_{1_{s_1} 1_{t_1}} &= \varepsilon^C_{1_{s_2} 1_{t_2}} \\
\varepsilon^C_{1_{s_1} 1_{t_2}} &= \varepsilon^C_{1_{s_2} 1_{t_1}} \\
\varepsilon^C_{2_m 1_{s_1}} &= \varepsilon^C_{2_m 1_{s_2}} \\
\varepsilon^C_{1_m 2_{s_1}} &= \varepsilon^C_{1_m 2_{s_2}} \\
\varepsilon^C_{2_{s_1} 1_{t_1}} &= \varepsilon^C_{2_{s_2} 1_{t_2}} \\
\varepsilon^C_{2_{s_1} 1_{t_2}} &= \varepsilon^C_{2_{s_2} 1_{t_1}} \\
\varepsilon^C_{1_{s_1} 1_{s_2} 1_{t_1}} &= \varepsilon^C_{1_{s_1} 1_{s_2} 1_{t_2}} \\
\varepsilon^C_{1_m 1_n 1_{s_1}} &= \varepsilon^C_{1_m 1_n 1_{s_2}} \\
\varepsilon^C_{1_m 1_{s_1} 1_{t_1}} &= \varepsilon^C_{1_m 1_{s_2} 1_{t_2}} \\
\varepsilon^C_{1_m 1_{s_1} 1_{t_2}} &= \varepsilon^C_{1_m 1_{s_2} 1_{t_1}} \\
\varepsilon^C_{1_{s_1} 1_{t_1} 1_{u_1}} &= \varepsilon^C_{1_{s_2} 1_{t_2} 1_{u_2}} \\
\varepsilon^C_{1_{s_1} 1_{t_2} 1_{u_2}} &= \varepsilon^C_{1_{s_2} 1_{t_1} 1_{u_1}} \\
\varepsilon^C_{1_{s_2} 1_{t_1} 1_{u_2}} &= \varepsilon^C_{1_{s_1} 1_{t_2} 1_{u_1}} \\
\varepsilon^C_{1_{s_2} 1_{t_2} 1_{u_1}} &= \varepsilon^C_{1_{s_1} 1_{t_1} 1_{u_2}}
\end{aligned}
\tag{20}
$$

S3 ZPVE

The expression of the resonance-free zero-point vibrational energy (ZPVE) for asymmetric tops is[S4–S6]

$$\begin{aligned}\varepsilon_0^C = &\sum_{i=1}^{N}\frac{\omega_i}{2} + \sum_{i=1}^{N}\sum_{j=1}^{N}\frac{\mathrm{f}_{iijj}}{32} - \sum_{i=1}^{N}\sum_{j=1}^{N}\sum_{k=1}^{N}\left[\frac{\mathrm{f}_{iik}\mathrm{f}_{jjk}}{32\omega_k} + \frac{{\mathrm{f}_{ijk}}^2}{48(\omega_i+\omega_j+\omega_k)}\right] \\ &- \sum_{\tau=x,y,z}\frac{B_\tau^{\mathrm{eq}}}{4}\left[1 - \sum_{i=1}^{N-1}\sum_{j=i+1}^{N}\{\zeta_{ij,\tau}\}^2\frac{(\omega_i-\omega_j)^2}{\omega_i\omega_j}\right]\end{aligned} \tag{21}$$

where f_{ijk} and f_{ijkl} are respectively the cubic and quartic force constants, while $\zeta_{ij,\tau}$ represent the Coriolis interaction parameters.

In this section, we will show that Eq. 21 can be used also for linear and symmetric tops, i.e.,

$$\varepsilon_0^C = \varepsilon_0^P \tag{22}$$

In order to demonstrate this equality, Eq. 21 has been rewritten according to the rules proposed by Amat and Henry[S7–S10] based on the equivalencies reported in Ref.[S1,S3] This procedure has to be repeated for all sets of symmetry rules, which are related in part to the order of the principal symmetry axis C_n. While straightforward, it is a relatively tedious work. For this reason, and as an illustration, let us focus on a single term,

$$\begin{aligned}\sum_{i=1}^{N}\sum_{j=1}^{N}\frac{\mathrm{f}_{iijj}}{32} = &\sum_{m=1}^{N'}\sum_{n=1}^{N'}\frac{\mathrm{f}_{mmnn}}{32} + \sum_{m=1}^{N'}\sum_{s=1}^{N''}\frac{\mathrm{f}_{mms_1s_1}+\mathrm{f}_{mms_2s_2}}{32} \\ &+ \sum_{s=1}^{N''}\sum_{t=1}^{N''}\frac{\mathrm{f}_{s:1s_1t_1t_1}+\mathrm{f}_{s_1s_1t_2t_2}+\mathrm{f}_{s_2s_2t_1t_1}+\mathrm{f}_{s_2s_2t_2t_2}}{32}\end{aligned} \tag{23}$$

By comparison with the ZPVE in the polar representation,[S1]

$$
\begin{aligned}
\varepsilon_0^P =& \frac{1}{2}\sum_{i=1}^{N}\omega_i d_i + \frac{1}{32}\sum_{m=1}^{N'}\sum_{n=1}^{N'} \mathrm{f}_{mmnn} + \frac{1}{12}\sum_{s=1}^{N''}\sum_{\sigma\leq III}\delta_\sigma \mathrm{f}_{ssss}^{(\sigma)} + \frac{1}{8}\sum_{m=1}^{N'}\sum_{s=1}^{N''}\mathrm{f}_{mmss} + \frac{1}{8}\sum_{s=1}^{N''}\sum_{\substack{t=1\\(t\neq s)}}^{N''}\sum_{\sigma\leq VII}\delta'_\sigma \mathrm{f}_{sstt}^{(\sigma)} \\
& - \sum_{m=1}^{N'}\sum_{n=1}^{N'}\sum_{o=1}^{N'}\left[\frac{\mathrm{f}_{mmo}\mathrm{f}_{nno}}{32\omega_o} + \frac{\mathrm{f}_{mno}^2}{48(\omega_m+\omega_n+\omega_o)}\right] - \frac{1}{36}\sum_{s=1}^{N''}\sum_{\sigma}\{\mathrm{f}_{sss}^{(\sigma)}\}^2 - \sum_{m=1}^{N'}\sum_{s=1}^{N''}\left[\frac{\{\mathrm{f}_{mss}^{(I)}\}^2(\omega_m+\omega_s)}{4\omega_m(2\omega_s+\omega_m)}\right. \\
& + \frac{\{\mathrm{f}_{mss}^{(III)}\}^2+\{\mathrm{f}_{mss}^{(IV)}\}^2}{8(2\omega_s+\omega_m)} + \frac{1}{8}\sum_{n=1}^{N'}\frac{\mathrm{f}_{mmn}\mathrm{f}_{nss}^{(I)}}{\omega_n} + \frac{1}{4}\sum_{t=s+1}^{N''}\left.\left(\frac{\mathrm{f}_{mss}^{(I)}\mathrm{f}_{mtt}^{(I)}}{\omega_m} + \sum_\sigma \frac{\{\mathrm{f}_{mst}^{(\sigma)}\}^2}{\omega_m+\omega_s+\omega_t}\right)\right] \\
& - \frac{1}{4}\sum_{s=1}^{N''}\sum_{\substack{t=1\\(t\neq s)}}^{N''}\sum_\sigma \frac{\{\mathrm{f}_{sst}^{(\sigma)}\}^2}{2\omega_s+\omega_t} - \frac{1}{2}\sum_{s=1}^{N''}\sum_{t=s+1}^{N''}\sum_{u=t+1}^{N''}\sum_\sigma \frac{\{\mathrm{f}_{stu}^{(\sigma)}\}^2}{\omega_s+\omega_t+\omega_u} - \frac{\Gamma}{4}\sum_{\tau=x,y,z}B_\tau^e \\
& + \frac{1}{4}\sum_{\tau=x,y,z}B_\tau^e\sum_{m=1}^{N'}\sum_{n=m+1}^{N'}\{\zeta_{mn,\tau}\}^2\left[\frac{\omega_m^2+\omega_n^2}{\omega_m\omega_n}-2\right] + \frac{B_x^e}{2}\sum_{m=1}^{N'}\sum_{s=1}^{N''}\left[\{\zeta_{ms}^{(I)}\}^2+\{\zeta_{ms}^{(II)}\}^2\right]\left[\frac{\omega_m^2+\omega_s^2}{\omega_m\omega_s}-2\right] \\
& + \sum_{s=1}^{N''}\sum_{t=s+1}^{N''}\left(\frac{B_z^e}{2}\Big[\{\zeta_{st}^{(I)}\}^2+\{\zeta_{st}^{(II)}\}^2\Big] + B_x^e\Big[\{\zeta_{st}^{(III)}\}^2+\{\zeta_{st}^{(IV)}\}^2\Big]\right)\left[\frac{\omega_s^2+\omega_t^2}{\omega_s\omega_t}-2\right]
\end{aligned}
\tag{24}
$$

the following equality must hold:

$$
\sum_{s=1}^{N''}\frac{\mathrm{f}_{s_1s_1s_1s_1}+\mathrm{f}_{s_2s_2s_2s_2}+2\mathrm{f}_{s_1s_1s_2s_2}}{32} = \frac{1}{12}\left(\mathrm{f}_{ssss}^{(I)} + \frac{3}{4}\Big[\mathrm{f}_{ssss}^{(II)}+\mathrm{f}_{ssss}^{(III)}\Big]\right) \tag{25}
$$

Based on the irreducible representation of each couple of degenerate modes s, two sets of conditions are possible,

I. $\mathrm{f}_{s_1s_1s_1s_1} = \mathrm{f}_{s_2s_2s_2s_2} = 3\mathrm{f}_{s_1s_1s_2s_2} = \mathrm{f}_{ssss}^{(I)}$

$\mathrm{f}_{ssss}^{(II)} = \mathrm{f}_{ssss}^{(III)} = 0$

II. $\mathrm{f}_{s_1s_1s_1s_1} = \mathrm{f}_{s_2s_2s_2s_2} = \mathrm{f}_{ssss}^{(II)}$

$\mathrm{f}_{s_1s_1s_2s_2} = \mathrm{f}_{ssss}^{(III)}$

$\mathrm{f}_{ssss}^{(I)} = 0$

These relations lead to

$$
\begin{aligned}
&\text{I.} \qquad \frac{\mathrm{f}_{s_1s_1s_1s_1} + \mathrm{f}_{s_2s_2s_2s_2} + 2\mathrm{f}_{s_1s_1s_2s_2}}{32} = \frac{\mathrm{f}^{(I)}_{ssss} + \mathrm{f}^{(I)}_{ssss} + 2\mathrm{f}^{(I)}_{ssss}/3}{32} = \frac{1}{12}\mathrm{f}^{(I)}_{ssss} \\
&\text{II.} \qquad \frac{\mathrm{f}_{s_1s_1s_1s_1} + \mathrm{f}_{s_2s_2s_2s_2} + 2\mathrm{f}_{s_1s_1s_2s_2}}{32} = \frac{\mathrm{f}^{(II)}_{ssss} + \mathrm{f}^{(II)}_{ssss} + 2\mathrm{f}^{(III)}_{ssss}}{32} = \frac{\mathrm{f}^{(II)}_{ssss} + \mathrm{f}^{(III)}_{ssss}}{16}
\end{aligned}
$$

Because the relations “I” and “II” are mutually exclusive, this can be generalized as,

$$
\sum_{s=1}^{N''} \frac{\mathrm{f}_{s_1s_1s_1s_1} + \mathrm{f}_{s_2s_2s_2s_2} + 2\mathrm{f}_{s_1s_1s_2s_2}}{32} = \frac{1}{12}\left(\mathrm{f}^{(I)}_{ssss} + \frac{3}{4}\left[\mathrm{f}^{(II)}_{ssss} + \mathrm{f}^{(III)}_{ssss}\right]\right)
$$

The same process can be repeated for all terms and for each symmetry group, leading systematically to Eq. 24 from Eq. 21. Hence, symmetry-specific formulas are unnecessary to compute the ZPVE of linear and symmetric tops.

S4 Darling-Dennison resonances with shifted reference states

In the first place, the expressions of the Darling-Dennison resonances in the canonical representation, derived by Rosnik and Polik,[S5] are reported. For clarity, the superscript C will be dropped in the following, and the notation $|\boldsymbol{v}\rangle = |v_1, \ldots v_i \ldots v_N\rangle$ will be used.

S4.1 1-1 resonances

S4.1.1 General expression

$$\langle v{+}1_i|\tilde{\mathcal{H}}|v{+}1_j\rangle = \frac{\sqrt{(v_i+1)(v_j+1)}}{2}\left\{\frac{3}{2}(v_i+1)\mathcal{K}_{ii;ji} + \frac{3}{2}(v_j+1)\mathcal{K}_{ij;jj} + \sum_{k\neq i,j}^{N}\left(v_k+\frac{1}{2}\right)\mathcal{K}_{ik;jk}\right\} \tag{26}$$

with

$$\begin{aligned}
\mathcal{K}_{ii;ji} &= \frac{\mathrm{f}_{iiij}}{6} - \frac{1}{24}\sum_{m=1}^{N}\mathrm{f}_{ijm}\mathrm{f}_{iim}\left[\frac{1}{2\omega_i+\omega_m} + \frac{1}{\omega_m-2\omega_i} + \frac{4}{\omega_m} + \frac{2}{\omega_j-\omega_i+\omega_m} + \frac{2}{\omega_i+\omega_m-\omega_j}\right.\\
&\quad\left. + \frac{1}{\omega_i+\omega_j+\omega_m} + \frac{1}{\omega_m-\omega_i-\omega_j}\right]\\
\mathcal{K}_{ij;jj} &= \frac{\mathrm{f}_{ijjj}}{6} - \frac{1}{24}\sum_{m=1}^{N}\mathrm{f}_{ijm}\mathrm{f}_{jjm}\left[\frac{1}{2\omega_j+\omega_m} + \frac{1}{\omega_m-2\omega_j} + \frac{4}{\omega_m} + \frac{2}{\omega_i-\omega_j+\omega_m} + \frac{2}{\omega_j+\omega_m-\omega_i}\right.\\
&\quad\left. + \frac{1}{\omega_i+\omega_j+\omega_m} + \frac{1}{\omega_m-\omega_i-\omega_j}\right]\\
\mathcal{K}_{ik;jk} &= \frac{\mathrm{f}_{ijkk}}{2} + 2\sum_{\tau=x,y,z}B^{eq}_{\tau}\frac{\zeta_{ik,\tau}\zeta_{jk,\tau}(\omega_i\omega_j+\omega_k^2)}{\omega_k\sqrt{\omega_i\omega_j}} - \frac{1}{8}\sum_{m=1}^{N}\mathrm{f}_{ikm}\mathrm{f}_{jkm}\left[\frac{1}{\omega_i+\omega_k+\omega_m} + \frac{1}{\omega_m-\omega_i-\omega_k}\right.\\
&\quad + \frac{1}{\omega_j+\omega_k+\omega_m} + \frac{1}{\omega_m-\omega_j-\omega_k} + \frac{1}{\omega_i+\omega_m-\omega_k} + \frac{1}{\omega_k+\omega_m-\omega_i} + \frac{1}{\omega_k+\omega_m-\omega_j}\\
&\quad\left. + \frac{1}{\omega_j+\omega_m-\omega_k}\right] - \frac{1}{8}\sum_{m=1}^{N}\mathrm{f}_{ijm}\mathrm{f}_{kkm}\left[\frac{1}{\omega_i+\omega_m-\omega_j} + \frac{1}{\omega_j+\omega_m-\omega_i} + \frac{2}{\omega_m}\right]
\end{aligned} \tag{27}$$

S4.1.2 Special cases: $\langle \boldsymbol{\nu}+2_i\,|\,\tilde{\boldsymbol{\mathcal{H}}}\,|\,\boldsymbol{\nu}+1_i+1_j\rangle$ and $\langle \boldsymbol{\nu}+1_i+1_j\,|\,\tilde{\boldsymbol{\mathcal{H}}}\,|\,\boldsymbol{\nu}+1_j+1_k\rangle$

The terms $\langle v+2_i\,|\,\tilde{\mathcal{H}}\,|\,v+1_i+1_j\rangle$ and $\langle v+1_i+1_j\,|\,\tilde{\mathcal{H}}\,|\,v+1_i+1_k\rangle$ required to obtain the expression of the ℓ-type doubling in terms of canonical quantities, correspond to resonances of types 2-11 and 11-11, respectively. However, they can be rewritten in the following form:

$$\begin{aligned}\langle v+2_i\,|\,\tilde{\mathcal{H}}\,|\,v+1_i+1_j\rangle &= \langle v'+1_i\,|\,\tilde{\mathcal{H}}\,|\,v'+1_j\rangle \\ \langle v+1_i+1_k\,|\,\tilde{\mathcal{H}}\,|\,v+1_j+1_k\rangle &= \langle v''+1_i\,|\,\tilde{\mathcal{H}}\,|\,v''+1_j\rangle\end{aligned} \tag{28}$$

where $|v'\rangle = |v+1_i\rangle$ and $|v''\rangle = |v+1_k\rangle$. As a matter of fact, Eq. (27) can be employed in both cases:

$$\langle v+2_i\,|\,\tilde{\mathcal{H}}\,|\,v+1_i+1_j\rangle = \frac{\sqrt{(v_i+2)(v_j+1)}}{2}\left\{\frac{3}{2}(v_i+2)\mathcal{K}_{ii;ji} + \frac{3}{2}(v_j+1)\mathcal{K}_{ij;jj} + \sum_{k\neq i,j}^{N}\left(v_k+\frac{1}{2}\right)\mathcal{K}_{ik;jk}\right\} \tag{29}$$

$$\begin{aligned}\langle v+1_i+1_k\,|\,\tilde{\mathcal{H}}\,|\,v+1_j+1_k\rangle = \frac{\sqrt{(v_i+1)(v_j+1)}}{2}&\left\{\frac{3}{2}(v_i+1)\mathcal{K}_{ii;ji} + \frac{3}{2}(v_j+1)\mathcal{K}_{ij;jj}\right.\\ &\left.+\sum_{l\neq i,j}^{N}\left(v_l+\delta_{kl}+\frac{1}{2}\right)\mathcal{K}_{il;jl}\right\}\end{aligned} \tag{30}$$

S4.2 2-2 resonances

$$\langle v+2_i\,|\,\tilde{\mathcal{H}}\,|\,v+2_k\rangle = \frac{\sqrt{(v_i+1)(v_i+2)(v_k+1)(v_k+2)}}{4}\mathcal{K}_{ii;kk} \tag{31}$$

with

$$\begin{aligned}\mathcal{K}_{ii;kk} = {} & \frac{\mathrm{f}_{iikk}}{4} - \sum_{\tau=x,y,z} B_\tau^{eq}\frac{\{\zeta_{ik,\tau}\}^2(\omega_i+\omega_k)^2}{\omega_i\omega_k} \\ & -\frac{1}{16}\sum_{m=1}^{N}\mathrm{f}_{iim}\mathrm{f}_{kkm}\left[\frac{1}{2\omega_i+\omega_m}+\frac{1}{\omega_m-2\omega_i}+\frac{1}{2\omega_k-\omega_m}+\frac{1}{\omega_m-2\omega_k}\right] \\ & -\frac{1}{4}\sum_{m=1}^{N}{\mathrm{f}_{ikm}}^2\left[\frac{1}{\omega_i-\omega_k+\omega_m}+\frac{1}{\omega_k-\omega_i+\omega_m}\right]\end{aligned} \tag{32}$$

S4.3 2-11 resonances

$$\langle v+2_i\,|\,\tilde{\mathcal{H}}\,|\,v+1_k+1_l\rangle = \frac{\sqrt{(v_i+1)(v_i+2)(v_k+1)(v_l+1)}}{4}\mathcal{K}_{ii;kl} \tag{33}$$

with

$$\begin{aligned}
\mathcal{K}_{ii;kl} &= \frac{\mathrm{f}_{iikl}}{2} - 2\sum_{\tau=x,y,z} B_\tau^{eq}\frac{\zeta_{ik,\tau}\zeta_{il,\tau}(\omega_i+\omega_k)(\omega_i+\omega_l)}{\omega_i\sqrt{\omega_k\omega_l}} \\
&+\ \frac{1}{4}\sum_{m=1}^{N}\mathrm{f}_{ikm}\mathrm{f}_{ilm}\left[\frac{1}{\omega_k-\omega_i-\omega_m}+\frac{1}{\omega_i-\omega_k-\omega_m}+\frac{1}{\omega_l-\omega_i-\omega_m}+\frac{1}{\omega_i-\omega_l-\omega_m}\right] \\
&-\ \frac{1}{8}\sum_{m=1}^{N}\mathrm{f}_{iim}\mathrm{f}_{klm}\left[\frac{1}{2\omega_i+\omega_m}+\frac{1}{\omega_m-2\omega_i}+\frac{1}{\omega_k+\omega_l+\omega_m}+\frac{1}{\omega_m-\omega_k-\omega_l}\right]
\end{aligned} \tag{34}$$

S4.4 11-11 resonances

$$\langle v+1_i+1_j\,|\,\tilde{\mathcal{H}}\,|\,v+1_k+1_l\rangle = \frac{\sqrt{(v_i+1)(v_j+1)(v_k+1)(v_l+1)}}{4}\mathcal{K}_{ij;kl} \tag{35}$$

with

$$\begin{aligned}
\mathcal{K}_{ij;kl} &= \mathrm{f}_{ijkl} + 2\sum_{\tau=x,y,z} B_\tau^{eq}\left[\frac{\zeta_{ij,\tau}\zeta_{kl,\tau}(\omega_i-\omega_j)(\omega_k-\omega_l) - \zeta_{ik,\tau}\zeta_{jl,\tau}(\omega_i+\omega_k)(\omega_j+\omega_l)}{\sqrt{\omega_i\omega_j\omega_k\omega_l}}\right. \\
&\left.-\frac{\zeta_{il,\tau}\zeta_{jk,\tau}(\omega_i+\omega_l)(\omega_j+\omega_k)}{\sqrt{\omega_i\omega_j\omega_k\omega_l}}\right] \\
&-\ \frac{1}{4}\sum_{m=1}^{N}\mathrm{f}_{ijm}\mathrm{f}_{klm}\left[\frac{1}{\omega_i+\omega_j+\omega_m}+\frac{1}{\omega_k+\omega_l+\omega_m}+\frac{1}{\omega_m-\omega_i-\omega_j}+\frac{1}{\omega_m-\omega_k-\omega_l}\right] \\
&+\ \frac{1}{4}\sum_{m=1}^{N}\mathrm{f}_{ikm}\mathrm{f}_{jlm}\left[\frac{1}{\omega_k-\omega_i-\omega_m}+\frac{1}{\omega_l-\omega_j-\omega_m}+\frac{1}{\omega_i-\omega_k-\omega_m}+\frac{1}{\omega_j-\omega_l-\omega_m}\right] \\
&+\ \frac{1}{4}\sum_{m=1}^{N}\mathrm{f}_{ilm}\mathrm{f}_{jkm}\left[\frac{1}{\omega_l-\omega_i-\omega_m}+\frac{1}{\omega_i-\omega_l-\omega_m}+\frac{1}{\omega_j-\omega_k-\omega_m}+\frac{1}{\omega_k-\omega_j-\omega_m}\right]
\end{aligned} \tag{36}$$

S5 $\boldsymbol{\ell}$-type doubling

Let us first consider the $U_s^{\pm}$ term, which can be expressed in terms of canonical energies and off-diagonal coupling terms as follows,

$$U_s^{\pm} = \frac{1}{16}\left[\varepsilon^C_{2_{s_1}} - \varepsilon^C_{1_{s_1}1_{s_2}} - \langle\psi^C_{2_{s_1}}|\tilde{\mathcal{H}}|\psi_{2_{s_2}}\rangle\right] \pm \frac{i}{8\sqrt{2}}\left[\langle\psi^C_{2_{s_1}}|\tilde{\mathcal{H}}|\psi^C_{1_{s_1}1_{s_2}}\rangle - \langle\psi^C_{2_{s_2}}|\tilde{\mathcal{H}}|\psi^C_{1_{s_1}1_{s_2}}\rangle\right] \tag{37}$$

When the order of the principal symmetry axis n is a multiple of 4, both real and imaginary parts of Eq. 37 vanish, and upon rearrangement we obtain an alternative formula for the calculation of g_{ss}:

$$g_{ss} = -\frac{1}{2}\langle\psi^C_{2_{s_1}}|\tilde{\mathcal{H}}|\psi^C_{2_{s_2}}\rangle \qquad \text{when } n \text{ is not a multiple of 4} \tag{38}$$

The expressions of $R_{st}^{\pm}$ and $S_{st}^{\pm}$ in terms of canonical quantities can be expressed as reported below,

$$R_{st}^{\pm} = \frac{1}{4}\langle\psi^P_{1_s1_t,\mp1_s\pm1_t}|\tilde{\mathcal{H}}|\psi^P_{1_s1_t,\pm1_s\mp1_t}\rangle = A_{st} + B_{st} \pm iC_{st} \tag{39a}$$

$$S_{st}^{\pm} = \frac{1}{4}\langle\psi^P_{1_s1_t,\mp1_s\mp1_t}|\tilde{\mathcal{H}}|\psi^P_{1_s1_t,\pm1_s\pm1_t}\rangle = A_{st} - B_{st} \mp iD_{st} \tag{39b}$$

where the following aliases have been introduced for the sake of readability:

$$
\begin{aligned}
A_{st} =& \frac{1}{4}\left[\varepsilon^{C}_{1_{s_1}1_{t_1}} + \varepsilon^{C}_{1_{s_2}1_{t_2}} - \varepsilon^{C}_{1_{s_1}1_{t_2}} - \varepsilon^{C}_{1_{s_2}1_{t_1}}\right]\\
B_{st} =& \frac{1}{2}\left[\langle\psi^{C}_{1_{s_1}1_{t_1}}|\tilde{\mathcal{H}}|\psi^{C}_{1_{s_2}1_{t_2}}\rangle + \langle\psi^{C}_{1_{s_1}1_{t_2}}|\tilde{\mathcal{H}}|\psi^{C}_{1_{s_2}1_{t_1}}\rangle\right]\\
C_{st} =& \frac{1}{2}\left[\langle\psi^{C}_{1_{s_1}1_{t_1}}|\tilde{\mathcal{H}}|\psi^{C}_{1_{s_1}1_{t_2}}\rangle + \langle\psi^{C}_{1_{s_2}1_{t_2}}|\tilde{\mathcal{H}}|\psi^{C}_{1_{s_1}1_{t_2}}\rangle - \langle\psi^{C}_{1_{s_1}1_{t_1}}|\tilde{\mathcal{H}}|\psi^{C}_{1_{s_2}1_{t_1}}\rangle - \langle\psi^{C}_{1_{s_2}1_{t_2}}|\tilde{\mathcal{H}}|\psi^{C}_{1_{s_2}1_{t_1}}\rangle\right]\\
D_{st} =& \frac{1}{2}\left[\langle\psi^{C}_{1_{s_1}1_{t_1}}|\tilde{\mathcal{H}}|\psi^{C}_{1_{s_1}1_{t_2}}\rangle - \langle\psi^{C}_{1_{s_2}1_{t_2}}|\tilde{\mathcal{H}}|\psi^{C}_{1_{s_1}1_{t_2}}\rangle + \langle\psi^{C}_{1_{s_1}1_{t_1}}|\tilde{\mathcal{H}}|\psi^{C}_{1_{s_2}1_{t_1}}\rangle - \langle\psi^{C}_{1_{s_2}1_{t_2}}|\tilde{\mathcal{H}}|\psi^{C}_{1_{s_2}1_{t_1}}\rangle\right]
\end{aligned}
\tag{40}
$$

Let us stress that the terms included in both C_{st} and D_{st} are 11-11 resonances in which bra and ket share one quantum of the same mode. As a result they can be evaluated through the expression for 1-1 resonances,[S5] considering a shifted reference state. Conversely, the term B_{st} contains actual 11-11 Darling-Dennison coupling terms involving the quartic force constants $\mathrm{f}_{s_1s_2t_1t_2}$, which are not available through one-step linear differentiations. However, there is a special case in which this issue can be bypassed, that is when n is odd. Under such circumstances, $S^{\pm}_{st}$ is null, so that $A_{st} = B_{st}$ and $D_{st} = 0$. The former equality can be used to rewrite $R^{\pm}_{st}$ in a form devoid of quartic force constants with four different indexes,

$$
R^{\pm}_{st} = 2A_{st} \pm iC_{st} \qquad \text{when } n \text{ is odd} \tag{41}
$$

since A_{st} is only dependent on canonical energies.

S6 Transition intensities

The definition of the transition moments depends on the final state and becomes slightly different if the final state is in resonance with one of the intermediate states during the development.

We report here the formulas in absence of resonance (non-resonant) and in presence of resonances (resonant). To be general, the equations are written for an arbitrary property, which can be a function of the normal coordinates ($S = 1$) or their conjugate momenta ($S = -1$). The definition of s_0, s_1, s_2, and $\mathbf{P}_0$, $\mathbf{P}_i$, $\mathbf{P}_{ij}$, $\mathbf{P}_{ijk}$ depends on the property. Interested readers are referred to Refs.[S11,S12] for examples of equivalencies with actual properties. As a final comment, and as already discussed in Ref.,[S13] transition moments at the VPT2 level for four-quanta excitations are null by construction, and unavailable for higher-quanta ones.

S6.1 Non-resonant equations

S6.1.1 1-quanta transitions: fundamentals

$$
\begin{aligned}
\langle \boldsymbol{P} \rangle_{0,1_i} &= s_0 \times \mathrm{S} \times \boldsymbol{P}_i + \frac{s_2}{2} \sum_{j=1}^{N} \{ \boldsymbol{P}_{jij} + \boldsymbol{P}_{ijj} + \mathrm{S}\boldsymbol{P}_{jji} \} - \frac{s_0}{8} \sum_{j=1}^{N} \sum_{k=1}^{N} \mathrm{f}_{ijkk} \boldsymbol{P}_j \left[\frac{1}{\omega_i + \omega_j} - \frac{\mathrm{S}(1-\delta_{ij})}{\omega_i - \omega_j} \right] \\
&- \frac{s_1}{8} \sum_{j=1}^{N} \sum_{k=1}^{N} \left\{ \mathrm{f}_{ijk} \left(\boldsymbol{P}_{jk} + \boldsymbol{P}_{kj} \right) \left(\frac{1}{\omega_i + \omega_j + \omega_k} - \frac{\mathrm{S}}{\omega_i - \omega_j - \omega_k} \right) + \frac{\mathrm{f}_{jkk}}{\omega_j} \left[2\mathrm{S}\boldsymbol{P}_{ji} + (1+\mathrm{S})\boldsymbol{P}_{ij} \right] \right\} \\
&+ \frac{s_0}{2} \sum_{j=1}^{N} \sum_{k=1}^{N} \left(\sum_{\tau} B_{\tau}^{\mathrm{eq}} \zeta_{ik,\tau} \zeta_{jk,\tau} \right) \boldsymbol{P}_j \left\{ \frac{\sqrt{\omega_i \omega_j}}{\omega_k} \left(\frac{1}{\omega_i + \omega_j} + \frac{\mathrm{S}(1-\delta_{ij})}{\omega_i - \omega_j} \right) - \frac{\omega_k}{\sqrt{\omega_i \omega_j}} \left(\frac{1}{\omega_i + \omega_j} - \frac{\mathrm{S}(1-\delta_{ij})}{\omega_i - \omega_j} \right) \right\} \\
&+ \frac{s_0}{16} \sum_{j=1}^{N} \sum_{k=1}^{N} \sum_{l=1}^{N} \mathrm{f}_{ikl} \mathrm{f}_{jkl} \boldsymbol{P}_j \left\{ (1-\delta_{ij})(1-\delta_{ik})(1-\delta_{il}) \left[\frac{1}{(\omega_i + \omega_j)(\omega_i + \omega_k + \omega_l)} \right. \right. \\
&\quad \left. - \frac{1}{(\omega_i + \omega_j)(\omega_i - \omega_k - \omega_l)} - \frac{\mathrm{S}}{(\omega_i - \omega_j)(\omega_i + \omega_k + \omega_l)} + \frac{\mathrm{S}}{(\omega_i - \omega_j)(\omega_i - \omega_k - \omega_l)} \right] \\
&\quad + \delta_{ij}(1+\delta_{ik})(1-\delta_{il}) \left[\frac{1}{2\omega_i(\omega_i + \omega_k + \omega_l)} - \frac{1}{2\omega_i(\omega_i - \omega_k - \omega_l)} + \frac{\mathrm{S}}{2(\omega_i + \omega_k + \omega_l)^2} - \frac{\mathrm{S}}{2(\omega_i - \omega_k - \omega_l)^2} \right] \\
&\quad \left. + (1-\delta_{ij})(1-\delta_{ik})\delta_{il} \left[\frac{4}{\omega_k(\omega_i + \omega_j)} + \frac{2}{(\omega_i + \omega_j)(2\omega_i + \omega_k)} - \frac{4\mathrm{S}}{\omega_k(\omega_i - \omega_j)} - \frac{2\mathrm{S}}{(\omega_i - \omega_j)(2\omega_i + \omega_k)} \right] \right\} \\
&+ \mathrm{f}_{ijk} \mathrm{f}_{llk} \boldsymbol{P}_j \left\{ \frac{\delta_{ij}}{\omega_i \omega_k} \left(1 + \frac{\delta_{ik}\delta_{il}(6-4\mathrm{S})}{9} \right) \right. \\
&\quad + (1-\delta_{ij})(1-\delta_{ik})(1-\delta_{il}) \left[\frac{2}{\omega_k(\omega_i + \omega_j)} - \frac{2\mathrm{S}}{\omega_k(\omega_i - \omega_j)} \right] \\
&\quad \left. + \delta_{ik}(1-\delta_{ij})(1 + \frac{2\delta_{il}}{3}) \left[\frac{2}{\omega_i(\omega_i + \omega_j)} - \frac{2\mathrm{S}}{\omega_i(\omega_i - \omega_j)} \right] \right\}
\end{aligned}
$$

S6.1.2 2-quanta transitions: first overtones and combinations

$$
\langle \boldsymbol{P} \rangle_{0,(1+\delta_{ij})_i(1-\delta_{ij})_j} = \sqrt{\frac{2}{1+\delta_{ij}}} \times \left[\frac{s_1 \times S}{2} \left(\boldsymbol{P}_{ij} + \boldsymbol{P}_{ji} \right) + \frac{s_0}{4} \sum_{k=1}^{N} \mathrm{f}_{ijk} \boldsymbol{P}_k \left(\frac{\mathrm{S}}{\omega_i + \omega_j - \omega_k} - \frac{1}{\omega_i + \omega_j + \omega_k} \right) \right]
$$

S6.1.3 3-quanta transitions: second overtones and combinations

$$
\begin{aligned}
&\langle \boldsymbol{P} \rangle_{0,(1+\delta_{ij}+\delta_{ik})_i(1-\delta_{ij})_j(1-\delta_{ik})_k} = \\
&\quad s_2(\boldsymbol{P}_{ijk} + \boldsymbol{P}_{ikj} + \boldsymbol{P}_{jki})\mathrm{S} \\
&\quad + A \sum_{l=1}^{N} \Bigg\{ \frac{s_0}{4} \mathrm{f}_{ijkl} \boldsymbol{P}_l \left[\frac{\mathrm{S}}{\omega_i + \omega_j + \omega_k - \omega_l} - \frac{1}{\omega_i + \omega_j + \omega_k + \omega_l} \right] \\
&\qquad + \frac{s_0}{2} \sum_{\tau=x,y,z} B_\tau^{\mathrm{eq}} \boldsymbol{P}_l \Bigg\{ \Bigg[\zeta_{ij,\tau}\zeta_{kl,\tau} \left(\sqrt{\frac{\omega_j\omega_l}{\omega_i\omega_k}} - \sqrt{\frac{\omega_i\omega_l}{\omega_j\omega_k}} \right) + \zeta_{ik,\tau}\zeta_{jl,\tau} \left(\sqrt{\frac{\omega_k\omega_l}{\omega_i\omega_j}} - \sqrt{\frac{\omega_i\omega_l}{\omega_j\omega_k}} \right) \\
&\qquad\quad + \zeta_{il,\tau}\zeta_{jk,\tau} \left(\sqrt{\frac{\omega_k\omega_l}{\omega_i\omega_j}} - \sqrt{\frac{\omega_j\omega_l}{\omega_i\omega_k}} \right) \Bigg] \times \left[\frac{1}{\omega_i + \omega_j + \omega_k + \omega_l} + \frac{\mathrm{S}}{\omega_i + \omega_j + \omega_k - \omega_l} \right] \\
&\qquad + \Bigg[\zeta_{ij,\tau}\zeta_{kl,\tau} \left(\sqrt{\frac{\omega_i\omega_k}{\omega_j\omega_l}} - \sqrt{\frac{\omega_j\omega_k}{\omega_i\omega_l}} \right) + \zeta_{ik,\tau}\zeta_{jl,\tau} \left(\sqrt{\frac{\omega_i\omega_j}{\omega_k\omega_l}} - \sqrt{\frac{\omega_j\omega_k}{\omega_i\omega_l}} \right) \\
&\qquad\quad + \zeta_{il,\tau}\zeta_{jk,\tau} \left(\sqrt{\frac{\omega_i\omega_j}{\omega_k\omega_l}} - \sqrt{\frac{\omega_i\omega_k}{\omega_j\omega_l}} \right) \Bigg] \times \left[\frac{1}{\omega_i + \omega_j + \omega_k + \omega_l} - \frac{\mathrm{S}}{\omega_i + \omega_j + \omega_k - \omega_l} \right] \Bigg\} \\
&\qquad + \frac{s_1}{4} \Bigg\{ \mathrm{f}_{ijl} \left[\frac{\mathrm{S}(\boldsymbol{P}_{kl} + \boldsymbol{P}_{lk})}{\omega_i + \omega_j - \omega_l} - \frac{\boldsymbol{P}_{kl} + \mathrm{S}\boldsymbol{P}_{lk}}{\omega_i + \omega_j + \omega_l} \right] + \mathrm{f}_{ikl} \left[\frac{\mathrm{S}(\boldsymbol{P}_{jl} + \boldsymbol{P}_{lj})}{\omega_i + \omega_k - \omega_l} - \frac{\boldsymbol{P}_{jl} + \mathrm{S}\boldsymbol{P}_{lj}}{\omega_i + \omega_k + \omega_l} \right] \\
&\qquad\quad + \mathrm{f}_{jkl} \left[\frac{\mathrm{S}(\boldsymbol{P}_{il} + \boldsymbol{P}_{li})}{\omega_j + \omega_k - \omega_l} - \frac{\boldsymbol{P}_{il} + \mathrm{S}\boldsymbol{P}_{li}}{\omega_j + \omega_k + \omega_l} \right] \Bigg\} \\
&\qquad + \frac{s_0}{8} \sum_{m=1}^{N} \mathrm{f}_{ijm}\mathrm{f}_{klm} \boldsymbol{P}_l \Bigg[\frac{1}{(\omega_m + \omega_j + \omega_i)(\omega_l + \omega_k + \omega_j + \omega_i)} + \frac{1}{(\omega_m - \omega_j - \omega_i)(\omega_l + \omega_k + \omega_j + \omega_i)} \\
&\qquad\quad + \frac{\mathrm{S}}{(\omega_m + \omega_j + \omega_i)(\omega_l - \omega_k - \omega_j - \omega_i)} + \frac{\mathrm{S}}{(\omega_m - \omega_j - \omega_i)(\omega_l - \omega_k - \omega_j - \omega_i)} \Bigg] \\
&\qquad + \mathrm{f}_{ikm}\mathrm{f}_{jlm} \boldsymbol{P}_l \Bigg[\frac{1}{(\omega_m + \omega_k + \omega_i)(\omega_l + \omega_k + \omega_j + \omega_i)} + \frac{1}{(\omega_m - \omega_k - \omega_i)(\omega_l + \omega_k + \omega_j + \omega_i)} \\
&\qquad\quad + \frac{\mathrm{S}}{(\omega_m + \omega_k + \omega_i)(\omega_l - \omega_k - \omega_j - \omega_i)} + \frac{\mathrm{S}}{(\omega_m - \omega_k - \omega_i)(\omega_l - \omega_k - \omega_j - \omega_i)} \Bigg] \\
&\qquad + \mathrm{f}_{jkm}\mathrm{f}_{ilm} \boldsymbol{P}_l \Bigg[\frac{1}{(\omega_m + \omega_k + \omega_j)(\omega_l + \omega_k + \omega_j + \omega_i)} + \frac{1}{(\omega_m - \omega_k - \omega_j)(\omega_l + \omega_k + \omega_j + \omega_i)} \\
&\qquad\quad + \frac{\mathrm{S}}{(\omega_m + \omega_k + \omega_j)(\omega_l - \omega_k - \omega_j - \omega_i)} + \frac{\mathrm{S}}{(\omega_m - \omega_k - \omega_j)(\omega_l - \omega_k - \omega_j - \omega_i)} \Bigg] \Bigg\}
\end{aligned}
$$

with

$$
A = \frac{\sqrt{1 + \delta_{ij} + \delta_{ik}}}{(1 + \delta_{ij} + \delta_{ik})!}
$$

and

$$\langle \boldsymbol{P} \rangle_{(1+\delta_{ij}+\delta_{ik})_i(1-\delta_{ij})_j(1-\delta_{ik})_k,0} = \mathrm{S} \times \langle \boldsymbol{P} \rangle_{0,(1+\delta_{ij}+\delta_{ik})_i(1-\delta_{ij})_j(1-\delta_{ik})_k}$$

In the original paper,[S13] an exponent was missing, leading to a wrong account of the Coriolis couplings, which was however correctly implemented. The equations above are exactly equivalent to those published in Ref.[S14] The transformation between the equation above and Eq. S5 in Ref.[S14] is straightforward. By introducing the following identity

$$\frac{1}{\omega_i + \omega_j + \omega_k + \omega_l} - \frac{1}{\omega_i + \omega_j + \omega_k - \omega_l} = C_{ijkl} \left(\frac{1}{\omega_i + \omega_j + \omega_k + \omega_l} + \frac{1}{\omega_i + \omega_j + \omega_k - \omega_l} \right)$$

where

$$C_{ijkl} = \frac{\dfrac{1}{\omega_i + \omega_j + \omega_k + \omega_l} - \dfrac{1}{\omega_i + \omega_j + \omega_k - \omega_l}}{\dfrac{1}{\omega_i + \omega_j + \omega_k + \omega_l} + \dfrac{1}{\omega_i + \omega_j + \omega_k - \omega_l}} = -\frac{\omega_l}{\omega_i + \omega_j + \omega_k}$$

in the expression of $\langle \boldsymbol{P} \rangle_{0,(1+\delta_{ij}+\delta_{ik})_i(1-\delta_{ij})_j(1-\delta_{ik})_k}$ reported above and considering the case $S = 1$, an expression identical to Eq. S5 of Ref.[S14] is obtained.

S6.2 Resonant cases

S6.2.1 1-quanta transitions: fundamentals

$$
\begin{aligned}
\langle \boldsymbol{P} \rangle_{0,1_i} &= s_0 \times \mathrm{S} \times \boldsymbol{P}_i + \frac{s_2}{2} \sum_{j=1}^{N} \{ \boldsymbol{P}_{jij} + \boldsymbol{P}_{ijj} + \mathrm{S} \boldsymbol{P}_{jji} \} - \frac{s_0}{8} \sum_{j=1}^{N} \sum_{k=1}^{N} \mathrm{f}_{ijkk} \boldsymbol{P}_j \left[\frac{1}{\omega_i + \omega_j} \right] \\
&- \frac{s_1}{8} \sum_{j=1}^{N} \sum_{k=1}^{N} \left\{ \mathrm{f}_{ijk} \left(\boldsymbol{P}_{jk} + \boldsymbol{P}_{kj} \right) \left(\frac{1}{\omega_i + \omega_j + \omega_k} - \frac{\mathrm{S}}{\omega_i - \omega_j - \omega_k} \right) + \frac{\mathrm{f}_{jkk}}{\omega_j} \left[2\mathrm{S} \boldsymbol{P}_{ji} + (1 + \mathrm{S}) \boldsymbol{P}_{ij} \right] \right\} \\
&+ \frac{s_0}{2} \sum_{j=1}^{N} \sum_{k=1}^{N} \left(\sum_{\tau} B_{\tau}^{\mathrm{eq}} \zeta_{ik,\tau} \zeta_{jk,\tau} \right) \boldsymbol{P}_j \left\{ \frac{\sqrt{\omega_i \omega_j}}{\omega_k} \left(\frac{1}{\omega_i + \omega_j} \right) - \frac{\omega_k}{\sqrt{\omega_i \omega_j}} \left(\frac{1}{\omega_i + \omega_j} \right) \right\} \\
&+ \frac{s_0}{16} \sum_{j=1}^{N} \sum_{k=1}^{N} \sum_{l=1}^{N} \mathrm{f}_{ikl} \mathrm{f}_{jkl} \boldsymbol{P}_j \left\{ (1 - \delta_{ij})(1 - \delta_{ik})(1 - \delta_{il}) \left[\frac{1}{(\omega_i + \omega_j)(\omega_i + \omega_k + \omega_l)} \right. \right. \\
&\quad \left. - \frac{1}{(\omega_i + \omega_j)(\omega_i - \omega_k - \omega_l)} + \frac{\mathrm{S}}{(\omega_i + \omega_k + \omega_l)(\omega_j + \omega_k + \omega_l)} \right] \\
&\quad + \delta_{ij}(1 + \delta_{ik})(1 - \delta_{il}) \left[\frac{1}{2\omega_i(\omega_i + \omega_k + \omega_l)} - \frac{1}{2\omega_i(\omega_i - \omega_k - \omega_l)} + \frac{\mathrm{S}}{2(\omega_i + \omega_k + \omega_l)^2} - \frac{\mathrm{S}}{2(\omega_i - \omega_k - \omega_l)^2} \right] \\
&\quad + (1 - \delta_{ij})(1 - \delta_{ik}) \delta_{il} \left[\frac{4}{\omega_k(\omega_i + \omega_j)} + \frac{2}{(\omega_i + \omega_j)(2\omega_i + \omega_k)} - \frac{\mathrm{S}}{\omega_k(\omega_i - \omega_j - \omega_k)} \right. \\
&\quad \left. \left. + \frac{2\mathrm{S}}{(\omega_i + \omega_j + \omega_k)(2\omega_i + \omega_k)} \right] \right\} \\
&+ \mathrm{f}_{ijk} \mathrm{f}_{llk} \boldsymbol{P}_j \left\{ \frac{\delta_{ij}}{\omega_i \omega_k} \left(1 + \frac{\delta_{ik} \delta_{il} (6 - 4\mathrm{S})}{9} \right) \right. \\
&\quad + (1 - \delta_{ij})(1 - \delta_{ik})(1 - \delta_{il}) \left[\frac{2}{\omega_k(\omega_i + \omega_j)} - \frac{\mathrm{S}}{\omega_k(\omega_i - \omega_j - \omega_k)} \right] \\
&\quad \left. + \delta_{ik}(1 - \delta_{ij}) \left[\frac{2(1 + 2\delta_{il}/3)}{\omega_i(\omega_i + \omega_j)} + \frac{3\mathrm{S}}{\omega_i \omega_j} + \frac{\delta_{il} \mathrm{S}}{3\omega_i(2\omega_i + \omega_j)} \right] \right\}
\end{aligned}
$$

S6.2.2 2-quanta transitions: first overtones and combinations

There is no problem of resonances with intermediate states, the equations are the same as for the non-resonant case.

S6.2.3 3-quanta transitions: second overtones and combinations

$$
\begin{aligned}
&\langle \boldsymbol{P} \rangle_{0,(1+\delta_{ij}+\delta_{ik})_i(1-\delta_{ij})_j(1-\delta_{ik})_k} = \\
&\quad s_2(\boldsymbol{P}_{ijk} + \boldsymbol{P}_{ikj} + \boldsymbol{P}_{jki})\mathrm{S} \\
&\quad + A\sum_{l=1}^{N}\Bigg\{ \frac{s_0}{4}\mathrm{f}_{ijkl}\boldsymbol{P}_l\left[-\frac{1}{\omega_i+\omega_j+\omega_k+\omega_l}\right] \\
&\qquad + \frac{s_0}{2}\sum_{\tau=x,y,z} B_\tau^{\mathrm{eq}}\boldsymbol{P}_l\Bigg\{\Bigg[\zeta_{ij,\tau}\zeta_{kl,\tau}\left(\sqrt{\frac{\omega_j\omega_l}{\omega_i\omega_k}}+\sqrt{\frac{\omega_i\omega_k}{\omega_j\omega_l}}-\sqrt{\frac{\omega_i\omega_l}{\omega_j\omega_k}}-\sqrt{\frac{\omega_j\omega_k}{\omega_i\omega_l}}\right) \\
&\qquad\quad + \zeta_{ik,\tau}\zeta_{jl,\tau}\left(\sqrt{\frac{\omega_k\omega_l}{\omega_i\omega_j}}+\sqrt{\frac{\omega_i\omega_j}{\omega_k\omega_l}}-\sqrt{\frac{\omega_i\omega_l}{\omega_j\omega_k}}-\sqrt{\frac{\omega_j\omega_k}{\omega_i\omega_l}}\right) \\
&\qquad\quad + \zeta_{il,\tau}\zeta_{jk,\tau}\left(\sqrt{\frac{\omega_k\omega_l}{\omega_i\omega_j}}+\sqrt{\frac{\omega_i\omega_j}{\omega_k\omega_l}}-\sqrt{\frac{\omega_j\omega_l}{\omega_i\omega_k}}-\sqrt{\frac{\omega_i\omega_k}{\omega_j\omega_l}}\right)\Bigg]\times\frac{1}{\omega_i+\omega_j+\omega_k+\omega_l}\Bigg\} \\
&\qquad + \frac{s_1}{4}\Bigg\{\mathrm{f}_{ijl}\left[\frac{\mathrm{S}(\boldsymbol{P}_{kl}+\boldsymbol{P}_{lk})}{\omega_i+\omega_j-\omega_l}-\frac{\boldsymbol{P}_{kl}+\mathrm{S}\boldsymbol{P}_{lk}}{\omega_i+\omega_j+\omega_l}\right]+\mathrm{f}_{ikl}\left[\frac{\mathrm{S}(\boldsymbol{P}_{jl}+\boldsymbol{P}_{lj})}{\omega_i+\omega_k-\omega_l}-\frac{\boldsymbol{P}_{jl}+\mathrm{S}\boldsymbol{P}_{lj}}{\omega_i+\omega_k+\omega_l}\right] \\
&\qquad\quad + \mathrm{f}_{jkl}\left[\frac{\mathrm{S}(\boldsymbol{P}_{il}+\boldsymbol{P}_{li})}{\omega_j+\omega_k-\omega_l}-\frac{\boldsymbol{P}_{il}+\mathrm{S}\boldsymbol{P}_{li}}{\omega_j+\omega_k+\omega_l}\right]\Bigg\} \\
&\qquad + \frac{s_0}{8}\sum_{m=1}^{N}\mathrm{f}_{ijm}\mathrm{f}_{klm}\boldsymbol{P}_l\Bigg[\frac{1}{(\omega_m+\omega_j+\omega_i)(\omega_l+\omega_k+\omega_j+\omega_i)}+\frac{1}{(\omega_m-\omega_j-\omega_i)(\omega_l+\omega_k+\omega_j+\omega_i)} \\
&\qquad\quad - \frac{\mathrm{S}}{(\omega_m+\omega_j+\omega_i)(\omega_k-\omega_l-\omega_m)}\Bigg] \\
&\qquad\quad + \mathrm{f}_{ikm}\mathrm{f}_{jlm}\boldsymbol{P}_l\Bigg[\frac{1}{(\omega_m+\omega_k+\omega_i)(\omega_l+\omega_k+\omega_j+\omega_i)}+\frac{1}{(\omega_m-\omega_k-\omega_i)(\omega_l+\omega_k+\omega_j+\omega_i)} \\
&\qquad\quad - \frac{\mathrm{S}}{(\omega_m+\omega_k+\omega_i)(\omega_j-\omega_l-\omega_m)}\Bigg] \\
&\qquad\quad + \mathrm{f}_{jkm}\mathrm{f}_{ilm}\boldsymbol{P}_l\Bigg[\frac{1}{(\omega_m+\omega_k+\omega_j)(\omega_l+\omega_k+\omega_j+\omega_i)}+\frac{1}{(\omega_m-\omega_k-\omega_j)(\omega_l+\omega_k+\omega_j+\omega_i)} \\
&\qquad\quad - \frac{\mathrm{S}}{(\omega_m+\omega_k+\omega_j)(\omega_i-\omega_l-\omega_m)}\Bigg] \\
&\qquad\quad + \mathrm{f}_{ijl}\mathrm{f}_{kmm}\boldsymbol{P}_l\left[\frac{\mathrm{S}}{2\omega_k(\omega_l-\omega_i-\omega_j)}\right]+\mathrm{f}_{ikl}\mathrm{f}_{jmm}\boldsymbol{P}_l\left[\frac{\mathrm{S}}{2\omega_j(\omega_l-\omega_i-\omega_k)}\right] \\
&\qquad\quad + \mathrm{f}_{jkl}\mathrm{f}_{imm}\boldsymbol{P}_l\left[\frac{\mathrm{S}}{2\omega_i(\omega_l-\omega_j-\omega_k)}\right]+\mathrm{f}_{ijk}\mathrm{f}_{lmm}\boldsymbol{P}_l\left[\frac{\mathrm{S}}{2\omega_l(\omega_i+\omega_j+\omega_k)}\right]\Bigg\}
\end{aligned}
$$

S7 States involving both doubly- and triply-degenerate vibrations

S7.1 Fundamental band of a triply-degenerate mode

The linear transformation connecting the three fundamental bands of a triply-degenerate mode s' is defined by the following expression:

$$\begin{pmatrix} |\psi^S_{1_{s'},1_{s'},+1_{s'}}\rangle \\ |\psi^S_{1_{s'},1_{s'},0_{s'}}\rangle \\ |\psi^S_{1_{s'},1_{s'},-1_{s'}}\rangle \end{pmatrix} = \begin{pmatrix} \frac{1}{\sqrt{2}} & \frac{i}{\sqrt{2}} & 0 \\ 0 & 0 & 1 \\ \frac{1}{\sqrt{2}} & -\frac{i}{\sqrt{2}} & 0 \end{pmatrix} \begin{pmatrix} |\psi^C_{1_{s'_1}}\rangle \\ |\psi^C_{1_{s'_2}}\rangle \\ |\psi^C_{1_{s'_3}}\rangle \end{pmatrix} \tag{42}$$

S7.2 First overtones of triply-degenerate vibrations

Let us consider an overtone of a triply-degenerate vibration, namely s'. The 6 states corresponding to the first overtone of s' can be obtained through the following expression,

$$\begin{pmatrix} |\psi^S_{2_{s'},2_{s'},+2_{s'}}\rangle \\ |\psi^S_{2_{s'},2_{s'},+1_{s'}}\rangle \\ |\psi^S_{2_{s'},2_{s'},0_{s'}}\rangle \\ |\psi^S_{2_{s'},0_{s'},0_{s'}}\rangle \\ |\psi^S_{2_{s'},2_{s'},-1_{s'}}\rangle \\ |\psi^S_{2_{s'},2_{s'},-2_{s'}}\rangle \end{pmatrix} = \begin{pmatrix} \frac{1}{2} & -\frac{1}{2} & 0 & \frac{i}{\sqrt{2}} & 0 & 0 \\ 0 & 0 & 0 & 0 & -\frac{1}{\sqrt{2}} & -\frac{i}{\sqrt{2}} \\ -\frac{1}{\sqrt{6}} & -\frac{1}{\sqrt{6}} & \frac{2}{\sqrt{6}} & 0 & 0 & 0 \\ -\frac{1}{\sqrt{3}} & -\frac{1}{\sqrt{3}} & -\frac{1}{\sqrt{3}} & 0 & 0 & 0 \\ 0 & 0 & 0 & 0 & -\frac{1}{\sqrt{2}} & \frac{i}{\sqrt{2}} \\ \frac{1}{2} & -\frac{1}{2} & 0 & -\frac{i}{\sqrt{2}} & 0 & 0 \end{pmatrix} \begin{pmatrix} |\psi^C_{2_{s'_1}}\rangle \\ |\psi^C_{2_{s'_2}}\rangle \\ |\psi^C_{2_{s'_3}}\rangle \\ |\psi^C_{1_{s'_1}1_{s'_2}}\rangle \\ |\psi^C_{1_{s'_1}1_{s'_3}}\rangle \\ |\psi^C_{1_{s'_2}1_{s'_3}}\rangle \end{pmatrix} \tag{43}$$

S7.2.1 Anharmonic energies

$$\begin{aligned}
\varepsilon^S_{2_{s'},2_{s'},\pm 2_{s'}} &= \frac{1}{4}\varepsilon^C_{2_{s'_1}} + \frac{1}{4}\varepsilon^C_{2_{s'_2}} + \frac{1}{2}\varepsilon^C_{1_{s'_1}1_{s'_2}} - \frac{1}{2}\langle\psi^C_{2_{s'_1}}|\tilde{\mathcal{H}}|\psi^C_{2_{s'_2}}\rangle \\
\varepsilon^S_{2_{s'},2_{s'},\pm 1_{s'}} &= \frac{1}{2}\varepsilon^C_{1_{s'_1}1_{s'_3}} + \frac{1}{2}\varepsilon^C_{1_{s'_2}1_{s'_3}} \\
\varepsilon^S_{2_{s'},2_{s'},0_{s'}} &= \frac{1}{6}\varepsilon^C_{2_{s'_1}} + \frac{1}{6}\varepsilon^C_{2_{s'_2}} + \frac{2}{3}\varepsilon^C_{2_{s'_3}} + \frac{1}{3}\langle\psi^C_{2_{s'_1}}|\tilde{\mathcal{H}}|\psi^C_{2_{s'_2}}\rangle - \frac{2}{3}\langle\psi^C_{2_{s'_1}}|\tilde{\mathcal{H}}|\psi^C_{2_{s'_3}}\rangle - \frac{2}{3}\langle\psi^C_{2_{s'_2}}|\tilde{\mathcal{H}}|\psi^C_{2_{s'_3}}\rangle \\
\varepsilon^S_{2_{s'},0_{s'},0_{s'}} &= \frac{1}{3}\varepsilon^C_{2_{s'_1}} + \frac{1}{3}\varepsilon^C_{2_{s'_2}} + \frac{1}{3}\varepsilon^C_{2_{s'_3}} + \frac{2}{3}\langle\psi^C_{2_{s'_1}}|\tilde{\mathcal{H}}|\psi^C_{2_{s'_2}}\rangle + \frac{2}{3}\langle\psi^C_{2_{s'_1}}|\tilde{\mathcal{H}}|\psi^C_{2_{s'_3}}\rangle + \frac{2}{3}\langle\psi^C_{2_{s'_2}}|\tilde{\mathcal{H}}|\psi^C_{2_{s'_3}}\rangle
\end{aligned} \tag{44}$$

S7.2.2 Anharmonic dipole strengths

$$\begin{aligned}
D^S_{2_{s'},2_{s'},\pm 2_{s'}} &= \frac{1}{4}D^C_{2_{s'_1}} + \frac{1}{4}D^C_{2_{s'_2}} + \frac{1}{2}D^C_{1_{s'_1}1_{s'_2}} - \frac{1}{2}\langle\boldsymbol{\mu}\rangle^C_{2_{s'_1}}\langle\boldsymbol{\mu}\rangle^C_{2_{s'_2}} \\
D^S_{2_{s'},2_{s'},\pm 1_{s'}} &= \frac{1}{2}D^C_{1_{s'_1}1_{s'_3}} + \frac{1}{2}D^C_{1_{s'_2}1_{s'_3}} \\
D^S_{2_{s'},2_{s'},0_{s'}} &= \frac{1}{6}D^C_{2_{s'_1}} + \frac{1}{6}D^C_{2_{s'_2}} + \frac{2}{3}D^C_{2_{s'_3}} + \frac{1}{3}\langle\boldsymbol{\mu}\rangle^C_{2_{s'_1}}\langle\boldsymbol{\mu}\rangle^C_{2_{s'_2}} - \frac{2}{3}\langle\boldsymbol{\mu}\rangle^C_{2_{s'_1}}\langle\boldsymbol{\mu}\rangle^C_{2_{s'_3}} - \frac{2}{3}\langle\boldsymbol{\mu}\rangle^C_{2_{s'_2}}\langle\boldsymbol{\mu}\rangle^C_{2_{s'_3}} \\
D^S_{2_{s'},0_{s'},0_{s'}} &= \frac{1}{3}D^C_{2_{s'_1}} + \frac{1}{3}D^C_{2_{s'_2}} + \frac{1}{3}D^C_{2_{s'_3}} + \frac{2}{3}\langle\boldsymbol{\mu}\rangle^C_{2_{s'_1}}\langle\boldsymbol{\mu}\rangle^C_{2_{s'_2}} + \frac{2}{3}\langle\boldsymbol{\mu}\rangle^C_{2_{s'_1}}\langle\boldsymbol{\mu}\rangle^C_{2_{s'_3}} + \frac{2}{3}\langle\boldsymbol{\mu}\rangle^C_{2_{s'_2}}\langle\boldsymbol{\mu}\rangle^C_{2_{s'_3}}
\end{aligned} \tag{45}$$

S7.2.3 Anharmonic Raman activities

$$\begin{aligned}
S^S_{2_{s'},2_{s'},\pm 2_{s'}} &= \frac{1}{4}S^C_{2_{s'_1}} + \frac{1}{4}S^C_{2_{s'_2}} + \frac{1}{2}S^C_{1_{s'_1}1_{s'_2}} - \frac{1}{2}S^C_{2_{s'_1};2_{s'_2}} \\
S^S_{2_{s'},2_{s'},\pm 1_{s'}} &= \frac{1}{2}S^C_{1_{s'_1}1_{s'_3}} + \frac{1}{2}S^C_{1_{s'_2}1_{s'_3}} \\
S^S_{2_{s'},2_{s'},0_{s'}} &= \frac{1}{6}S^C_{2_{s'_1}} + \frac{1}{6}S^C_{2_{s'_2}} + \frac{2}{3}S^C_{2_{s'_3}} + \frac{1}{3}S^C_{2_{s'_1};2_{s'_2}} - \frac{2}{3}S^C_{2_{s'_1};2_{s'_3}} - \frac{2}{3}S^C_{2_{s'_2};2_{s'_3}} \\
S^S_{2_{s'},0_{s'},0_{s'}} &= \frac{1}{3}S^C_{2_{s'_1}} + \frac{1}{3}S^C_{2_{s'_2}} + \frac{1}{3}S^C_{2_{s'_3}} + \frac{2}{3}S^C_{2_{s'_1};2_{s'_2}} + \frac{2}{3}S^C_{2_{s'_1};2_{s'_3}} + \frac{2}{3}S^C_{2_{s'_2};2_{s'_3}}
\end{aligned} \tag{46}$$

S7.3 Binary combinations between triply-degenerate vibrations

Let us consider a binary combination band involving two triply-degenerate vibrations, namely s' and t'. The 9 states corresponding to the binary combination band between s' and t' can be obtained by performing one-by-one products of the matrices $\mathbf{Q}_{1_s}$ and $\mathbf{Q}_{1_t}$, which for instance, have the same expression (let us stress that the form of the rotation is only affected by the type of band, and not by the vibrational mode itself). As a matter of fact, the form of the

wave functions $|\psi^S_{1_{s'}1_{t'},1_{s'},1_{t'},m_{s'}m_{t'}}\rangle$ (with $m_{s'}, m_{t'} = -1, 0, +1$) is:

$$\begin{pmatrix} |\psi^S_{1_{s'}1_{t'},1_{s'}1_{t'},+1_{s'}+1_{t'}}\rangle \\ |\psi^S_{1_{s'}1_{t'},1_{s'}1_{t'},-1_{s'}-1_{t'}}\rangle \\ |\psi^S_{1_{s'}1_{t'},1_{s'}1_{t'},+1_{s'}-1_{t'}}\rangle \\ |\psi^S_{1_{s'}1_{t'},1_{s'}1_{t'},-1_{s'}+1_{t'}}\rangle \\ |\psi^S_{1_{s'}1_{t'},1_{s'}1_{t'},+1_{s'}0_{t'}}\rangle \\ |\psi^S_{1_{s'}1_{t'},1_{s'}1_{t'},-1_{s'}0_{t'}}\rangle \\ |\psi^S_{1_{s'}1_{t'},1_{s'}1_{t'},0_{s'}+1_{t'}}\rangle \\ |\psi^S_{1_{s'}1_{t'},1_{s'}1_{t'},0_{s'}-1_{t'}}\rangle \\ |\psi^S_{1_{s'}1_{t'},1_{s'}1_{t'},0_{s'}0_{t'}}\rangle \end{pmatrix} = \frac{1}{2} \begin{pmatrix} 1 & -1 & i & i & 0 & 0 & 0 & 0 & 0 \\ 1 & -1 & -i & -i & 0 & 0 & 0 & 0 & 0 \\ 1 & 1 & -i & i & 0 & 0 & 0 & 0 & 0 \\ 1 & 1 & i & -i & 0 & 0 & 0 & 0 & 0 \\ 0 & 0 & 0 & 0 & \sqrt{2} & 0 & -i\sqrt{2} & 0 & 0 \\ 0 & 0 & 0 & 0 & \sqrt{2} & 0 & i\sqrt{2} & 0 & 0 \\ 0 & 0 & 0 & 0 & 0 & \sqrt{2} & 0 & i\sqrt{2} & 0 \\ 0 & 0 & 0 & 0 & 0 & \sqrt{2} & 0 & -i\sqrt{2} & 0 \\ 0 & 0 & 0 & 0 & 0 & 0 & 0 & 0 & 2 \end{pmatrix} \begin{pmatrix} |\psi^C_{1_{s'_1}1_{t'_1}}\rangle \\ |\psi^C_{1_{s'_2}1_{t'_2}}\rangle \\ |\psi^C_{1_{s'_1}1_{t'_2}}\rangle \\ |\psi^C_{1_{s'_2}1_{t'_1}}\rangle \\ |\psi^C_{1_{s'_1}1_{t'_3}}\rangle \\ |\psi^C_{1_{s'_3}1_{t'_1}}\rangle \\ |\psi^C_{1_{s'_2}1_{t'_3}}\rangle \\ |\psi^C_{1_{s'_3}1_{t'_2}}\rangle \\ |\psi^C_{1_{s'_3}1_{t'_3}}\rangle \end{pmatrix} \tag{47}$$

S7.3.1 Anharmonic energies

$$\begin{aligned} \varepsilon^S_{1_{s'}1_{t'},1_{s'}1_{t'},\pm1_{s'}\pm1_{t'}} &= \frac{1}{4}\left[\varepsilon_{s't'} - 2\langle\psi^C_{1_{s'_1}1_{t'_1}}|\tilde{\mathcal{H}}|\psi^C_{1_{s'_2}1_{t'_2}}\rangle + 2\langle\psi^C_{1_{s'_1}1_{t'_2}}|\tilde{\mathcal{H}}|\psi^C_{1_{s'_2}1_{t'_1}}\rangle\right] \\ \varepsilon^S_{1_{s'}1_{t'},1_{s'}1_{t'},\pm1_{s'}\mp1_{t'}} &= \frac{1}{4}\left[\varepsilon_{s't'} + 2\langle\psi^C_{1_{s'_1}1_{t'_1}}|\tilde{\mathcal{H}}|\psi^C_{1_{s'_2}1_{t'_2}}\rangle - 2\langle\psi^C_{1_{s'_1}1_{t'_2}}|\tilde{\mathcal{H}}|\psi^C_{1_{s'_2}1_{t'_1}}\rangle\right] \\ \varepsilon^S_{1_{s'}1_{t'},1_{s'}1_{t'},\pm1_{s'}0_{t'}} &= \frac{1}{2}\left[\varepsilon^C_{1_{s'_1}1_{t'_3}} + \varepsilon^C_{1_{s'_2}1_{t'_3}}\right] \\ \varepsilon^S_{1_{s'}1_{t'},1_{s'}1_{t'},0_{s'}\pm1_{t'}} &= \frac{1}{2}\left[\varepsilon^C_{1_{s'_3}1_{t'_1}} + \varepsilon^C_{1_{s'_3}1_{t'_2}}\right] \\ \varepsilon^S_{1_{s'}1_{t'},1_{s'}1_{t'},0_{s'}0_{t'}} &= \varepsilon^C_{1_{s'_3}1_{t'_3}} \end{aligned} \tag{48}$$

where

$$\varepsilon_{s't'} = \varepsilon^C_{1_{s'_1}1_{t'_1}} + \varepsilon^C_{1_{s'_2}1_{t'_2}} + \varepsilon^C_{1_{s'_1}1_{t'_2}} + \varepsilon^C_{1_{s'_2}1_{t'_1}} \tag{49}$$

S7.3.2 Anharmonic dipole strengths

$$\begin{aligned}
D^S_{1_{s'}1_{t'},1_{s'}1_{t'},\pm 1_{s'}\pm 1_{t'}} &= \frac{1}{4}\left[D_{s't'} - 2\langle\boldsymbol{\mu}\rangle^C_{1_{s'_1}1_{t'_1}}\langle\boldsymbol{\mu}\rangle^C_{1_{s'_2}1_{t'_2}} + 2\langle\boldsymbol{\mu}\rangle^C_{1_{s'_1}1_{t'_2}}\langle\boldsymbol{\mu}\rangle^C_{1_{s'_2}1_{t'_1}}\right] \\
D^S_{1_{s'}1_{t'},1_{s'}1_{t'},\pm 1_{s'}\mp 1_{t'}} &= \frac{1}{4}\left[D_{s't'} + 2\langle\boldsymbol{\mu}\rangle^C_{1_{s'_1}1_{t'_1}}\langle\boldsymbol{\mu}\rangle^C_{1_{s'_2}1_{t'_2}} - 2\langle\boldsymbol{\mu}\rangle^C_{1_{s'_1}1_{t'_2}}\langle\boldsymbol{\mu}\rangle^C_{1_{s'_2}1_{t'_1}}\right] \\
D^S_{1_{s'}1_{t'},1_{s'}1_{t'},\pm 1_{s'}0_{t'}} &= \frac{1}{2}\left[D^C_{1_{s'_1}1_{t'_3}} + D^C_{1_{s'_2}1_{t'_3}}\right] \\
D^S_{1_{s'}1_{t'},1_{s'}1_{t'},0_{s'}\pm 1_{t'}} &= \frac{1}{2}\left[D^C_{1_{s'_3}1_{t'_1}} + D^C_{1_{s'_3}1_{t'_2}}\right] \\
D^S_{1_{s'}1_{t'},1_{s'}1_{t'},0_{s'}0_{t'}} &= D^C_{1_{s'_3}1_{t'_3}}
\end{aligned} \tag{50}$$

where

$$D_{s't'} = D^C_{1_{s'_1}1_{t'_1}} + D^C_{1_{s'_2}1_{t'_2}} + D^C_{1_{s'_1}1_{t'_2}} + D^C_{1_{s'_2}1_{t'_1}} \tag{51}$$

S7.3.3 Anharmonic Raman activities

$$\begin{aligned}
S^S_{1_{s'}1_{t'},1_{s'}1_{t'},\pm 1_{s'}\pm 1_{t'}} &= \frac{1}{4}\left[S_{s't'} - 2S^C_{1_{s'_1}1_{t'_1};1_{s'_2}1_{t'_2}} + 2S^C_{1_{s'_1}1_{t'_2};1_{s'_2}1_{t'_1}}\right] \\
S^S_{1_{s'}1_{t'},1_{s'}1_{t'},\pm 1_{s'}\mp 1_{t'}} &= \frac{1}{4}\left[S_{s't'} + 2S^C_{1_{s'_1}1_{t'_1};1_{s'_2}1_{t'_2}} - 2S^C_{1_{s'_1}1_{t'_2};1_{s'_2}1_{t'_1}}\right] \\
S^S_{1_{s'}1_{t'},1_{s'}1_{t'},\pm 1_{s'}0_{t'}} &= \frac{1}{2}\left[S^C_{1_{s'_1}1_{t'_3}} + S^C_{1_{s'_2}1_{t'_3}}\right] \\
S^S_{1_{s'}1_{t'},1_{s'}1_{t'},0_{s'}\pm 1_{t'}} &= \frac{1}{2}\left[S^C_{1_{s'_3}1_{t'_1}} + S^C_{1_{s'_3}1_{t'_2}}\right] \\
S^S_{1_{s'}1_{t'},1_{s'}1_{t'},0_{s'}0_{t'}} &= S^C_{1_{s'_3}1_{t'_3}}
\end{aligned} \tag{52}$$

where

$$S_{s't'} = D^C_{1_{s'_1}1_{t'_1}} + S^C_{1_{s'_2}1_{t'_2}} + S^C_{1_{s'_1}1_{t'_2}} + S^C_{1_{s'_2}1_{t'_1}} \tag{53}$$

S7.4 Binary combinations between triply- and doubly-degenerate vibrations

Let us now consider the binary combination bands between a triply-degenerate mode s' and a doubly-degenerate mode t. The combination of one-quantum excitations of s' and t give rise to a set of 6 spherical states $|\psi^S_{1_{s'}1_t,1_{s'},m_{s'},\ell_t}\rangle$ (with $m_{s'} = -1, 0, +1$ and $\ell_t = -1, 1$),

which can be expressed in terms of canonical ones by performing one-by-one products of the matrices $\mathbf{Q}_{1_{s'}}$ and $\mathbf{P}_{1_t}$:

$$\begin{pmatrix} |\psi^S_{1_{s'}1_t,1_{s'},+1_{s'},+1_t}\rangle \\ |\psi^S_{1_{s'}1_t,1_{s'},-1_{s'},-1_t}\rangle \\ |\psi^S_{1_{s'}1_t,1_{s'},+1_{s'},-1_t}\rangle \\ |\psi^S_{1_{s'}1_t,1_{s'},-1_{s'},+1_t}\rangle \\ |\psi^S_{1_{s'}1_t,1_{s'},0_{s'},+1_t}\rangle \\ |\psi^S_{1_{s'}1_t,1_{s'},0_{s'},-1_t}\rangle \end{pmatrix} = -\frac{1}{2} \begin{pmatrix} 1 & -1 & i & i & 0 & 0 \\ 1 & -1 & -i & -i & 0 & 0 \\ 1 & 1 & -i & i & 0 & 0 \\ 1 & 1 & i & -i & 0 & 0 \\ 0 & 0 & 0 & 0 & \sqrt{2} & i\sqrt{2} \\ 0 & 0 & 0 & 0 & \sqrt{2} & -i\sqrt{2} \end{pmatrix} \begin{pmatrix} |\psi^C_{1_{s'_1}1_{t_1}}\rangle \\ |\psi^C_{1_{s'_2}1_{t_2}}\rangle \\ |\psi^C_{1_{s'_1}1_{t_2}}\rangle \\ |\psi^C_{1_{s'_2}1_{t_1}}\rangle \\ |\psi^C_{1_{s'_3}1_{t_1}}\rangle \\ |\psi^C_{1_{s'_3}1_{t_2}}\rangle \end{pmatrix} \tag{54}$$

S7.4.1 Anharmonic energies

$$\begin{aligned} \varepsilon^S_{1_{s'}1_t,1_{s'},\pm1_{s'},\pm1_t} &= \frac{1}{4}\left[\varepsilon_{s't} - 2\langle\psi^C_{1_{s'_1}1_{t_1}}|\tilde{\mathcal{H}}|\psi^C_{1_{s'_2}1_{t_2}}\rangle + 2\langle\psi^C_{1_{s'_1}1_{t_2}}|\tilde{\mathcal{H}}|\psi^C_{1_{s'_2}1_{t_1}}\rangle\right] \\ \varepsilon^S_{1_{s'}1_t,1_{s'},\pm1_{s'},\mp1_t} &= \frac{1}{4}\left[\varepsilon_{s't} + 2\langle\psi^C_{1_{s'_1}1_{t_1}}|\tilde{\mathcal{H}}|\psi^C_{1_{s'_2}1_{t_2}}\rangle + 2\langle\psi^C_{1_{s'_1}1_{t_2}}|\tilde{\mathcal{H}}|\psi^C_{1_{s'_2}1_{t_1}}\rangle\right] \\ \varepsilon^S_{1_{s'}1_t,1_{s'},0_{s'},\pm1_t} &= \frac{1}{2}\left[\varepsilon^C_{1_{s'_3}1_{t_1}} + \varepsilon^C_{1_{s'_3}1_{t_2}}\right] \end{aligned} \tag{55}$$

where

$$\varepsilon_{s't} = \varepsilon^C_{1_{s'_1}1_{t_1}} + \varepsilon^C_{1_{s'_2}1_{t_2}} + \varepsilon^C_{1_{s'_1}1_{t_2}} + \varepsilon^C_{1_{s'_2}1_{t_1}} \tag{56}$$

S7.4.2 Anharmonic dipole strengths

$$\begin{aligned} D^S_{1_{s'}1_t,1_{s'},\pm1_{s'},\pm1_t} &= \frac{1}{4}\left[D_{s't} - 2\langle\boldsymbol{\mu}\rangle^C_{1_{s'_1}1_{t_1}}\langle\boldsymbol{\mu}\rangle^C_{1_{s'_2}1_{t_2}} + 2\langle\boldsymbol{\mu}\rangle^C_{1_{s'_1}1_{t_2}}\langle\boldsymbol{\mu}\rangle^C_{1_{s'_2}1_{t_1}}\right] \\ D^S_{1_{s'}1_t,1_{s'},\pm1_{s'},\mp1_t} &= \frac{1}{4}\left[D_{s't} + 2\langle\boldsymbol{\mu}\rangle^C_{1_{s'_1}1_{t_1}}\langle\boldsymbol{\mu}\rangle^C_{1_{s'_2}1_{t_2}} - 2\langle\boldsymbol{\mu}\rangle^C_{1_{s'_1}1_{t_2}}\langle\boldsymbol{\mu}\rangle^C_{1_{s'_2}1_{t_1}}\right] \\ D^S_{1_{s'}1_t,1_{s'},0_{s'},\pm1_t} &= \frac{1}{2}\left[D^C_{1_{s'_3}1_{t_1}} + D^C_{1_{s'_3}1_{t_2}}\right] \end{aligned} \tag{57}$$

where

$$D_{s't} = D^C_{1_{s'_1}1_{t_1}} + D^C_{1_{s'_2}1_{t_2}} + D^C_{1_{s'_1}1_{t_2}} + D^C_{1_{s'_2}1_{t_1}} \tag{58}$$

S7.4.3 Anharmonic Raman activities

$$\begin{aligned}
S^S_{1_{s'}1_t,1_{s'},\pm 1_{s'},\pm 1_t} &= \frac{1}{4}\left[S_{s't} - 2S^C_{1_{s'_1}1_{t_1};1_{s'_2}1_{t_2}} + 2S^C_{1_{s'_1}1_{t_2};1_{s'_2}1_{t_1}}\right] \\
S^S_{1_{s'}1_t,1_{s'},\pm 1_{s'},\mp 1_t} &= \frac{1}{4}\left[S_{s't} + 2S^C_{1_{s'_1}1_{t_1};1_{s'_2}1_{t_2}} - 2S^C_{1_{s'_1}1_{t_2};1_{s'_2}1_{t_1}}\right] \\
S^S_{1_{s'}1_t,1_{s'},0_{s'},\pm 1_t} &= \frac{1}{2}\left[S^C_{1_{s'_3}1_{t_1}} + S^C_{1_{s'_3}1_{t_2}}\right]
\end{aligned} \tag{59}$$

where

$$S_{s't} = S^C_{1_{s'_1}1_{t_1}} + S^C_{1_{s'_2}1_{t_2}} + S^C_{1_{s'_1}1_{t_2}} + S^C_{1_{s'_2}1_{t_1}} \tag{60}$$

References

(S1) Piccardo, M.; Bloino, J.; Barone, V. Generalized vibrational perturbation theory for rotovibrational energies of linear, symmetric and asymmetric tops: Theory, approximations, and automated approaches to deal with medium-to-large molecular systems. *Int. J. Quantum Chem.* **2015**, *115*, 948–982.

(S2) Plíva, J. Anharmonic constants for degenerate modes of symmetric top molecules. *J. Mol. Spectrosc.* **1990**, *139*, 278–285.

(S3) Willetts, A.; Handy, N. C. The anharmonic constants for a symmetric top. *Chem. Phys. Lett.* **1995**, *235*, 286–290.

(S4) Schuurman, M. S.; Allen, W. D.; von Ragué Schleyer, P.; Schaefer III, H. F. The highly anharmonic BH_5 potential energy surface characterized in the *ab initio* limit. *J. Chem. Phys.* **2005**, *122*, 104302.

(S5) Rosnik, A. M.; Polik, W. F. VPT2+K spectroscopic constants and matrix elements of the transformed vibrational Hamiltonian of a polyatomic molecule with resonances using Van Vleck perturbation theory. *Mol. Phys.* **2014**, *112*, 261–300.

(S6) Krasnoshchekov, S. V.; Isayeva, E. V.; Stepanov, N. F. Criteria for first- and second-order vibrational resonances and correct evaluation of the Darling-Dennison resonance coefficients using the canonical Van Vleck perturbation theory. *J. Chem. Phys.* **2014**, *141*, 234114.

(S7) Henry, L.; Amat, G. The cubic anharmonic potential function of polyatomic molecules. *J. Mol. Spectrosc.* **1961**, *5*, 319–325.

(S8) Henry, L.; Amat, G. The quartic anharmonic potential function of polyatomic molecules. *J. Mol. Spectrosc.* **1965**, *15*, 168–179.

(S9) Amat, G.; Henry, L. SUR LES COEFFICIENTS D'INTERACTION ENTRE LA VIBRATION ET LA ROTATION DANS LES MOLÉCULES POLYATOMIQUES. I PAR. *Cah. Phys.* **1958**, *12*, 273.

(S10) Henry, L.; Amat, G. Sur les coefficients d'intéraction entre la vibration et la rotation dans les molécules polyatomiques. II. *Cah. Phys.* **1960**, *118*, 230–256.

(S11) Bloino, J.; Barone, V. A second-order perturbation theory route to vibrational averages and transition properties of molecules: General formulation and application to infrared and vibrational circular dichroism spectroscopies. *J. Chem. Phys.* **2012**, *136*, 124108.

(S12) Bloino, J.; Biczysko, M.; Barone, V. Anharmonic Effects on Vibrational Spectra Intensities: Infrared, Raman, Vibrational Circular Dichroism, and Raman Optical Activity. *J. Phys. Chem. A* **2015**, *119*, 11862–11874.

(S13) Bloino, J. A VPT2 Route to Near-Infrared Spectroscopy: The Role of Mechanical and Electrical Anharmonicity. *J. Phys. Chem. A* **2015**, *119*, 5269–5287.

(S14) Franke, P. R.; Stanton, J. F.; Douberly, G. E. How to VPT2: Accurate and Intuitive Simulations of CH Stretching Infrared Spectra Using VPT2+ K with Large Effective Hamiltonian Resonance Treatments. *J. Phys. Chem. A* **2021**, *125*, 1301–1324.
